# Genomic view of heavy-ion-induced deletions associated with distribution of essential genes in *Arabidopsis thaliana*

**DOI:** 10.3389/fpls.2024.1352564

**Published:** 2024-04-17

**Authors:** Kotaro Ishii, Yusuke Kazama, Tomonari Hirano, Jeffrey A. Fawcett, Muneo Sato, Masami Yokota Hirai, Fujiko Sakai, Yuki Shirakawa, Sumie Ohbu, Tomoko Abe

**Affiliations:** ^1^ RIKEN Nishina Center for Accelerator-Based Science, Wako, Japan; ^2^ Department of Radiation Measurement and Dose Assessment, Institute for Radiological Science, Quantum Life and Medical Science Directorate, National Institutes for Quantum Science and Technology, Chiba, Japan; ^3^ Department of Bioscience and Biotechnology, Fukui Prefectural University, Eiheiji-cho, Japan; ^4^ Faculty of Agriculture, University of Miyazaki, Miyazaki, Japan; ^5^ RIKEN Interdisciplinary Theoretical and Mathematical Sciences (iTHEMS), Wako, Japan; ^6^ RIKEN Center for Sustainable Resource Science, Yokohama, Japan; ^7^ Graduate School of Bioagricultural Science, Nagoya University, Nagoya, Japan; ^8^ RIKEN Center for Brain Science, Wako, Japan

**Keywords:** heavy-ion beam, linear energy transfer, *Arabidopsis thaliana*, mutagenesis, tandemly arrayed gene, essential gene

## Abstract

Heavy-ion beam, a type of ionizing radiation, has been applied to plant breeding as a powerful mutagen and is a promising tool to induce large deletions and chromosomal rearrangements. The effectiveness of heavy-ion irradiation can be explained by linear energy transfer (LET; keV µm^-1^). Heavy-ion beams with different LET values induce different types and sizes of mutations. It has been suggested that deletion size increases with increasing LET value, and complex chromosomal rearrangements are induced in higher LET radiations. In this study, we mapped heavy-ion beam-induced deletions detected in Arabidopsis mutants to its genome. We revealed that deletion sizes were similar between different LETs (100 to 290 keV μm^-1^), that their upper limit was affected by the distribution of essential genes, and that the detected chromosomal rearrangements avoid disrupting the essential genes. We also focused on tandemly arrayed genes (TAGs), where two or more homologous genes are adjacent to one another in the genome. Our results suggested that 100 keV µm^-1^ of LET is enough to disrupt TAGs and that the distribution of essential genes strongly affects the heritability of mutations overlapping them. Our results provide a genomic view of large deletion inductions in the Arabidopsis genome.

## 1 Introduction

Plant molecular genetics has advanced through both forward and reverse genetic approaches, and recent technological innovations accelerate its understanding. Genome editing tools has contributed to the progress of reverse genetics in plants and it has become easy to obtain knock-out mutants for target genes (Yin et al., 2017; Zhang et al., 2017). Moreover, large deletions, inversions, and translocations (Enciso-Rodriguez et al., 2019; Schmidt et al., 2019; Beying et al., 2020; Schmidt et al., 2020) can be induced in plant genomes, indicating that chromosomal engineering is also possible by using genome-editing technology. On the other hand, in forward genetics, next generation sequencing (NGS) technology has played important roles for the identification of gene functions in recent years, and causative genes for mutants can be identified by whole genome sequencing in *Arabidopsis thaliana* (Schneeberger et al., 2009; Ashelford et al., 2011; Austin et al., 2011; Uchida et al., 2011; Katano et al., 2016; Koide et al., 2018; Yamatani et al., 2018; Du et al., 2020; Nhat et al., 2021) and rice (Abe et al., 2012a; Fekih et al., 2013; Morita et al., 2019).

Heavy-ion beam, a type of radiation, has been applied to plant breeding as a powerful mutagen (Tanaka et al., 2010; Abe et al., 2012b, 2015, 2021; Ishii et al., 2021; Ma et al., 2021). The effectiveness of heavy-ion irradiation can be explained by linear energy transfer (LET; keV µm^-1^). The LET represents the amount of energy deposited locally by radiation. The LETs of ^60^Co γ-ray and 250 keV X-ray are 0.2 keV µm^-1^ and 2.0 keV µm^-1^, respectively, and are called low LET radiations. By contrast, the LET of a heavy-ion beam is variable and higher than those of γ-ray and X-ray. For instance, in the RIKEN RI-beam factory, LETs for biological research range from 22.5 to 4000 keV µm^-1^ (Ryuto et al., 2008). As the energy from heavy-ion beams with high LETs is deposited more densely on the target than the energy from γ-rays and X-rays, irradiation of the beams efficiently causes double strand breaks on DNA molecules and results in more significant biological effects. Difference in LET values affects efficiency of heavy-ion mutagenesis, with the efficiency being highest at an LET of 30 keV µm^-1^ for C ions in *A. thaliana* (Kazama et al., 2008, 2012). Due to its high efficiency, heavy-ion beams have been applied to forward genetic approaches, resulting in many useful mutants (Maeda et al., 2014; Katano et al., 2016; Aonuma et al., 2021; Nhat et al., 2021; Takeshita et al., 2021; Tojo et al., 2021). The effects of LET on small mutations including single nucleotide polymorphisms have been well described (Li et al., 2017; Ichida et al., 2019; Li et al., 2019; Yang et al., 2019; Hase et al., 2020; Oono et al., 2020; Zheng et al., 2020; Ren et al., 2023). Moreover, several investigations have demonstrated that heavy-ion beams with different LET values induce different types and sizes of mutations; deletion size increased with increasing LET value, and complex chromosomal rearrangements were induced in higher LET radiations (Kazama et al., 2011; Hirano et al., 2012; Kazama et al., 2013; Hirano et al., 2015; Hase et al., 2017; Abe et al., 2021; Morita et al., 2021; Sanjaya et al., 2021). Comparison of induced mutations with the LET of 30 and 290 keV µm^-1^ by genome resequencing revealed that the higher LET tended to induce less (0.5 times) small mutations including single-base substitutions and small indels (<100 bp) and more (4.4 times) large mutations including chromosomal rearrangements or large deletions (≥100 bp) (Kazama et al., 2017). Appropriate choice of the LET value would enable to efficiently induce deletions with on-demand size.

Characterization of plant genomes has proceeded in a wide range of species from various viewpoints. One common feature of plant genomes is their high proportion of tandemly arrayed genes (TAGs), where two or more homologous genes are adjacent to one another in the genome (Rizzon et al., 2006; Jander and Barth, 2007). To carry out functional analysis in members of TAGs, it is necessary to disrupt a set of TAGs due to genetic redundancy. Since it is difficult to accumulate mutations in tightly linked loci through crossing with single mutants, induction of a large deletion covering a TAG region is an effective approach to generate knockout mutants corresponding to the TAGs. For instance, disruption of TAGs was achieved by induction of large deletions using γ-ray irradiation (Morita et al., 2007) as a forward genetic approach and Zinc Finger Nucleases as a reverse genetic approach (Qi et al., 2013). It has been also reported that C-ion or Ar-ion beam with higher LET value at 290 keV μm^-1^ can induce deletions ranging from several hundred bp to several Mbp, which are large enough to disrupt TAGs (Hirano et al., 2012, 2015; Kazama et al., 2018; Abe et al., 2021).

The distribution of essential genes would have contributed to the formation of the plant genome. Many essential genes in Arabidopsis were previously reported (Lloyd et al., 2015; Meinke, 2019). If a mutant possesses a deletion covering an essential gene that is involved in morphogenesis or gametogenesis, the deletion would not be homozygously inherited. Although disruption of essential genes should have effects on hereditary nature and/or size limitation of the induced deletion, the relationship between essential genes and deletion mutations has not been investigated at genome level.

In this study, we used three heavy-ion beams at 100, 200, and 290 keV µm^-1^ and investigated how efficiently the beams induce large deletions in the Arabidopsis genome. We also designed array comparative genomic hybridization (array CGH) for the detection of TAG deletions and examined the effects of LET values on TAG disruptions. Further, we examined the distributions of both homozygously and heterozygously inheritable deletions and compared to that of essential genes in the genome, suggesting that the distribution of essential genes affects the upper limit of sizes of homozygously inheritable deletions. Based on these findings, we provide a genomic view for future studies including functional analysis of genes and mutagenesis.

## 2 Materials and methods

### 2.1 Plant materials and irradiation treatment

Dry seeds of *A. thaliana* ecotype Columbia (Col-0) were irradiated with heavy-ion beams as previously described (Kazama et al., 2008). In short, the seeds were irradiated with C-ion beams with LETs of 100 keV μm^-1^ and 200 keV μm^-1^, and Ar-ion beams with an LET of 290 keV μm^-1^ at doses of 150 Gy, 75 Gy, and 50 Gy, respectively, using the E5 beam line in the RIKEN RI-beam factory. The penetration distances of C-ion beams with LETs of 100 keV μm^-1^ and 200 keV μm^-1^, and Ar-ion beams with an LET of 290 keV μm^-1^ in water were calculated to be 1.0 mm, 0.12 mm, and 6.0 mm, respectively, using the SRIM-2013 code ‘The Stopping and Range of Ions in Matter (SRIM)’ (http://www.srim.org). All LET values were calculated behind seeds. Irradiation doses were determined as they showed around 90% survivals in the M1 generation, which are defined as the most effective doses in heavy-ion-beam mutagenesis (Kazama et al., 2008, 2011). More than 1,000 seeds were irradiated for each condition.

### 2.2 Growth conditions and preparation for mutagenized lines

The irradiated M_1_ seeds were surface-sterilized by dipping in 1% sodium hypochlorite for 10 min, washed five times with autoclaved Milli-Q water (1 mL each time), and incubated on Murashige and Skoog medium supplemented with 3% sucrose and 0.7% agar at 4°C in the dark for 4 d to induce vernalization. Subsequently, the seeds were incubated at 22°C under long-day conditions (16 h light, 8 h dark) with white light from fluorescent lamps at approximately 50-100 µM m^-2^ s^-1^ light intensity. Over 100 seedlings that developed true leaves were transplanted into plastic trays (13 × 9 cm^2^) that contained soil. Two seedlings were planted in each tray and grown at 22°C under long-day conditions in a growth chamber. The M_2_ self-pollinated seeds were collected from each M_1_ plant.

For each M_1_ plant, forty M_2_ plants were grown in the same way as M_1_ plants. When the growing M_2_ plants were transplanted into the plastic trays, their phenotypes were checked and the M_2_ plants were divided into apparently normal plants and mutants; the former showed Col-0 like phenotypes and the latter showed visible mutant phenotypes. From each M_2_ line, one normal plant and one mutant were randomly selected and transplanted into the plastic tray. Phenotypes of the mutants were checked again 30 days and 40 days after the cultivation started. For the lines showing no visible mutant phenotypes, two normal plants were randomly selected and transplanted. M_3_ self-pollinated seed aliquots (40 grains each) derived from each M_2_ plant were sown and their seedlings were grown in the same way as the M_1_ plants (**Supplementary Figure S1**). The M_3_ lines were named according to the following rule: “irradiation type” (Ar-ion beams with an LET of 290 keV μm^-1^: Ar50, C-ion beams with LETs of 100 keV μm^-1^: C100, and 200 keV μm^-1^: C200) - “line identification number in the M_2_ generation” - “phenotype” (N means normal), for example, Ar50-01-N1. After confirmation of their phenotypes when they were in the M_2_ generation, leaves of 40 M_3_ plants were harvested and bulked to be used for DNA extraction, which were used for array CGH. At the same time, leaves of 15 individuals were harvested one by one to extract DNA from each individual, which were used for PCR and qPCR analyses.

### 2.3 Array CGH analysis for tandemly arrayed genes

TAGs were listed based on a previous study (Rizzon et al., 2006). The criteria “Low stringency” was adopted; if sequence similarity between two or more tandemly arrayed genes was 70% or more, they were listed as TAGs. Tandemly arrayed homologous genes having another gene between them were also included. Then, corresponding genes were extracted from the whole-genome sequence of *A. thaliana* (TAIR10, http://www.arabidopsis.org/). For each region having TAGs, 1-kbp upstream to 1-kbp downstream regions were tiled with oligonucleotides (**Supplementary Table S1**). In total, 3,469 genes were mounted on the DNA array. The sequence of the oligonucleotide probes was initiated every 100 bp across the genome sequence, excluding repetitive sequences, and probe lengths ranged from 50 to 75 bp.

DNA was extracted from bulks of M_3_ leaves by using a DNA extraction kit, Mag Extractor Plant Genome (Toyobo, Osaka, Japan), followed by the purification using High Pure PCR Cleanup Micro Kit (Roche Diagnostics GmbH, Mannheim, Germany). The DNA of an M_3_ bulk and that of another M_3_ bulk derived from different irradiation treatment was labelled with Cy5 and Cy3, respectively. Hybridization, washing, and scanning of the array CGH were conducted as previously described (Hirano et al., 2015). The positive signals of deletion were extracted as previously described (Hirano et al., 2015). In brief, for each spot on the array, signal values were calculated as log2 ratios of the Cy3-labeled sample (mutant) versus the Cy5-reference (wild type). Peak detection was performed using the Find Peaks feature in SignalMap software, version 1.9 (Roche NimbleGen Inc.) with the peak window size of 400 or 500 bp and the peak threshold of 35% instead of 10% in the previous study.

### 2.4 Confirmation of deletions with PCR and qPCR

The candidate deletions detected as positive peaks in the array CGH were confirmed by PCR using genomic DNA from the 15 individual M_3_ plants. Two primer sets were used for each candidate deletion (**Supplementary Table S2**). The deletions which were confirmed by PCR were defined as homozygously inherited deletions (**Supplementary Table S2**). For the deletions that were not confirmed by PCR, in which the same DNA fragment as in the wild-type plant was amplified, qPCR was performed on genomic DNA from the seven individual M_3_ plants by using LightCycler and the Universal Probe Library detection format (Roche Diagnostics, Penzberg, Germany). Relative amplification ratios between the deleted and non-deleted regions were calculated by using the ΔΔCp method as previously described (Kazama et al., 2015). One primer set was used for each candidate deletion (**Supplementary Table S3**). Deletions determined by qPCR in at least one of the seven siblings to have half the amount of DNA were defined as heterozygously inherited deletions. (**Supplementary Table S3**).

### 2.5 Resequencing of the mutant genome

Genomic DNA was extracted from the collected leaves of 10-15 M_3_ plants using Extractor Plant Genome (Toyobo), followed by the purification using High Pure PCR Cleanup Micro Kit (Roche Diagnostics GmbH). The extracted DNA was sequenced using the HiSeq 4000 sequencing system (Illumina Inc., https://www.illumina.com) as described previously (Kazama et al., 2017). The read sequences obtained were analyzed by using AMAP as described previously (Ishii et al., 2016). The detected candidate mutations were visually confirmed by using the Integrative Genomics Viewer (IGV; Robinson et al., 2011). Visualization of genomic locations of the detected deletions, rearrangements, and essential genes were performed using Circos (Krzywinski et al., 2009).

### 2.6 Metabolic profiling by widely targeted metabolome analysis

Seeds of 90 M_3_ lines that were confirmed to possess deletions in this study except for Ar50-76-pl1 and C200-11-N2 lines were used for metabolic profiling (**Supplementary Table S4**). For each line, three replicates were prepared. Metabolic profiling by widely targeted metabolome analysis (single-grain-based) was conducted as previously described (Sawada et al., 2017). Metabolome data matrix with 128 metabolite (**Supplementary Table S5**) intensities obtained by LC-ESI-QqQ-MS analysis (UPLC-TQS, Waters) was generated from the 270 samples: 90 mutant lines × three replicates derived from each individual plant. The missing values of signal intensities and the values less than 10 were set to 10. Metabolites with a signal-to-noise ratio (defined as the ratio of the averaged signal intensity to that of the extraction-solvent control) < 10 in all experimental groups were removed. In addition, metabolites that showed low signal-to-noise ratio in more than 30% of experimental groups were removed, leaving 75 metabolites. The intensities of the 75 metabolites were divided by those of the internal standards (80% methanol, 0.1% formic acid, 16.8 nmol L^-1^ lidocaine, and 105 mol L^-1^ 10-camphorsulfonic), resulting in the metabolic profiles (**Supplementary Table S4**). Further analyses were conducted using MetaboAnalyst 5.0 (Chong et al., 2018). In brief, missing values were replaced by 1/5 of minimum positive values of their corresponding intensities. Then, intensities were normalized by median. Univariate analysis was conducted by one-way Analysis of Variance (ANOVA) (**Supplementary Table S6**). Hierarchical clustering was performed using ‘euclidean’ distance measure and ‘ward.D.’ clustering algorithm.

### 2.7 Listing mutable and essential genes

We merged the list of 510 embryo-defective (EMB) genes (Meinke, 2019) and 705 genes whose homologous deletions are lethal (Lloyd et al., 2015) into a list consisting of 811 essential genes (**Supplementary Table S7**). We cited 1,765 genes that possess mutations (including 10 or more nonsynonymous SNPs, nonsense SNPs, frameshift small indels, full CDS deletions, or partial deletions) in ecotype Landsberg (Lu et al., 2012), i.e., genes that are most likely not essential for *A. thaliana*, and excluded 25 genes (AT1G24340, AT1G30610, AT1G62340, AT2G03870, AT2G15820, AT2G18510, AT2G24840, AT2G28880, AT2G32590, AT2G33160, AT3G05770, AT3G23110, AT3G24560, AT3G55400, AT4G04790, AT4G13750, AT4G16144, AT4G19490, AT4G21100, AT4G27010, AT4G28590, AT5G08080, AT5G24670, AT5G37630, and AT5G39750) included in the essential genes. We defined the consequent 1,740 genes as mutable genes.

### 2.8 Correlation analysis

The reference genome sequence was divided into 1-Mb bins. In each bin, the numbers of overlapping breakpoints of chromosomal rearrangements (**Supplementary Table S8**) and essential genes (**Supplementary Table S7**) were counted. Multiple breakpoints derived from the same mutant line were counted as one. The correlation coefficient was calculated using *cor.test* function implemented in *R* software environment (Ihaka and Gentleman, 1996).

## 3 Results

### 3.1 Deletion detection for tandemly arrayed genes

To investigate the effect of high-LET beam on inducing the deletion of TAGs, the dry seeds of *Arabidopsis thaliana* Col-0 were irradiated by heavy-ion beams with three conditions: C-ion beams with LETs of 100 keV μm^-1^ and 200 keV μm^-1^, and Ar-ion beams with an LET of 290 keV μm^-1^ (see Materials and Method). For each irradiation condition, 96 M_2_ lines were grown (**Table 1**), and 96 M_2_-lines-derived 192 M_3_ lines (two M_3_ lines per one M_2_ line) were used for the array CGH analysis. As a result, 266 putative deletion signals were detected (**Supplementary Table S2**).

**Table 1:** Summary of deletions of TAGs detected by array CGH.

Ion species (LET, dose)	Phenotype	No. of M_2_ lines	No. of deletion detected lines	No. of detected deletion
C (100 keV µm^-1^, 150Gy)	Normal	34	20	24
	Abnormal	62	2	2
	Total	96	22	26
C (200 keV µm^-1^, 75Gy)	Normal	41	18	23
	Abnormal	55	7	11
	Total	96	25	33
Ar (290 keV µm^-1^, 50Gy)	Normal	50	20	27
	Abnormal	46	12	16
	Total	96	31	43
Total	Normal	125	58	74
	Abnormal	163	21	29
	Total	288	79	103

There are two types of deletions induced by heavy-ion irradiation; one is homozygously inherited deletion that shows mendelian inheritance and the other is deletion that is only inherited heterozygously because of homozygous lethality. To confirm these putative deletions that array CGH analysis sometimes false-positively detects, and to investigate inheritance pattern, PCR and qPCR were performed on the siblings of the M_3_ lines. The deletions which were confirmed by PCR were defined as homozygously inherited deletions (**Supplementary Table S2**). For the deletions not confirmed by PCR, qPCR was conducted to identify heterozygously inherited deletions (**Supplementary Table S3**). Deletions in which at least one sibling showed the result “+” (fragments amplified but with one more amplification cycle) or “-” (fragment not amplified) were considered positive for heterozygous inheritance. Reverting to M_2_ lines, 103 deletions were detected in 79 out of 288 lines by array CGH analysis (**Table 1**) which included 163 and 125 M_2_ lines with and without visible phenotypic abnormality, respectively. The number of induced deletions tended to increase of the value of LET, though it was not significant (Kruskal-Wallis test). Considering that the number of ion particles irradiated on seeds was highest in the irradiation with LET of 100 keV μm^-1^, irradiation with higher LET is likely to induce deletions efficiently. One or more deletions were detected in 19% and 36% of the lines with and without visible phenotypic abnormality, respectively, resulting in no significant difference (chi-square test). This suggests that at least deletions on TAGs detected by array CGH analysis were induced regardless of phenotypic abnormality. We focused on the number of deleted TAGs detected by the array CGH analysis. There were three types of disruption on TAGs: loss of only a part of the TAGs (partial), deletion of one set of TAGs, and two or more sets of TAGs loss (**Table 2**). Deletions covering only one TAGs were more likely to be homozygous while those covering two or more TAGs were more likely to be heterozygous (p < 0.01, chi-square test) (**Table 2**). Deletions covering partial TAG were also more likely to be homozygous than those covering two or more TAGs (p < 0.01, chi-square test). The number of deleted TAGs tended to increase with increase of the value of LET, though only the difference between 100 and 290 keV μm^-1^ was significant (p = 0.047, chi-square test).

**Table 2:** Number of TAGs deleted by each deletion.

Ion species (LET)	Zygosity	No. of deleted TAGs (n)			
		0 < n < 1	n = 1	n ≥ 2	Total*
C (100 keV µm^-1^)	Homozygous	6	8	0	18
	Heterozygous	2	1	9	
C (200 keV µm^-1^)	Homozygous	7	7	2	21
	Heterozygous	5	3	9	
Ar (290 keV µm^-1^)	Homozygous	10	9	5	31
	Heterozygous	3	6	11	
Total	Homozygous	23	24	7	70
	Heterozygous	10	10	29	

*Number of deletions containing one or more TAG irrespective of zygosity.

We then asked how the deletions affect the accumulation of metabolites by conducting metabolic profiling of 126 metabolites on 90 out of 94 M_3_ lines possessing deletions (**Supplementary Tables S4, S5**). Seventy-five metabolites showed significantly different intensity between some combinations of the lines (**Supplementary Figure S2**, **Supplementary Table S6**). This suggests that metabolisms of irradiated line were affected by deletions regardless of phenotypic abnormality, or that mutations undetected by array CGH analysis affected the amounts of the metabolites.

### 3.2 Characteristics of homozygous and heterozygous deletions among three LETs

**Figure 1:**
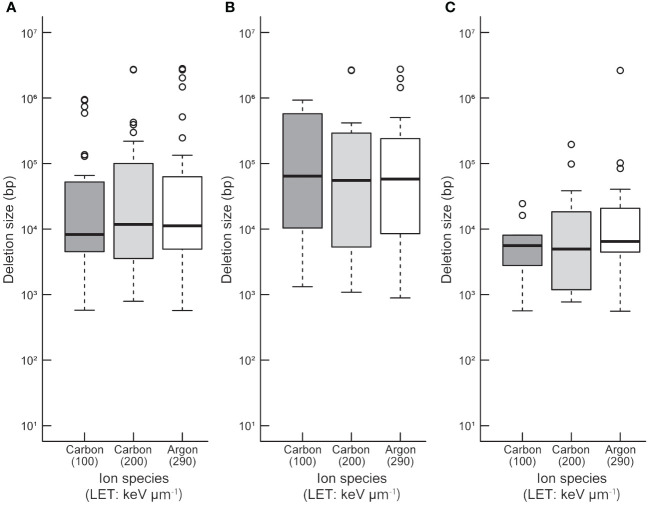
Distribution of deletion size detected by TAG array. Distributions of all **(A)**, heterozygous **(B)**, and homozygous **(C)** deletions induced by each heavy-ion are shown. The median values are indicated by bold horizontal lines in each box. The outliers are indicated by hollow circles.

To compare the sizes between homozygously and heterozygously inherited deletions in the 94 M_3_ lines, both ends of probes showing deletion and both ends of deletions at base-pair levels were used to determine deletion size in the array-based detection (**Supplementary Table S9**). Statistical analysis revealed that there was no significant difference in deletion size between the irradiation treatments with different LETs (p > 0.05; Kruskal-Wallis rank sum test; **Figure 1A**). This tendency did not change when deletion sizes of homozygous and heterozygous deletions were compared separately (**Figures 1B, C**). However, at each irradiation condition, the mean size of heterozygously inherited deletions was significantly larger than that of homozygously inherited deletions (p < 0.05; Wilcoxon rank-sum test; **Figures 1B, C**). This tendency may be attributed to their lengths; longer deletions are more likely to cover essential genes which make them homozygous lethal. This finding raises a question about how the homozygous and heterozygous deletions are distributed in the Arabidopsis genome.

### 3.3 Distribution of deletions and essential genes

**Figure 2:**
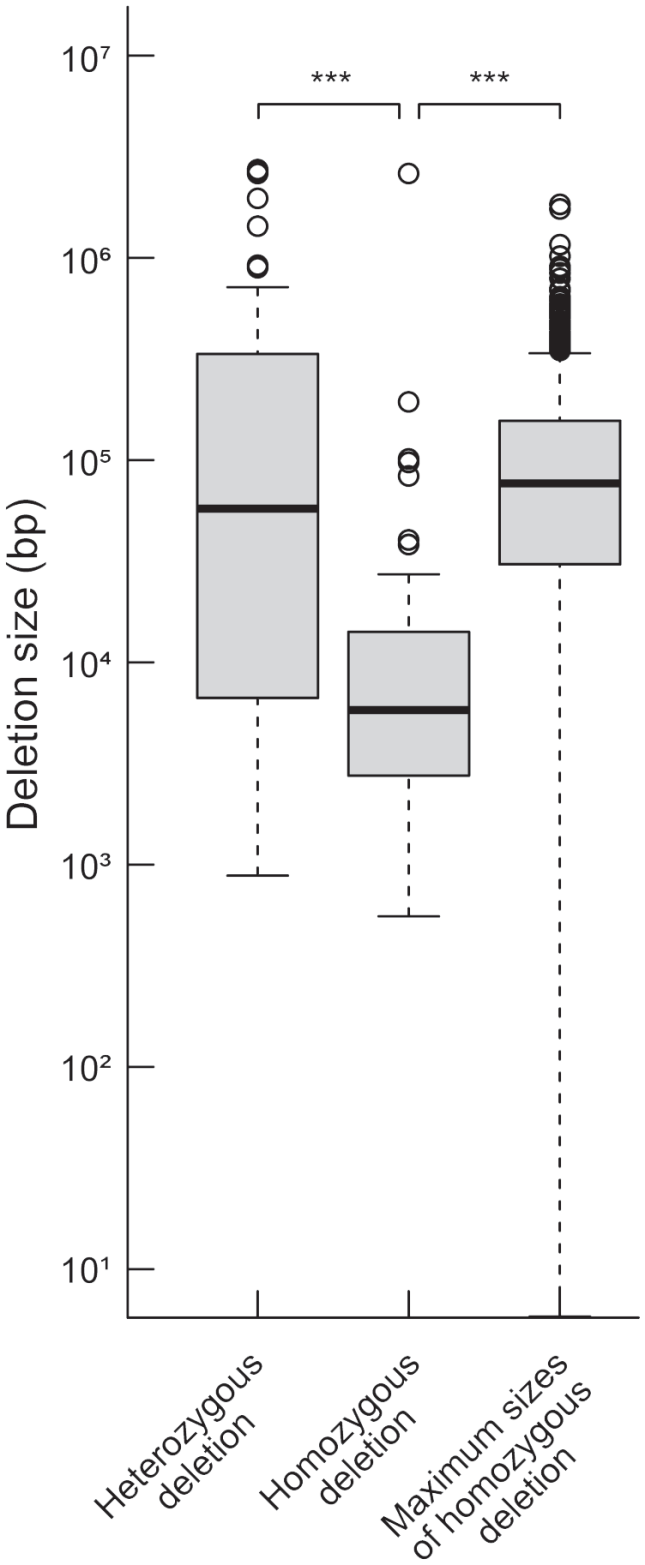
Distributions of heterozygous and homozygous deletions and theoretical maximum sizes of homozygous deletions. The detected deletions were classified into homozygous and heterozygous ones, and distributions of their sizes are shown. Theoretical maximum sizes of homozygous deletions were estimated from distances between the closest pair of essential genes. The median values are indicated by bold horizontal lines in each box. The outliers are indicated by hollow circles. ***; P<0.01 in Wilcoxon rank-sum test.

**Figure 3:**
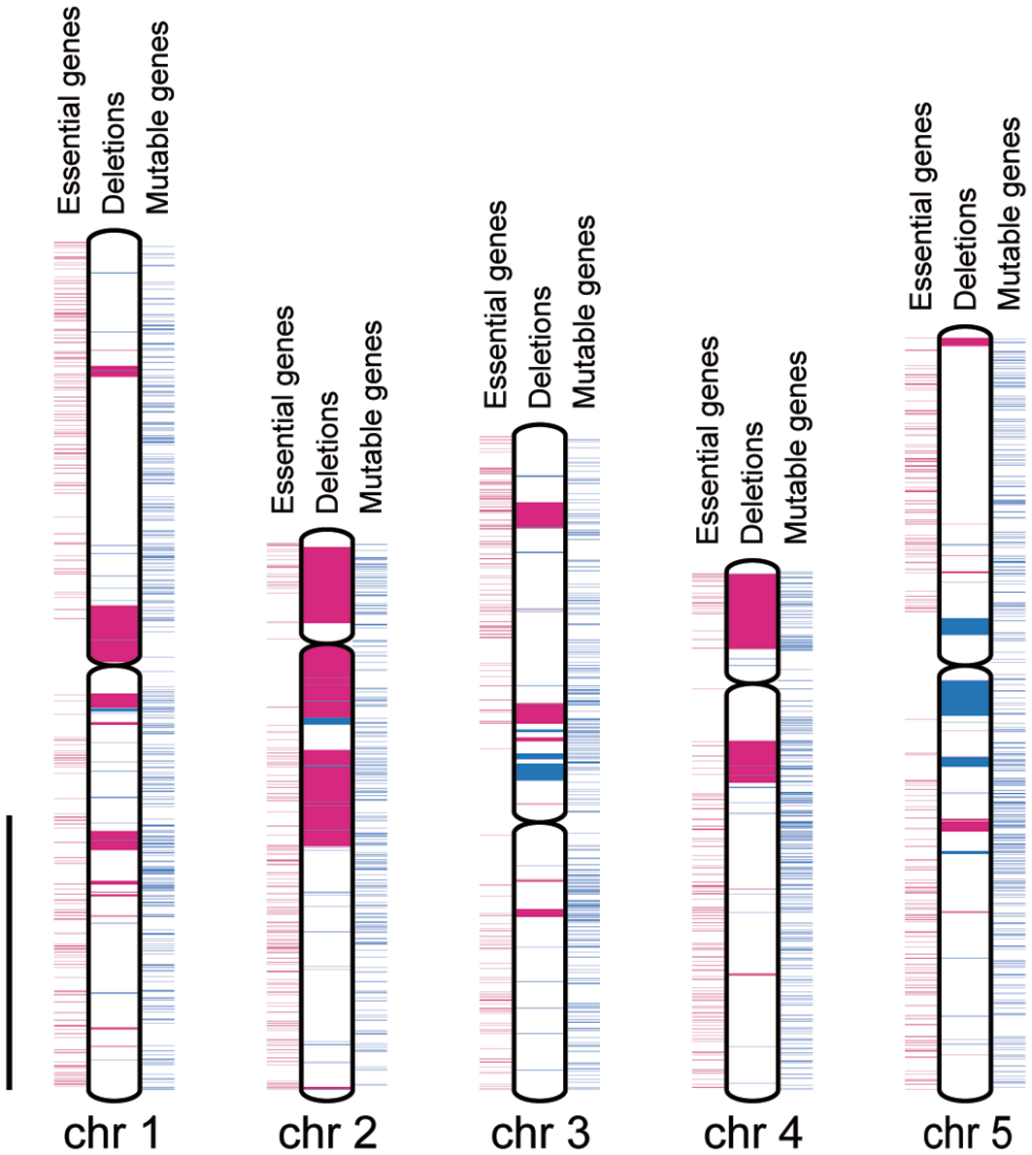
Distributions of genes and deletion mutations. Chromosomes are indicated by white rectangles. Centromeres are indicated by gray spindles. Essential and non-essential genes are indicated by magenta and blue rectangles outside the chromosome, respectively. Heterozygously and homozygously inherited deletions are indicated by magenta and blue rectangles inside the chromosome, respectively. Scale bar = 10 Mbp.

We compared the distances between the closest pair of essential genes as theoretical maximum sizes of homozygous deletions and the sizes of observed homozygous or heterozygous deletions regardless of the LET value. The mean size of the homozygous deletions was significantly smaller than that of the distances between the closest pairs of essential genes (**Figure 2**, P < 0.01; Wilcoxon rank-sum test). This result strongly suggests that the mean size of homozygous deletions seemed to be influenced by the distribution of essential genes. Then we investigated the influence of essential genes on the distributions of homozygous and heterozygous deletions of the mutants. For the heterozygously inherited deletions that are relatively larger than others, genome resequencing was performed to confirm accurate sizes of the deletions as previously described (**Supplementary Table S9**; Hirano et al., 2015; Kazama et al., 2017). We then collected 94 homozygous and 55 heterozygous deletions (> 100 bp) from 94 M_3_ lines possessing deletions isolated in this study and 22 mutant lines previously reported (**Supplementary Table S9**; Hirano et al., 2015; Kazama et al., 2017; Hase et al., 2020; Sanjaya et al., 2021). Next, we investigated the distribution of 1,740 mutable genes. In the 94 homozygous and 55 heterozygous deletions, 593 and 4,061 genes were overlapped, respectively. The mutable genes occupied 10% of the genes in homozygous (62 out of 593) and 6.6% of the genes in heterozygous (270 out of 4,061) deletions, respectively (p < 0.01, chi-square test) (**Figure 3**). Second, we investigated the overlaps of the essential genes with the homozygous or heterozygous deletions (**Figure 3**). No essential gene (0 out of 593) overlapped with homozygous deletions while 2.2% (90 out of 4,061) of the genes in the heterozygous deletions were essential genes (p < 0.01, chi-square test). This contrasting situation can be attributed to the nature of the essential genes that deletions including them cannot be inherited homozygously. These results suggest that the distribution of essential genes affects the upper limit of the deletion size (**Figure 2**).

### 3.4 Location of breakpoints and essential genes

**Figure 4:**
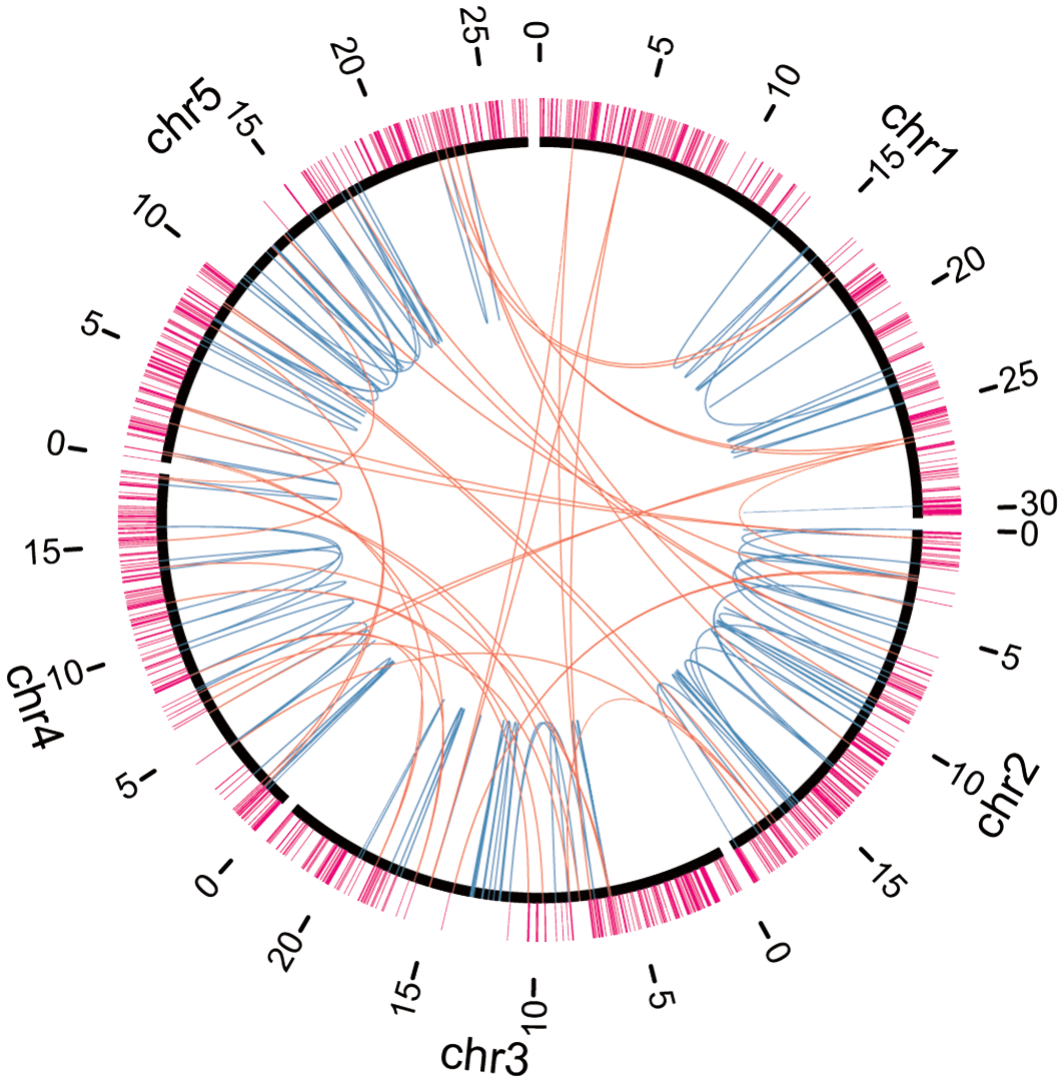
Distributions of essential genes and chromosomal rearrangements. Chromosomes are indicated by black arcs. Essential genes are indicated by magenta lines outside chromosomes. Breakpoint junctions of chromosomal rearrangements and large (≥ 100 bp) deletions listed in **Supplementary Table S8** are indicated by orange (inter-chromosomal) or light blue (intra-chromosomal) arcs inside chromosomes.

Through whole-genome mutation analysis, we and other groups have identified chromosomal rearrangements as well as deletions induced by heavy-ion-beam irradiation (Hirano et al., 2015; Kazama et al., 2017; Hase et al., 2020; Sanjaya et al., 2021). To investigate whether the locations of the breakpoints were influenced by essential genes, we compared the locations of the 532 breakpoints detected by NGS-based analysis in this study and the previous studies (**Supplementary Table S7**; Hirano et al., 2015; Kazama et al., 2017; Hase et al., 2020; Sanjaya et al., 2021) and those of essential genes (**Figure 4**). Almost all of the breakpoints did not overlap with the essential genes, except eight breakpoints: five were heterozygous deletions or chromosomal rearrangements with breakpoints overlapping the genes AT1G63160, AT4G03430, and AT4G27600, two were homozygous translocations with breakpoints in the 5′-UTR region of AT2G28880 gene, and the other one was a homozygous translocation whose breakpoint was in the coding region of AT4G27600 (*NARA5* gene) which is essential for autotrophic photosynthetic growth (Ogawa et al., 2009). We supposed that the distribution of essential genes gave a bias to inheritance of chromosomal rearrangement. Indeed, the numbers of breakpoints and essential genes in every 1-Mb window of the Arabidopsis genome were found to be negatively correlated (**Supplementary Table S10**) (correlation coefficient r = -0.32, p = 0.00036).

## 4 Discussion

### 4.1 Omics analysis on mutants induced by heavy-ion beams

In the current study, we detected large-scale mutations in the Arabidopsis plants after heavy-ion irradiations. We performed NGS-based mutation analysis on the mutants that possessing heterozygously inherited deletions that were detected by array CGH analysis and were relatively larger than others. Some deletions were only detected through NGS-based analysis (**Supplementary Table S9**). As the deletions were small (< 1 kbp), it is likely that they were not detected by the array CGH analysis, which can only detect deletions that overlap with probe regions. The breakpoints of chromosomal rearrangements, such as inversions, can also be detected through NGS-based analysis. Others were detected only by array CGH analysis, possibly due to resequencing mixed samples of 10-15 M_3_ plants derived from the same M_2_ plants, resulting in the deselection of infrequent mutations. Consequently, the combination of array CGH and NGS-based analyses has enabled comprehensive mutation detection.

We also performed metabolic profiling on 90 M_3_ lines possessing deletions and suggested the influence of deletions on metabolome (**Supplementary Table S6**). A heatmap of hierarchical clustering (**Supplementary Figure S2**) showed that several metabolites were upregulated or downregulated specifically in some lines and clustered in the heatmap. For example, in the Ar50-44-as1 line that showed an abnormal shape of leaves (**Supplementary Figure S1**; mutant ID 15 in **Supplementary Figure S2**), a metabolite cluster containing three metabolites (metabolite IDs 1128, 200005, and 200009) was upregulated. We set relevant keywords corresponding to these metabolites and searched the pathway map of *A. thaliana* in the KEGG PATHWAY database (Kanehisa et al., 2021) for pathways in which these metabolites are involved. These metabolites were commonly involved in the flavonoid biosynthesis pathway (ath00941) (**Supplementary Table S11**). In the Ar50-44-as1 line, a deletion in chromosome 2 was also detected by microarray probes located from 8058304 bp to 8099777 bp. The size of this deletion could be expanded to the positions of adjacent probes leading to a maximum size possible (chr2:7920277-8245220) in which 92 genes were included (**Supplementary Table S12**). We also searched the pathway map of *A. thaliana* in the KEGG PATHWAY database for pathways in which these genes are involved. The gene coding a peroxidase superfamily protein (AT2G18980) was involved in the phenylpropanoid biosynthesis (ath00940) that is connected to the flavonoid biosynthesis pathway (ath00941) via metabolites cinnamoyl-CoA and *p*-coumaroyl-CoA. It can be considered that the upregulation of the metabolite cluster was presumably due to the deletion of AT2G18980 gene though it is uncertain whether this metabolic change affected the phenotype. A series of combinational information of deletions and metabolite intensities of the mutant lines would help functional analysis of the deleted TAG in further studies.

### 4.2 Deletion inheritance influenced by both LET and distribution of essential genes

In the current study, we detected multiple deletions in the Arabidopsis plants after irradiations with LET of 100, 200, and 290 keV μm^-1^. In terms of TAG disruption, heavy-ion-beam irradiation with LETs of ≥100 keV µm^-1^ was enough to disrupt TAGs of about two or three genes without affecting the essential genes. In addition to the frequency and size of deletions, the deletions and rearrangements detected in this study provided information for inheritable rearrangements that did not disrupt any essential genes. This could be a beneficial view in inducing deletions in the plant genome. Recently, mutation analysis has been effectively conducted by using whole genome sequencing and exome sequencing in several plant species (Li et al., 2017; Ichida et al., 2019; Li et al., 2019; Hase et al., 2020; Liu et al., 2023; Yang et al., 2019; Oono et al., 2020; Zheng et al., 2020, 2021; Sun et al., 2022; Ren et al., 2023; Wen et al., 2023; Xiong et al., 2023). By using whole-genome mutation analysis, we previously found a significant difference in mutation spectrum between 30 keV μm^-1^ and 290 keV μm^-1^ heavy-ion-beam irradiations (Hirano et al., 2015; Kazama et al., 2017; Morita et al., 2021). It was suggested that heavy-ion beams with higher values of LET tend to induce larger sizes of mutations. In this study, however, the lengths of deletions were not significantly different by LET in a range of 100-290 keV μm^-1^ (**Figure 1A**), presumably affected by the distribution of essential genes. These results imply that the scale of induced mutation increases with the increasing value of LET but that the upper limit of the scale is subject to the distribution of essential genes. Indeed, the einkorn wheat mutants induced by heavy-ion beams showed deletions over 20 kbp or more, even those induced by carbon-ion irradiation with an LET of 50 keV µm^-1^ (Shitsukawa et al., 2007; Nishiura et al., 2018; Hashimoto et al., 2021). In the 570 Mbp long *Silene latifolia* Y chromosome, C-ion irradiation with an LET of 30 keV µm^-1^ induced large deletions covering more than a quarter of the Y chromosome (Kazama et al., 2016; Krasovec et al., 2019). These findings suggest that such large deletions can be induced by C-ion irradiation with the LETs of 30-50 keV µm^-1^ in the species with long distances between essential genes. These findings were consistent with a classical image that any radiations can induce large deletions effectively. However, upper limits of heritable deletion sizes are largely dependent on the distribution of essential genes and only deletions that do not disrupt the essential genes can be inherited homozygously. Conversely, the ability of the heavy-ion beams with high LETs to induce large deletions with high efficiency in the Arabidopsis genome, in which essential genes are densely distributed, may be due to the ion beam causing localized damage on chromosomes.

### 4.3 Putative essential genes overlapped with heterozygous deletions

We investigated the overlap between the essential genes and the 55 heterozygous deletions and revealed that 21 heterozygous deletions overlapped with essential genes (**Figure 3**). In the remaining 34 heterozygous deletions, 32 deletions included 471 genes. It is possible that some of these genes are essential (**Supplementary Table S13**), or that those deletions are linked with small mutations that disrupt the function of essential genes. It is also possible that a deletion including all genes in some TAG leads to lethality as if the TAG behaves as an essential gene. We compared the theoretical maximum sizes of homozygous deletions and the sizes of observed homozygous or heterozygous deletions (**Figure 2**). This result not only strongly suggests that the mean size of heterozygous deletions also seemed to be influenced by the distribution of essential genes though not as much as homozygous deletions, but also implies that the essential genes of Arabidopsis might not be fully discovered yet. Moreover, deletions of some genes involved in gametogenesis are critical even if they are heterozygous, which would be a factor for the upper limit of sizes of both homozygous and heterozygous deletions. Naito et al. (2005) investigated the transmissibility of various sizes of deletions including *GL1* gene induced in pollen cells (M_1_) and proposed that compared to the small deletions (1-4 bp), large deletions (> 6 Mbp) are not transmitted to progeny (M_2_) because the possibility that genes involved in gamete development or viability are overlapped with increasing size of deletions. Kitamura et al. (2022a) compared mutation spectrum between M_1_ and M_2_ generation after gamma-ray irradiation and revealed that chromosomal rearrangements tend not to be inherited to the next generation. Observations of the heritability of deletions in our study corroborates this hypothesis.

### 4.4 Influences of essential gene on inheritance of chromosome rearrangement

Our result implied that the locations of the essential genes are also influential on the locations of breakpoints (**Figure 4**; **Supplementary Table S10**). As one of the exceptions of the relationship, we found a breakpoint in the coding region of *NARA5* which is essential for autotrophic photosynthetic growth (Ogawa et al., 2009). The translocation was detected in the Ar-57-al1 mutant line that was grown on the medium containing sucrose from germination to young plantlet (Hirano et al., 2015). Therefore, Ar-57-al1 plants would be able to overcome the growing stage when *NARA5* is essential. Except for this, no homozygous breakpoint was located in the coding region of essential genes. The irradiation experiments on the pollen grains of *Cyrtanthus mackenii* showed that C-ion irradiation with an LET of 22.5 keV µm^-1^ is sufficient to induce rearrangements in the chromosomes of the male gametophyte and produces more pronounced biological effects on chromosomal bridge induction than Ar-ions with an LET of 280 keV µm^-1^ during the first cell division (Hirano et al., 2013, 2021). Since almost all chromosomal bridges cannot be transmitted to the next generation, the C-ion irradiation with an LET of 22.5 keV µm^-1^ cannot effectively produce inheritable rearrangements. On the other hand, inheritable chromosomal rearrangements were frequently produced with higher LET radiations (Kazama et al., 2017; Hirano et al., 2022). These results suggest that heavy-ion beams with higher LETs can effectively induce inheritable rearrangements because of localized damage on chromosomes.

Different mutation frequencies after heavy-ion irradiation were also observed between chromosomal locations (Kitamura et al., 2022b). In the current study, the percentage of intra-chromosomal rearrangements including large (≥ 100 bp) deletions in all chromosomal rearrangements was 72%. Ex-TAQing system that can induce chromosomal rearrangements randomly using restriction enzymes has been developed (Tanaka et al., 2020). The percentage of intra-chromosomal rearrangements in all chromosomal rearrangements induced by Ex-TAQing that were detected in the two diploid mutants was 82% (18 out of 22). Both heavy-ion beam and Ex-TAQing tend to induce more intra-chromosomal rearrangements than inter-chromosomal rearrangements (not significantly different by Z-test), which is probably because the heritability of inter-chromosomal rearrangement is less than that of intra-chromosomal rearrangement through cell division.

This study led to the conclusion that the distribution of essential genes affects the heritability of large deletions and chromosomal rearrangements. To create large deletions that are effective in destroying TAGs without deleting essential genes, more dense ionization, for example caused by high-LET heavy-ion beams, would be effective. Although less data of the effects of high-LET irradiations on phenotypic spectrum has been provided than low-LET irradiations or chemical mutagens, the effectiveness of the high-LET irradiations on the induction of large deletions and rearrangements may be a beneficial source for creating a new variety of mutants.

## Data availability statement

The datasets presented in this study can be found in online repositories. The names of the repository/repositories and accession number(s) can be found below: https://www.ddbj.nig.ac.jp/, DRA013459.

## Author contributions

KI: Writing – review & editing, Writing – original draft, Visualization, Investigation, Funding acquisition. YK: Writing – review & editing, Writing – original draft, Investigation, Funding acquisition, Data curation, Conceptualization. TH: Writing – review & editing, Writing – original draft, Investigation, Data curation, Conceptualization. JF: Writing – review & editing, Writing – original draft, Investigation. MS: Writing – review & editing, Investigation. MH: Writing – review & editing, Investigation. FS: Writing – review & editing, Investigation. YS: Writing – review & editing, Investigation. SO: Writing – review & editing, Investigation. TA: Writing – review & editing, Supervision, Funding acquisition, Conceptualization.

## References

[B1] Abe T. Ichida H. Hayashi Y. Morita R. Shirakawa Y. Ishii K.. (2021). “Ion beam mutagenesis - an innovative and effective method for plant breeding and gene discovery,” in Mutation breeding, genetic diversity and crop adaptation to climate change. Eds. Sivasankar S. Ellis T. H. N. Jankuloski L. Ingelbrecht I.(CABI, Oxfordshire, UK), 411–423.

[B2] Abe T. Kazama Y. Hirano T. (2015). Ion beam breeding and gene discovery for function analyses using mutants. Nucl. Phys. News 25, 30–34. doi: 10.1080/10619127.2015.1104130

[B3] Abe A. Kosugi S. Yoshida K. Natsume S. Takagi H. Kanzaki H.. (2012a). Genome sequencing reveals agronomically important loci in rice using MutMap. Nat. Biotechnol. 30, 174–178. doi: 10.1038/nbt.2095 22267009

[B4] Abe T. Ryuto H. Fukunishi N. (2012b). “Ion beam radiation mutagenesis,” in Plant Mutation Breeding and Biotechnology. Eds. Shu Q. Y. Forster B. P. Nakagawa H.(CABI, Oxfordshire, UK), 99–106.

[B5] Aonuma W. Kawamoto H. Kazama Y. Ishii K. Abe T. Kawano S. (2021). Male/female trade-off in hermaphroditic Y-chromosome deletion mutants of the dioecious plant *Silene latifolia* . Cytologia 86, 329–338. doi: 10.1508/cytologia.86.329

[B6] Ashelford K. Eriksson M. E. Allen C. M. D'Amore R. Johansson M. Gould P.. (2011). Full genome re-sequencing reveals a novel circadian clock mutation in *Arabidopsis* . Genome Biol. 12, R28. doi: 10.1186/gb-2011-12-3-r28 21429190 PMC3129678

[B7] Austin R. S. Vidaurre D. Stamatiou G. Breit R. Provart N. J. Bonetta D.. (2011). Next-generation mapping of Arabidopsis genes. Plant J. 67, 715–725. doi: 10.1111/j.1365-313X.2011.04619.x 21518053

[B8] Beying N. Schmidt C. Pacher M. Houben A. Puchta H. (2020). CRISPR–Cas9-mediated induction of heritable chromosomal translocations in *Arabidopsis* . Nat. Plants 6, 638–645. doi: 10.1038/s41477-020-0663-x 32451449

[B9] Chong J. Soufan O. Li C. Caraus I. Li S. Bourque G.. (2018). MetaboAnalyst 4.0: towards more transparent and integrative metabolomics analysis. Nucleic Acids Res. 46, W486–W494. doi: 10.1093/nar/gky310 29762782 PMC6030889

[B10] Du Y. Hase Y. Satoh K. Shikazono N. (2020). Characterization of gamma irradiation-induced mutations in Arabidopsis mutants deficient in non-homologous end joining. J. Radiat. Res. 61, 639–647. doi: 10.1093/jrr/rraa059 32766789 PMC7482170

[B11] Enciso-Rodriguez F. Manrique-Carpintero N. C. Nadakuduti S. S. Buell C. R. Zarkam D. Douches D. (2019). Overcoming self-incompatibility in diploid potato using CRISPR-Cas9. Front. Plant Sci. 10. doi: 10.3389/fpls.2019.00376 PMC6454193 31001300

[B12] Fekih R. Takagi H. Tamiru M. Abe A. Natsume S. Yaegashi H.. (2013). MutMap+: genetic mapping and mutant identification without crossing in rice. PloS One 8, e68529. doi: 10.1371/journal.pone.0068529 23874658 PMC3707850

[B13] Hase Y. Nozawa S. Narumi I. Oono Y. (2017). Effects of ion beam irradiation on size of mutant sector and genetic damage in Arabidopsis. Nucl. Instrum. Methods Phys. Res. B 391, 14–19. doi: 10.1016/j.nimb.2016.11.023

[B14] Hase Y. Satoh K. Seito H. Oono Y. (2020). Genetic consequences of acute/chronic gamma and carbon ion irradiation of *arabidopsis thaliana* . Front. Plant Sci. 11. doi: 10.3389/fpls.2020.00336 PMC7113374 32273879

[B15] Hashimoto K. Kazama Y. Ichida H. Abe T. Murai K. (2021). Einkorn wheat (*Triticum monococcum*) mutant extra-early flowering 4, generated by heavy-ion beam irradiation, has a deletion of the *LIGHT-REGULATED WD1* homolog. Cytologia 86, 297–302. doi: 10.1508/cytologia.86.297

[B16] Hirano T. Kazama Y. Ishii K. Ohbu S. Shirakawa Y. Abe T. (2015). Comprehensive identification of mutations induced by heavy-ion beam irradiation in *Arabidopsis thaliana* . Plant J. 82, 93–104. doi: 10.1111/tpj.12793 25690092

[B17] Hirano T. Kazama Y. Kunitake H. Abe T. (2022). Mutagenic effects of heavy-ion beam irradiation to plant genome. Cytologia 87, 3–6. doi: 10.1508/cytologia.87.3

[B18] Hirano T. Kazama Y. Ohbu S. Shirakawa Y. Liu Y. Kambara T.. (2012). Molecular nature of mutations induced by high-LET irradiation with argon and carbon ions in *Arabidopsis thaliana* . Mutat. Res. 735, 19–31. doi: 10.1016/j.mrfmmm.2012.04.010 22579628

[B19] Hirano T. Matsuyama Y. Hanada A. Hayashi Y. Abe T. Kunitake H. (2021). DNA damage response of *Cyrtanthus mackenii* male gametes following argon ion beam irradiation. Cytologia 86, 311–315. doi: 10.1508/cytologia.86.311

[B20] Hirano T. Takagi K. Hoshino Y. Abe T. (2013). DNA damage response in male gametes of *Cyrtanthus mackenii* during pollen tube growth. AoB Plants 5, plt004. doi: 10.1093/aobpla/plt004 23550213 PMC3583183

[B21] Ichida H. Morita R. Shirakawa Y. Hayashi Y. Abe T. (2019). Targeted exome sequencing of unselected heavy-ion beam-irradiated populations reveals less-biased mutation characteristics in the rice genome. Plant J. 98, 301–314. doi: 10.1111/tpj.14213 30584677 PMC6850588

[B22] Ihaka R. Gentleman R. (1996). R: a language for data analysis and graphics. J. Comput. Graph. Stat. 5, 299–314. doi: 10.2307/1390807

[B23] Ishii K. Kawano S. Abe T. (2021). Creation of green innovation and functional gene analyses using heavy-ion beam breeding. Cytologia 86, 273–274. doi: 10.1508/cytologia.86.273

[B24] Ishii K. Kazama Y. Hirano T. Hamada M. Ono Y. Yamada M.. (2016). AMAP: a pipeline for whole-genome mutation detection in *Arabidopsis thaliana* . Genes Genet. Syst. 91, 229–233. doi: 10.1266/ggs.15-00078 27452041

[B25] Jander G. Barth C. (2007). Tandem gene arrays: a challenge for functional genomics. Trends Plant Sci. 12, 203–210. doi: 10.1016/j.tplants.2007.03.008 17416543

[B26] Kanehisa M. Furumichi M. Sato Y. Ishiguro-Watanabe M. Tanabe M. (2021). KEGG: integrating viruses and cellular organisms. Nucleic Acids Res. 49, D545–D551. doi: 10.1093/nar/gkaa970 33125081 PMC7779016

[B27] Katano M. Takahashi K. Hirano T. Kazama Y. Abe T. Tsukaya H.. (2016). Suppressor screen and phenotype analyses revealed an emerging role of the monofunctional peroxisomal enoyl-CoA hydratase 2 in compensated cell enlargement. Front. Plant Sci. 7. doi: 10.3389/fpls.2016.00132 PMC4756126 26925070

[B28] Kazama Y. Hirano T. Abe T. Matsunaga S. (2018). Chromosomal rearrangement: from induction by heavy-ion irradiation to in *vivo* engineering by genome editing. Cytologia 83, 125–128. doi: 10.1508/cytologia.83.125

[B29] Kazama Y. Hirano T. Ishii K. Yamada M. Shirakawa Y. Ohbu S.. (2015). Rapid screening of heavy-ion-induced large deletion mutants by using quantitative real-time PCR in *Arabidopsis thaliana* . RIKEN Accel. Prog. Rep. 48, 307.

[B30] Kazama Y. Hirano T. Nishihara K. Ohbu S. Shirakawa Y. Abe T. (2013). Effect of high-LET Fe-ion beam irradiation on mutation induction in *Arabidopsis thaliana* . Genes Genet. Syst. 88, 189–197. doi: 10.1266/ggs.88.189 24025247

[B31] Kazama Y. Hirano T. Saito H. Liu Y. Ohbu S. Hayashi Y.. (2011). Characterization of highly efficient heavy-ion mutagenesis in *Arabidopsis thaliana* . BMC Plant Biol. 11, 161. doi: 10.1186/1471-2229-11-161 22085561 PMC3261129

[B32] Kazama Y. Ishii K. Aonuma W. Ikeda T. Kawamoto H. Koizumi A.. (2016). A new physical mapping approach refines the sex-determining gene positions on the *Silene latifolia* Y-chromosome. Sci. Rep. 6, 18917. doi: 10.1038/srep18917 26742857 PMC4705512

[B33] Kazama Y. Ishii K. Hirano T. Wakana T. Yamada M. Ohbu S.. (2017). Different mutational function of low- and high-linear energy transfer heavy-ion irradiation demonstrated by whole-genome resequencing of Arabidopsis mutants. Plant J. 92, 1020–1030. doi: 10.1111/tpj.13738 29024116

[B34] Kazama Y. Ma L. Hirano T. Ohbu S. Shirakawa Y. Hatakeyama S.. (2012). Rapid evaluation of effective linear energy transfer in heavy-ion mutagenesis of *Arabidopsis thaliana* . Plant Biotechnol. (Tokyo) 29, 441–445. doi: 10.5511/plantbiotechnology.12.0921a

[B35] Kazama Y. Saito H. Yamamoto Y. Y. Hayashi Y. Ichida H. Ryuto H.. (2008). LET-dependent effects of heavy-ion beam irradiation in *Arabidopsis thaliana* . Plant Biotechnol. (Tokyo) 25, 113–117. doi: 10.5511/plantbiotechnology.25.113

[B36] Kitamura S. Hirata S. Satoh K. Inamura R. Narumi I. Oono Y. (2022b). Development of a simple multiple mutation detection system using seed-coat flavonoid pigments in irradiated Arabidopsis M_1_ plants. Sci. Rep. 12, 22467. doi: 10.1038/s41598-022-26989-z 36577797 PMC9797493

[B37] Kitamura S. Satoh K. Oono Y. (2022a). Detection and characterization of genome-wide mutations in M1 vegetative cells of gamma-irradiated Arabidopsis. PloS Genet. 18, e1009979. doi: 10.1371/journal.pgen.1009979 35051177 PMC8775353

[B38] Koide Y. Ogino A. Yoshikawa T. Kitashima Y. Saito N. Kanaoka Y.. (2018). Lineage-specific gene acquisition or loss is involved in interspecific hybrid sterility in rice. PNAS 115, E1955–E1962. doi: 10.1073/pnas.1711656115 29444864 PMC5834674

[B39] Krasovec M. Kazama Y. Ishii K. Abe T. Filatov D. A. (2019). Immediate dosage compensation is triggered by the deletion of Y-linked genes in *Silene latifolia* . Curr. Biol. 29, 2214–2221.e4. doi: 10.1016/j.cub.2019.05.060 31231053 PMC6616318

[B40] Krzywinski M. Schein J. Birol I. Connors J. Gascoyne R. Horsman D.. (2009). Circos: an information aesthetic for comparative genomics. Genome Res. 19, 1639–1645. doi: 10.1101/gr.092759.109 19541911 PMC2752132

[B41] Li G. Jain R. Chern M. Pham N. T. Martin J. A. Wei T.. (2017). The sequences of 1504 mutants in the model rice variety Kitaake facilitate rapid functional genomic studies. Plant Cell 29, 1218–1231. doi: 10.1105/tpc.17.00154 28576844 PMC5502455

[B42] Li F. Shimizu A. Nishio T. Tsutsumi N. Kato H. (2019). Comparison and characterization of mutations induced by gamma-ray and carbon-ion irradiation in rice (*Oryza sativa* L.) using whole-genome resequencing. G3-Genes Genom. Genet. 9, 3743–3751. doi: 10.1534/g3.119.400555 PMC6829151 31519747

[B43] Liu J. Zhao G. Geng J. Geng Z. Dou H. Liu X. An Z.. (2023). Genome-wide analysis of mutations induced by carbon ion beam irradiation in cotton. Front. Plant Sci. 14, 1056662. doi: 10.3389/fpls.2023.1056662 36875607 PMC9978701

[B44] Lloyd J. P. Seddon A. E. Moghe G. D. Simenc M. C. Shiu S. (2015). Characteristics of plant essential genes allow for within- and between-species prediction of lethal mutant phenotypes. Plant Cell 27, 2133–2147. doi: 10.1105/tpc.15.00051 26286535 PMC4568498

[B45] Lu P. Han X. Qi J. Yang J. Wijeratne A. J. Li T.. (2012). Analysis of *Arabidopsis* genome-wide variations before and after meiosis and meiotic recombination by resequencing Landsberg *erecta* and all four products of a single meiosis. Genome Res. 22, 508–518. doi: 10.1101/gr.127522.111 22106370 PMC3290786

[B46] Ma L. Kong F. Sun K. Wang T. Guo T. (2021). From classical radiation to modern radiation: past, present, and future of radiation mutation breeding. Front. Public Health 9. doi: 10.3389/fpubh.2021.768071 PMC8725632 34993169

[B47] Maeda S. Gunji S. Hanai K. Hirano T. Kazama Y. Ohbayashi I.. (2014). The conflict between cell proliferation and expansion primarily affects stem organogenesis in Arabidopsis. Plant Cell Physiol. 55, 1994–2007. doi: 10.1093/pcp/pcu131 25246492

[B48] Meinke D. W. (2019). Genome-wide identification of *EMBRYO-DEFECTIVE* (*EMB*) genes required for growth and development in Arabidopsis. New Phytol. 226, 306–325. doi: 10.1111/nph.16071 31334862

[B49] Morita R. Ichida H. Hayashi Y. Ishii K. Shirakawa Y. Usuda-Kogure S.. (2021). Responsible gene analysis of phenotypic mutants revealed the linear energy transfer (LET)-dependent mutation spectrum in rice. Cytologia 86, 303–309. doi: 10.1508/cytologia.86.303

[B50] Morita R. Ichida H. Ishii K. Hayashi Y. Abe H. Shirakawa Y.. (2019). *LONG GRAIN 1*: a novel gene that regulates grain length in rice. Mol. Breed. 39, 135. doi: 10.1007/s11032-019-1032-1

[B51] Morita R. Kusaba M. Iida S. Nishio T. Nishimura M. (2007). Knockout of glutelin genes which form a tandem array with a high level of homology in rice by gamma irradiation. Genes Genet. Syst. 82, 321–327. doi: 10.1266/ggs.82.321 17895583

[B52] Naito K. Kusaba M. Shikazono N. Takano T. Tanaka A. Tanisaka T.. (2005). Transmissible and nontransmissible mutations induced by irradiating *Arabidopsis thaliana* pollen with gamma-rays and carbon ions. Genetics 169, 881–889. doi: 10.1534/genetics.104.033654 15371348 PMC1449103

[B53] Nhat V. Q. Kazama Y. Ishii K. Ohbu S. Kunitake H. Abe T.. (2021). Double mutant analysis with the large flower mutant, ohbana1, to explore the regulatory network controlling the flower and seed sizes in *Arabidopsis thaliana* . Plants 10, 1881. doi: 10.3390/plants10091881 34579413 PMC8473154

[B54] Nishiura A. Kitagawa S. Matsumura M. Kazama Y. Abe T. Mizuno N.. (2018). An early-flowering einkorn wheat mutant with deletions of *PHYTOCLOCK 1/LUX ARRHYTHMO* and *VERNALIZATION 2* exhibits a high level of *VERNALIZATION 1* expression induced by vernalization. J. Plant Physiol. 222, 28–38. doi: 10.1016/j.jplph.2018.01.002 29367015

[B55] Ogawa T. Nishimura K. Aoki T. Takase H. Tomizawa K. Ashida H.. (2009). A phosphofructokinase B-type carbohydrate kinase family protein, NARA5, for massive expressions of plastid-encoded photosynthetic genes in Arabidopsis. Plant Physiol. 151, 114–112. doi: 10.1104/pp.109.139683 19587101 PMC2736000

[B56] Oono Y. Ichida H. Morita R. Nozawa S. Satoh K. Shimizu A.. (2020). Genome sequencing of ion-beam-induced mutants facilitates detection of candidate genes responsible for phenotypes of mutants in rice. Mutat. Res. 821, 111691. doi: 10.1016/j.mrfmmm.2020.111691 32171089

[B57] Qi Y. Li X. Zhang Y. Starker C. G. Baltes N. J. Zhang F.. (2013). Targeted deletion and inversion of tandemly arrayed genes in *Arabidopsis thaliana* using zinc finger nucleases. G3-Genes Genom. Genet. 3, 1707–1715. doi: 10.1534/g3.113.006270 PMC3789795 23979943

[B58] Ren W. Wang H. Du Y. Li Y. Feng Z. Zhou X.. (2023). Multi-generation study of heavy ion beam-induced mutations and agronomic trait variations to accelerate rice breeding. Front. Plant Sci. 14. doi: 10.3389/fpls.2023.1213807 PMC10322207 37416884

[B59] Rizzon C. Ponger L. Gaut B. S. (2006). Striking similarities in the genomic distribution of tandemly arrayed genes in *Arabidopsis* and rice. PloS Comput. Biol. 2, e115. doi: 10.1371/journal.pcbi.0020115 16948529 PMC1557586

[B60] Robinson J. T. Thorvaldsdottir H. Winckler W. Guttman M. Lander E. S. Getz G.. (2011). Integrative genomics viewer. Nat. Biotechnol. 29, 24–26. doi: 10.1038/nbt.1754 21221095 PMC3346182

[B61] Ryuto H. Fukunishi N. Hayashi Y. Ichida H. Abe T. Kase M.. (2008). Heavy-ion beam irradiation facility for biological samples in RIKEN. Plant Biotechnol. (Tokyo) 25, 119–122. doi: 10.5511/plantbiotechnology.25.119

[B62] Sanjaya A. Kazama Y. Ishii K. Muramatsu R. Kanamaru K. Ohbu S.. (2021). An argon-ion-induced pale green mutant of *Arabidopsis* exhibiting rapid disassembly of mesophyll chloroplast grana. Plants 10, 848. doi: 10.3390/plants10050848 33922223 PMC8145761

[B63] Sawada Y. Tsukaya H. Li Y. Sato M. Kawade K. Hirai M. Y. (2017). A novel method for single-grain-based metabolic profiling of Arabidopsis seed. Metabolomics 13, 75. doi: 10.1007/s11306-017-1211-1

[B64] Schmidt C. Fransz P. Rönspies M. Dreissig S. Fuchs J. Heckmann S.. (2020). Changing local recombination patterns in Arabidopsis by CRISPR/Cas mediated chromosome engineering. Nat. Commun. 11, 4418. doi: 10.1038/s41467-020-18277-z 32887885 PMC7474074

[B65] Schmidt C. Pacher M. Puchta H. (2019). Efficient induction of heritable inversions in plant genomes using the CRISPR/Cas system. Plant J. 98, 577–589. doi: 10.1111/tpj.14322 30900787

[B66] Schneeberger K. Ossowski S. Lanz C. Juul T. Petersen A. H. Nielsen K. L.. (2009). SHOREmap: simultaneous mapping and mutation identification by deep sequencing. Nat. Methods 6, 550–551. doi: 10.1038/nmeth0809-550 19644454

[B67] Shitsukawa N. Ikari C. Shimada S. Kitagawa S. Sakamoto K. Saito H.. (2007). The einkorn wheat (*Triticum monococcum*) mutant, *maintained vegetative phase*, is caused by a deletion in the *VRN1* gene. Genes Genet. Syst. 82, 167–170. doi: 10.1266/ggs.82.167 17507783

[B68] Sun K. Li D. Xia A. Zhao H. Wen Q. Jia S.. (2022). Targeted identification of Rice grain-associated gene allelic variation through mutation induction, targeted sequencing, and whole genome sequencing combined with a mixed-samples strategy. Rice 15, 57. doi: 10.1186/s12284-022-00603-2 36326973 PMC9633910

[B69] Takeshita T. Takita K. Ishii K. Kazama Y. Abe T. Kawano S. (2021). Robust mutants isolated through heavy-ion-beam irradiation and endurance screening in the green alga *Haematococcus pluvialis* . Cytologia 86, 283–289. doi: 10.1508/cytologia.86.283

[B70] Tanaka H. Muramoto N. Sugimoto H. Oda A. H. Ohta K. (2020). Extended TAQing system for large-scale plant genome reorganization. Plant J. 103, 2139–2150. doi: 10.1111/tpj.14888 32579240

[B71] Tanaka A. Shikazono N. Hase Y. (2010). Studies on biological effects of ion beams on lethality, molecular nature of mutation, mutation rate, and spectrum of mutation phenotype for mutation breeding in higher plants. J. Radiat. Res. 51, 223–233. doi: 10.1269/jrr.09143 20505261

[B72] Tojo H. Nakamura A. Ferjani A. Kazama Y. Abe T. Iida H. (2021). A method enabling comprehensive isolation of Arabidopsis mutants exhibiting unusual root mechanical behavior. Front. Plant Sci. 12. doi: 10.3389/fpls.2021.646404 PMC7966703 33747026

[B73] Uchida N. Sakamoto T. Kurata T. Tasaka M. (2011). Identification of EMS-induced causal mutations in a non-reference *Arabidopsis thaliana* accession by whole genome sequencing. Plant Cell Physiol. 52, 716–722. doi: 10.1093/pcp/pcr029 21398646

[B74] Wen X. Li J. Yang F. Zhang X. Li Y. (2023). Exploring the effect of high-energy heavy ion beam on Rice genome: transposon activation. Genes 14, 2178. doi: 10.3390/genes14122178 38137000 PMC10742395

[B75] Xiong H. Guo H. Fu M. Xie Y. Zhao L. Gu J.. (2023). A large-scale whole-exome sequencing mutant resource for functional genomics in wheat. Plant Biotechnol. J. 21, 2047–2056. doi: 10.1111/pbi.14111 37401008 PMC10502753

[B76] Yamatani H. Kohzuma K. Nakano M. Takami T. Kato Y. Hayashi Y.. (2018). Impairment of Lhca4, a subunit of LHCI, causes high accumulation of chlorophyll and the stay-green phenotype in rice. J. Exp. Bot. 69, 1027–1035. doi: 10.1093/jxb/erx468 29304198 PMC6019047

[B77] Yang G. Luo W. Zhang J. Yan X. Du Y. Zhou L.. (2019). Genome-wide comparisons of mutations induced by carbon-ion beam and gamma-rays irradiation in rice *via* resequencing multiple mutants. Front. Plant Sci. 10. doi: 10.3389/fpls.2019.01514 PMC6892775 31850019

[B78] Yin K. Gao C. Qiu J. (2017). Progress and prospects in plant genome editing. Nat. Plants 3, 17107. doi: 10.1038/nplants.2017.107 28758991

[B79] Zhang K. Raboanatahiry N. Zhu B. Li M. (2017). Progress in genome editing technology and its application in plants. Front. Plant Sci. 8. doi: 10.3389/fpls.2017.00177 PMC5306361 28261237

[B80] Zheng Y. Li S. Huang J. Fu H. Zhou L. Furusawa Y.. (2020). Mutagenic effect of three ion beams on rice and identification of heritable mutations by whole genome sequencing. Plants 9, 551. doi: 10.3390/plants9050551 32357388 PMC7284785

[B81] Zheng Y. Li S. Huang J. Fu H. Zhou L. Furusawa Y.. (2021). Identification and characterization of inheritable structural variations induced by ion beam radiations in rice. Mutat. Res. 823, 111757. doi: 10.1016/j.mrfmmm.2021.111757 34271440

