# Supplementary material for: Genomic view of heavy-ion-induced deletions associated with distribution of essential genes in Arabidopsis thaliana
Source: Front Plant Sci. 2024 Apr 17;15:1352564. doi: 10.3389/fpls.2024.1352564 (PMC11061394; doi:10.3389/fpls.2024.1352564)
Supplement: Supplementary file 1 [file Image_1.pdf]

Ar50-6-N2

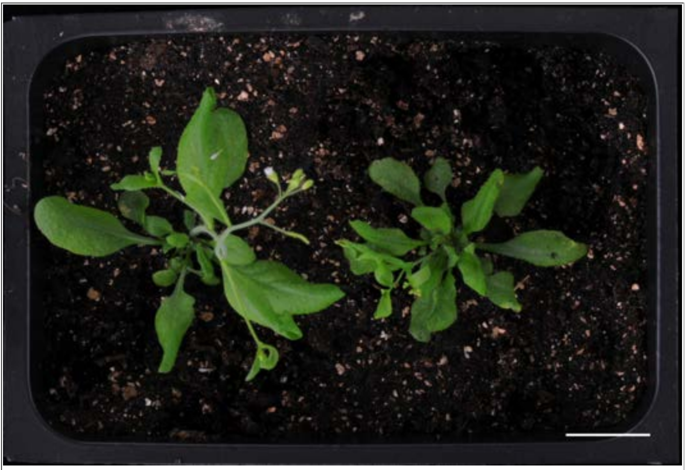
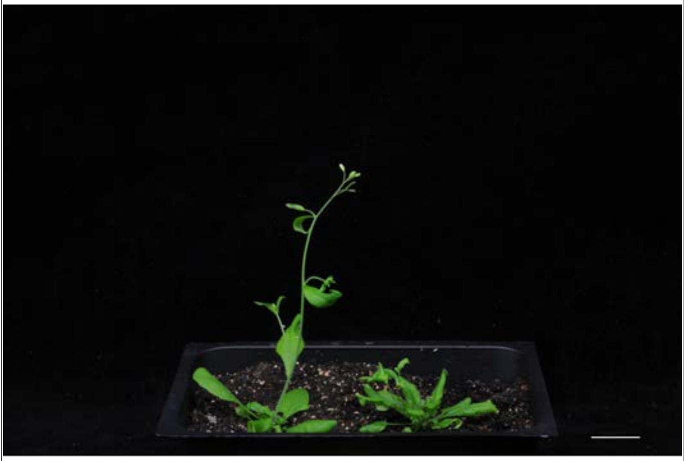

Ar50-12-pg1

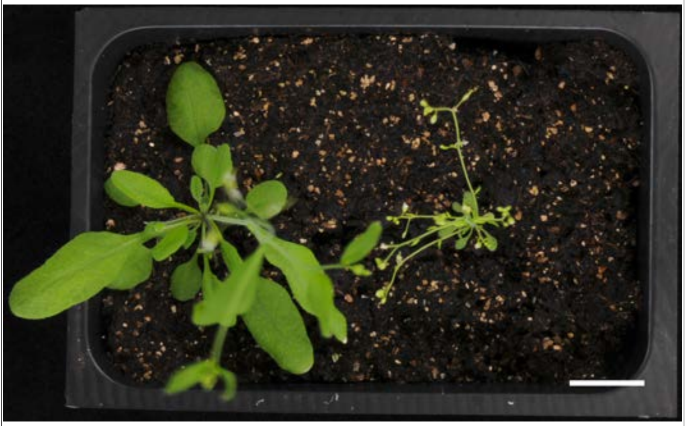
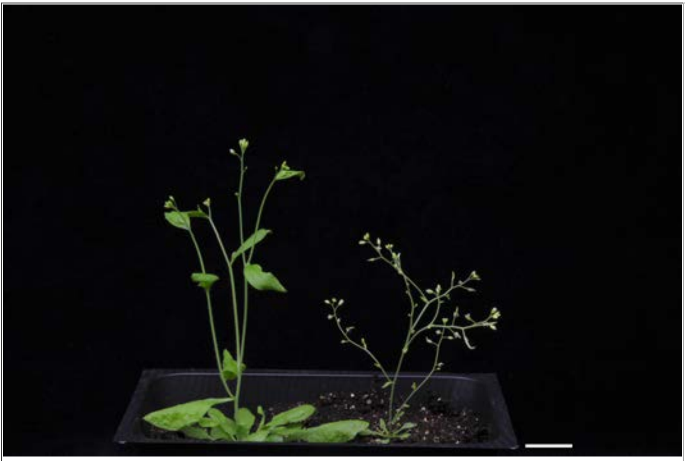

Ar50-14-pl1

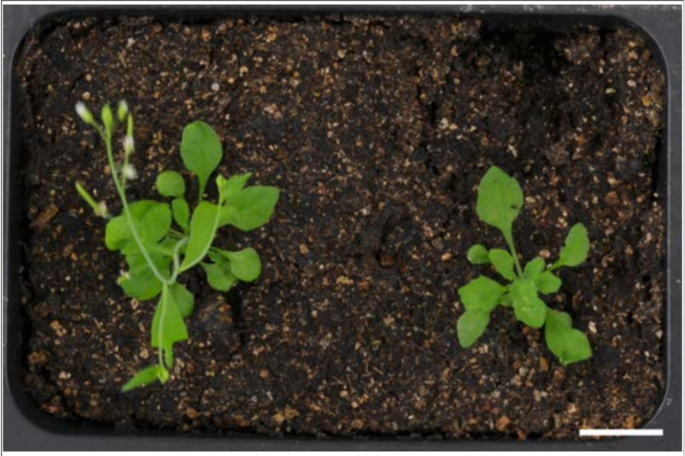
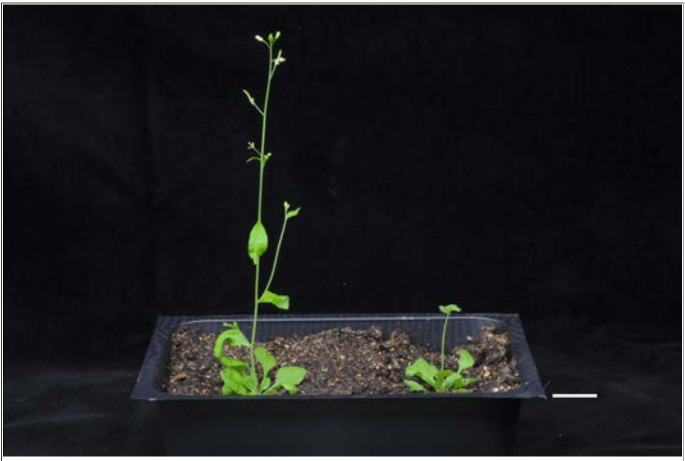

Ar50-17-N1

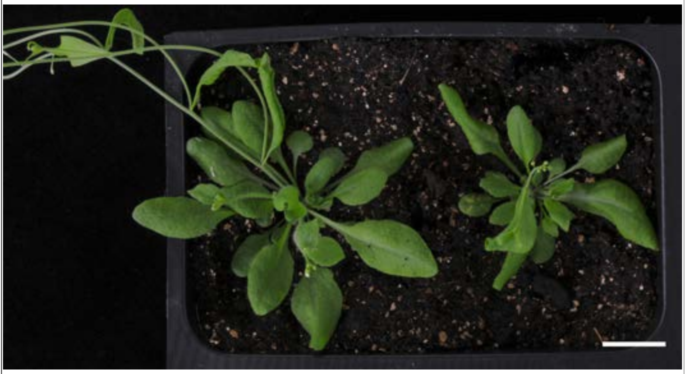

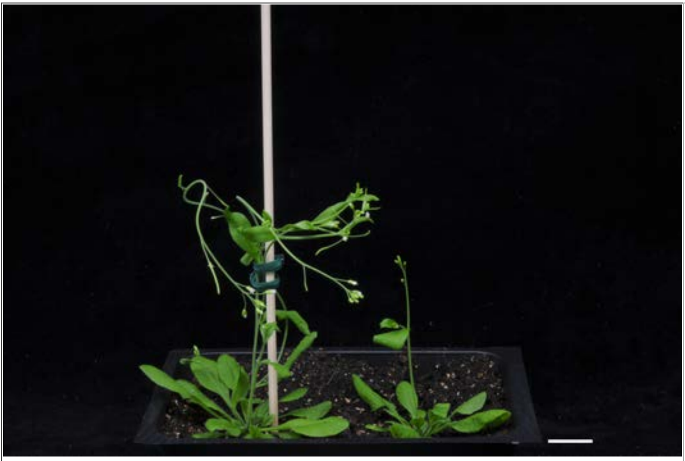

Ar50-21-as1

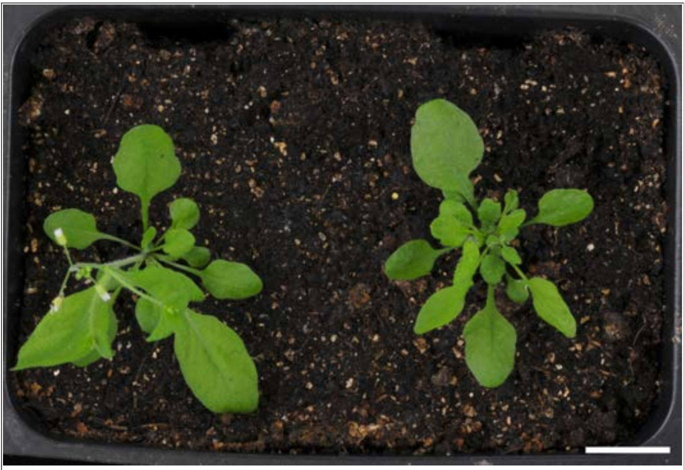

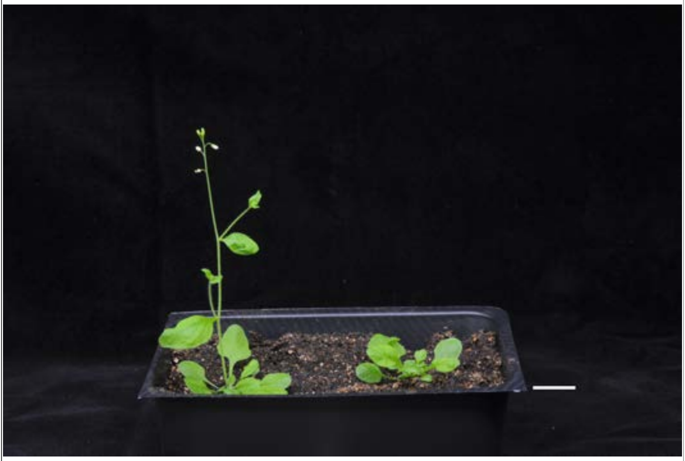

Ar50-25-N2

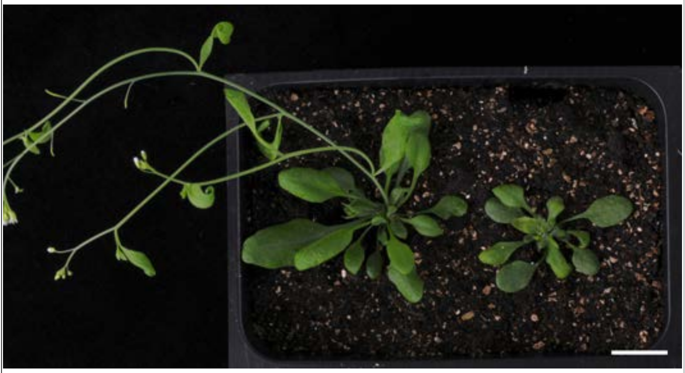

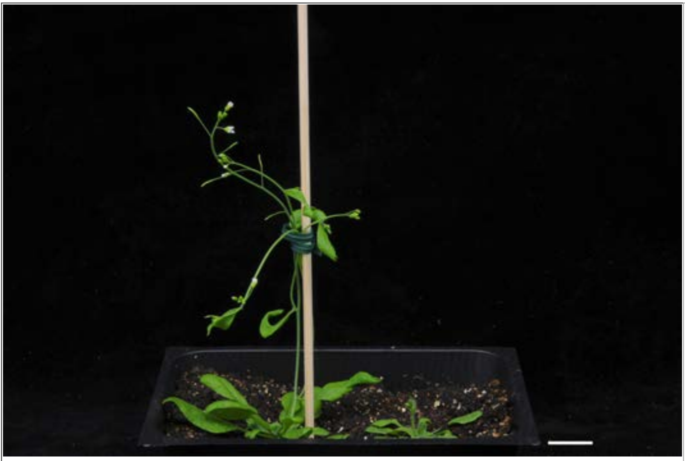

Ar50-27-as1

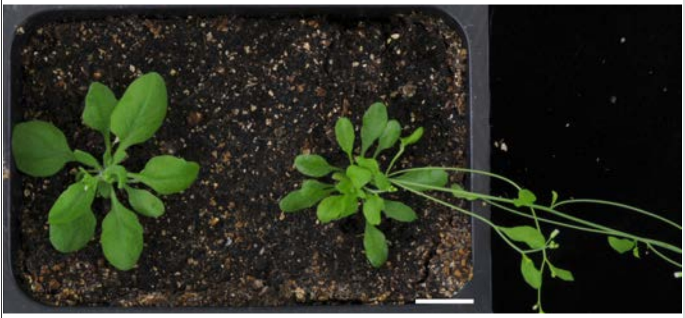
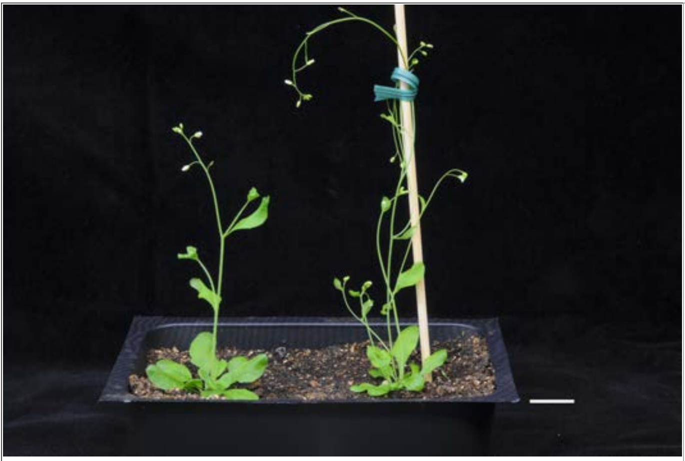

Ar50-33-pg1

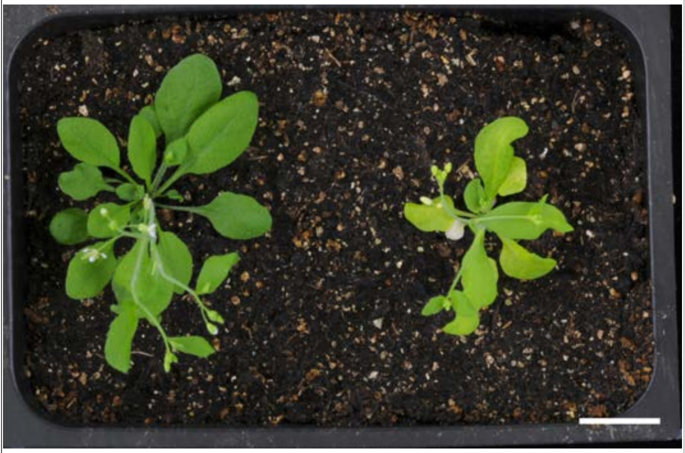
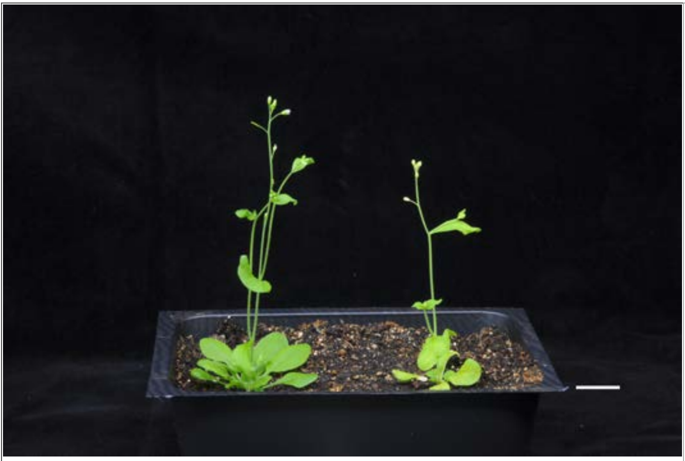

Ar50-44-as1

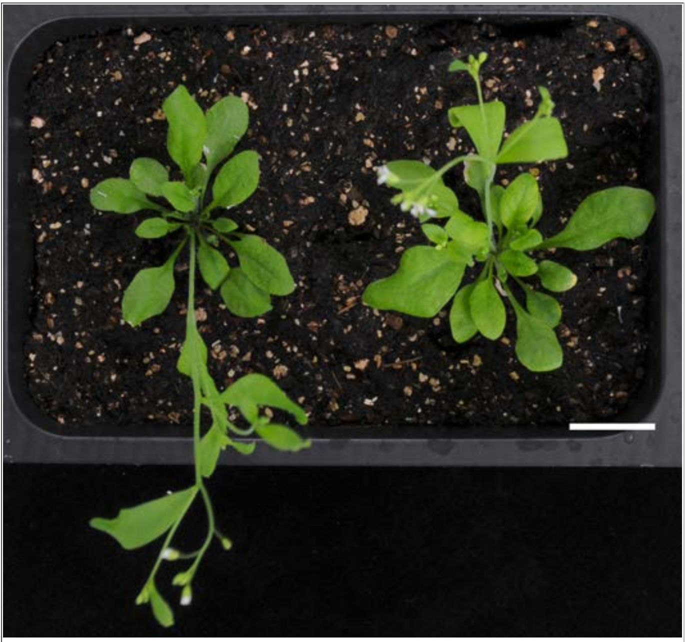
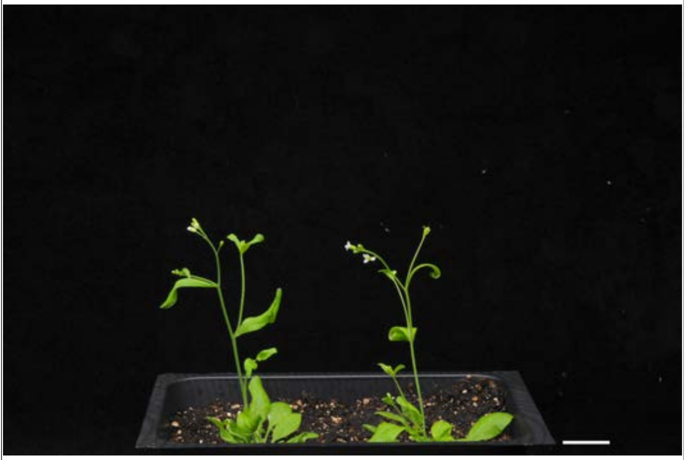

Ar50-46-pl1

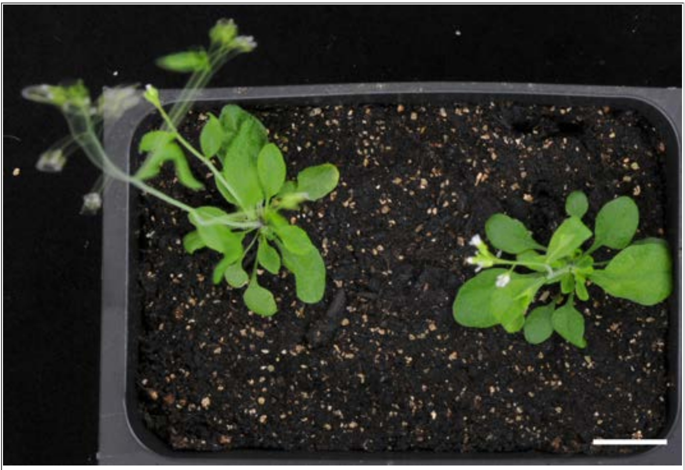

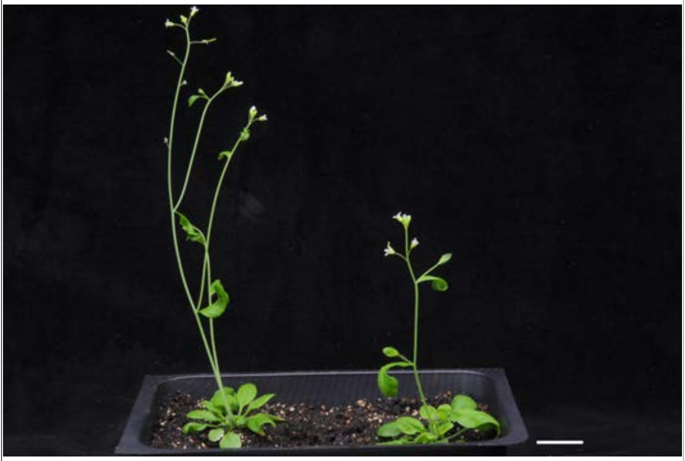

Ar50-52-nl1

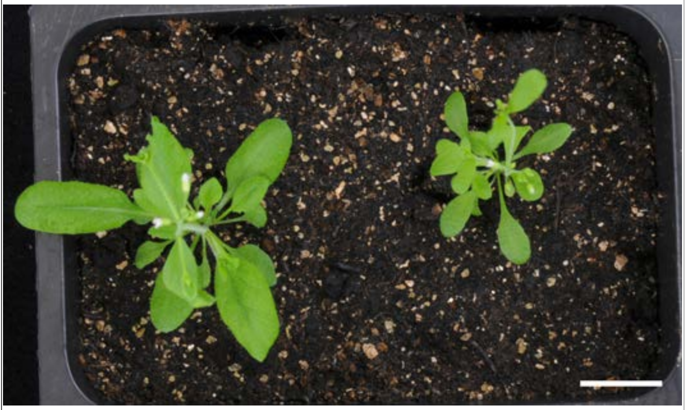

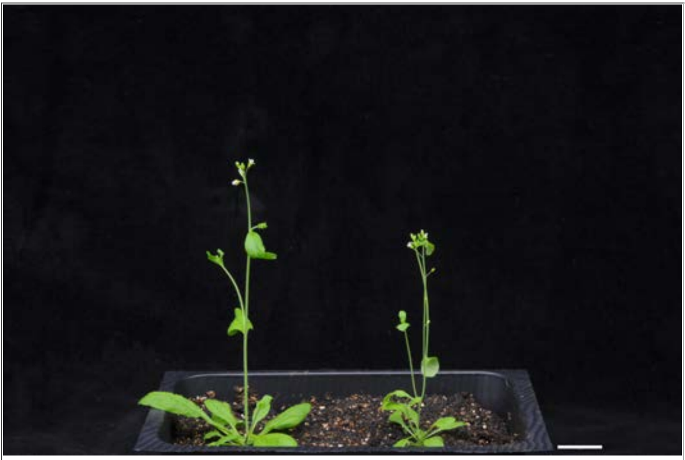

Ar50-55-as3

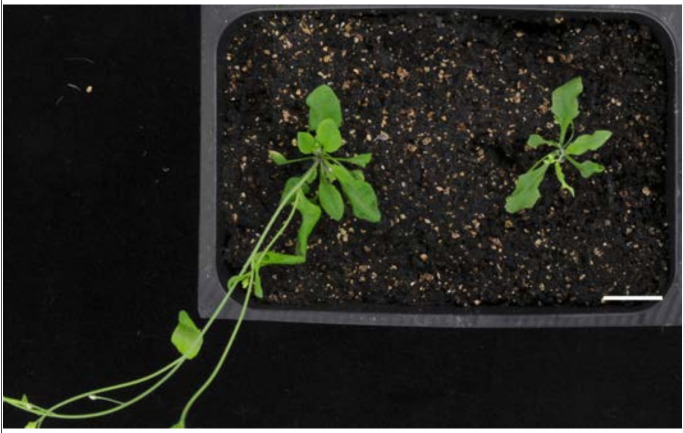

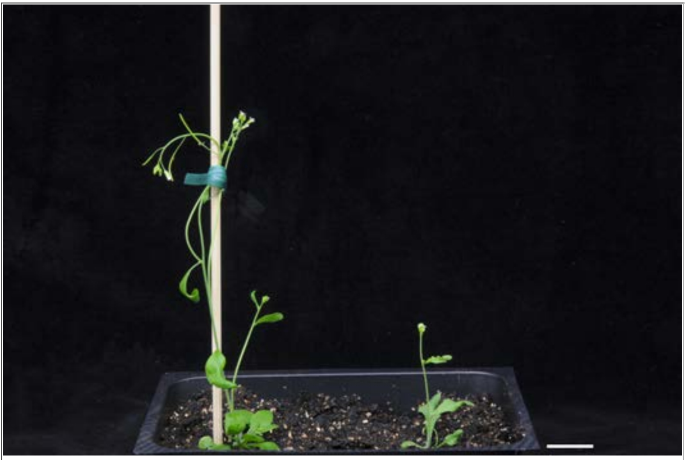

Ar50-58-nl1

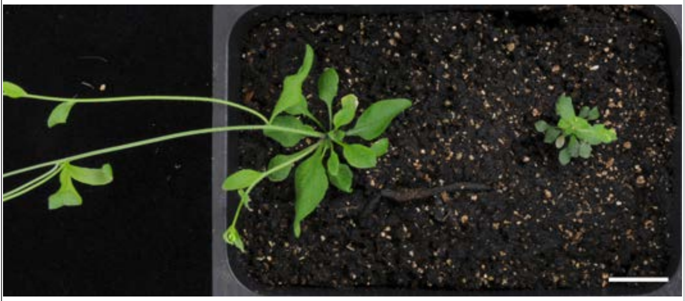

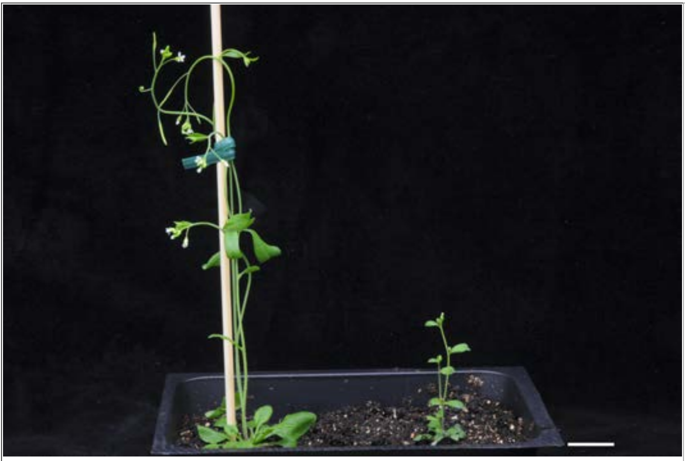

Ar50-60-as1

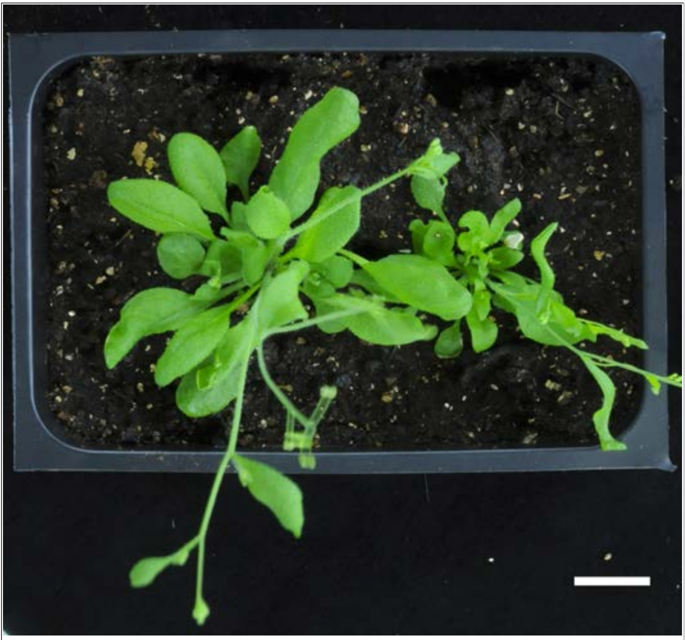

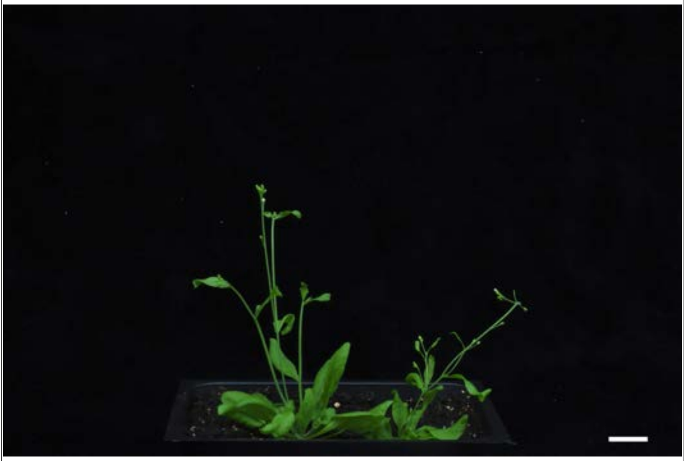

Ar50-71-N2

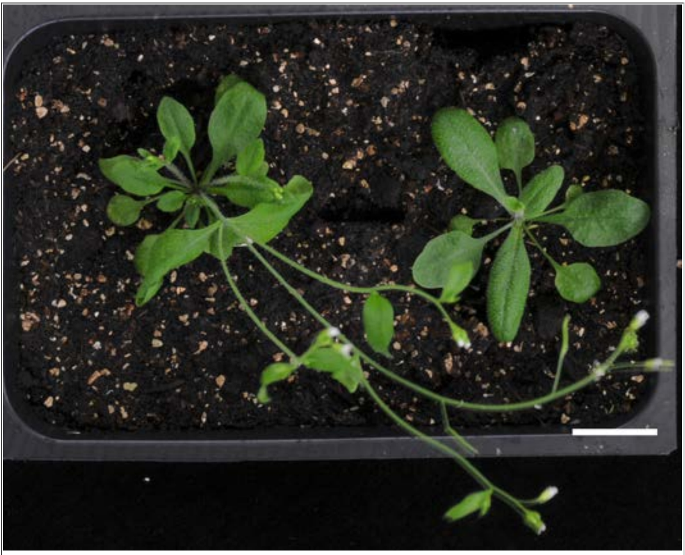

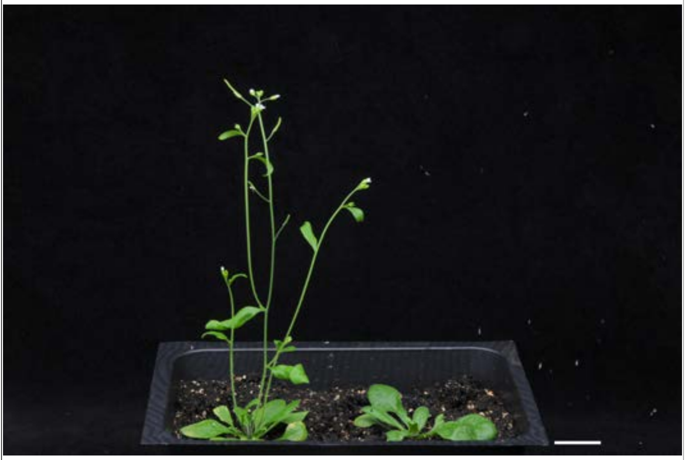

Ar50-72-N2

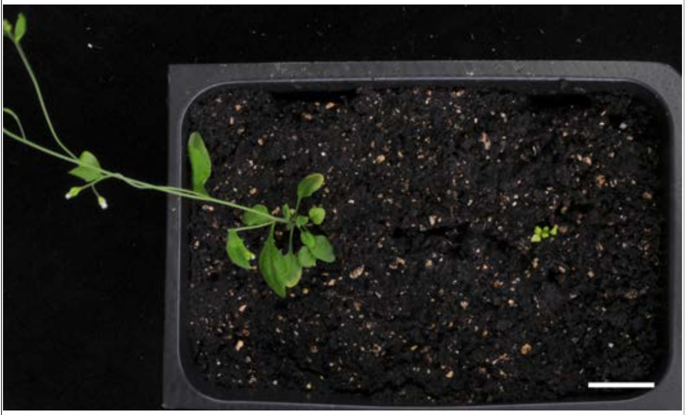

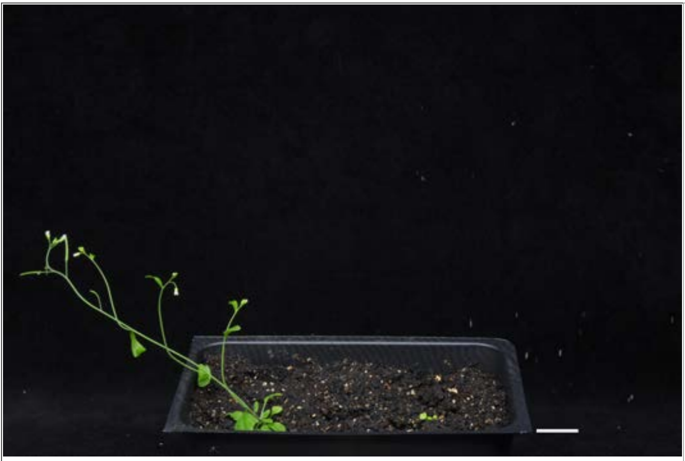

Ar50-74-N1

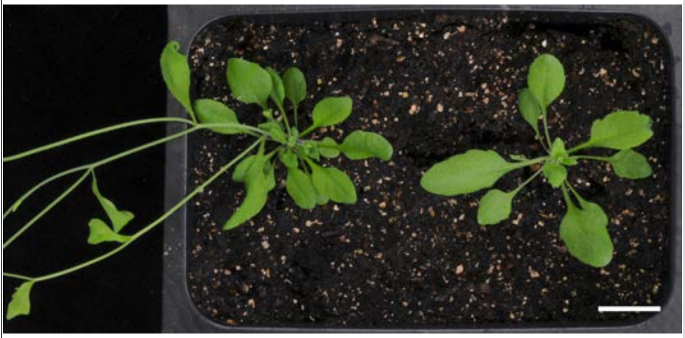

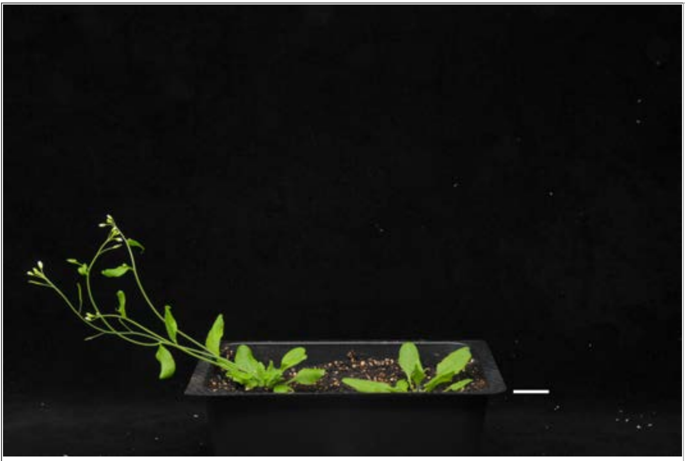

Ar50-74-pl5

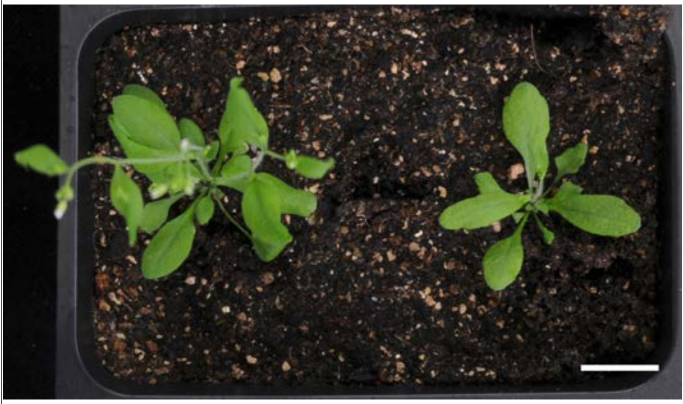

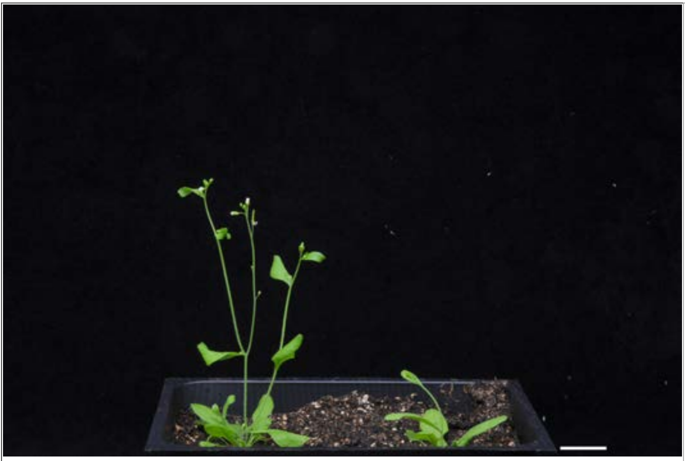

Ar50-81-N1

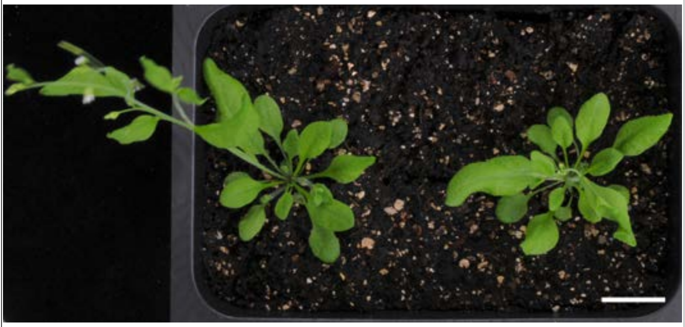

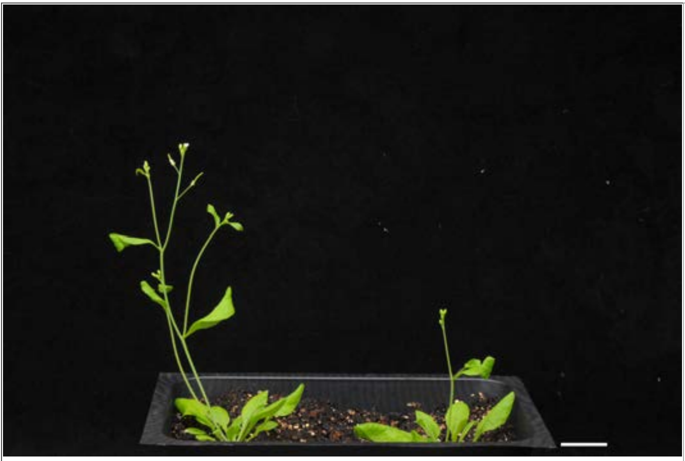

Ar50-81-pg1

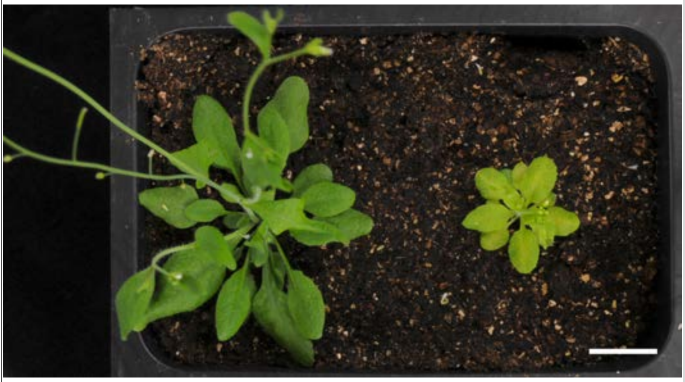

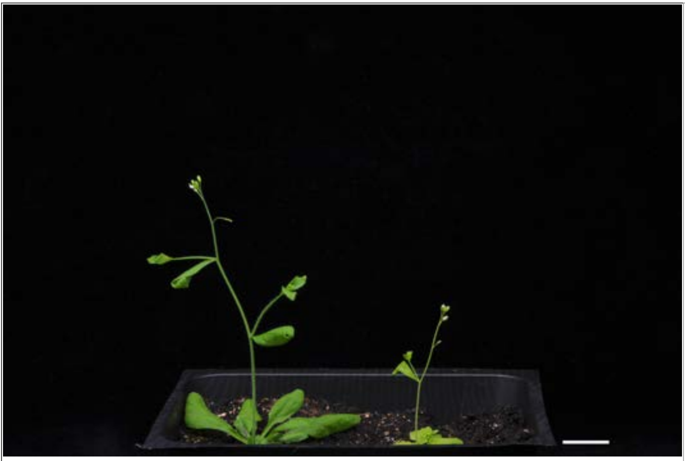

Ar50-83-N1

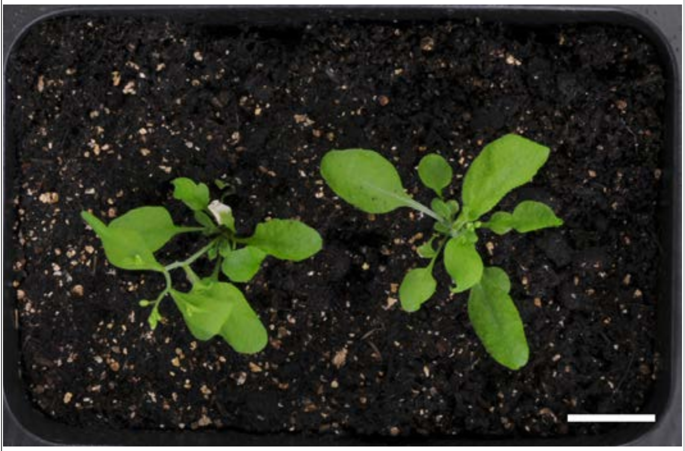

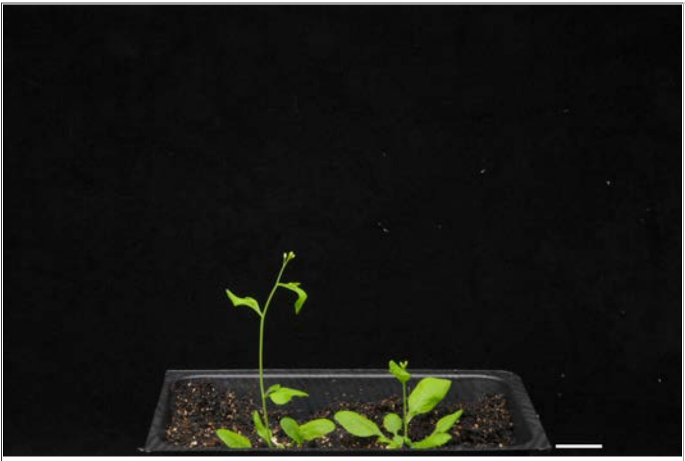

C100-5-pu1

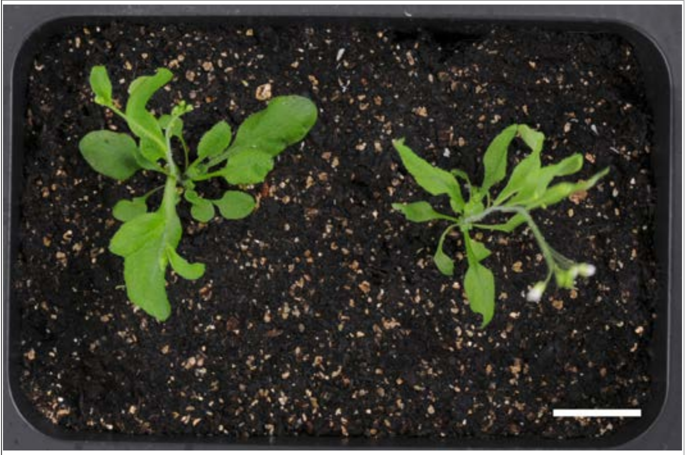

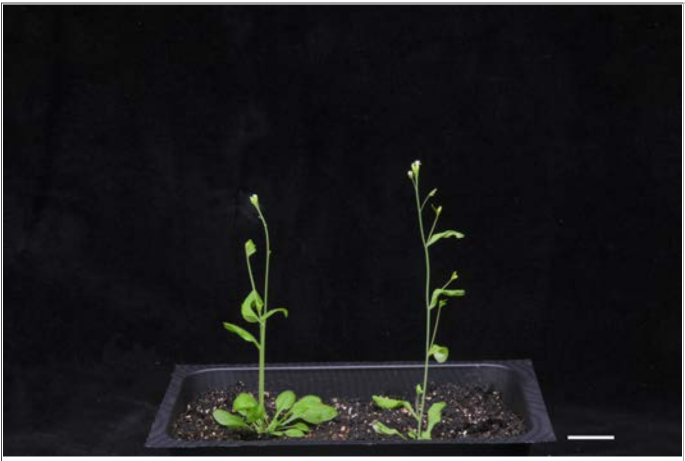

C100-9-pg1

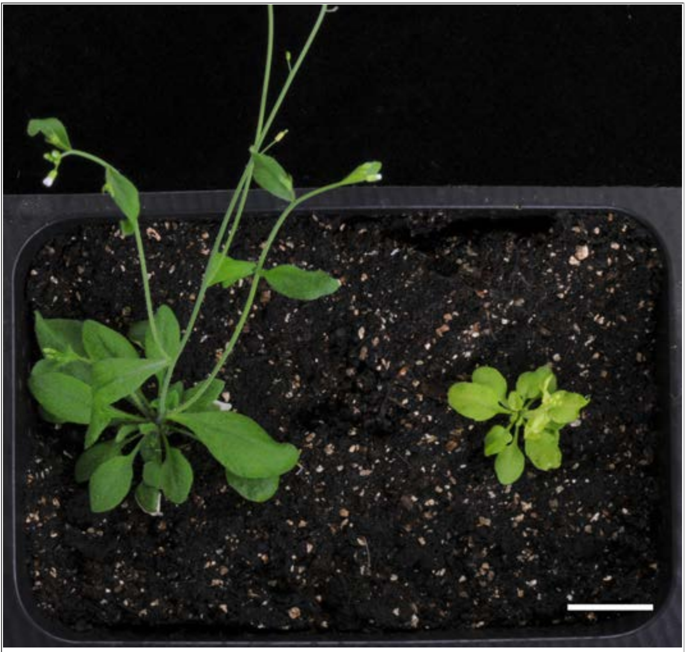

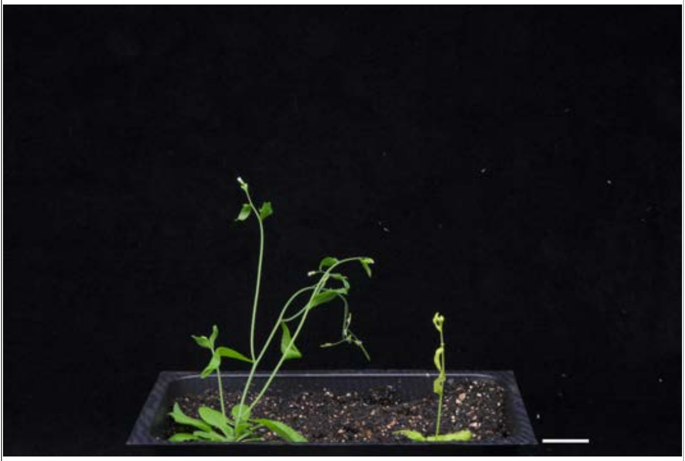

C100-13-pg1

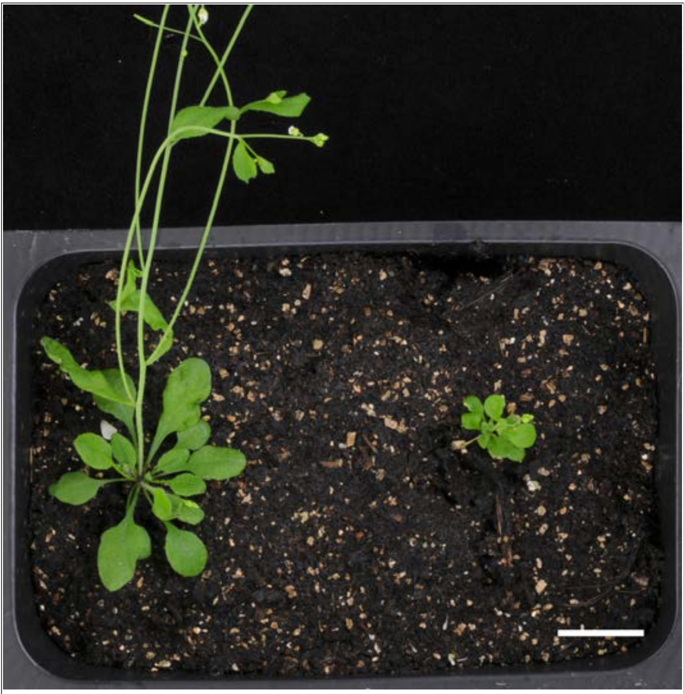

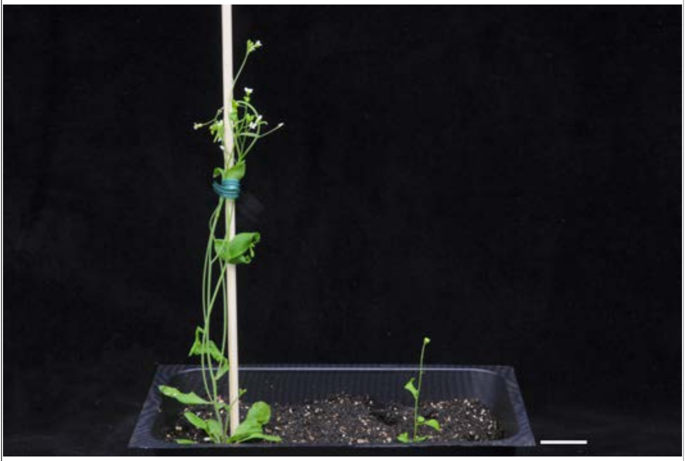

C100-16-as1

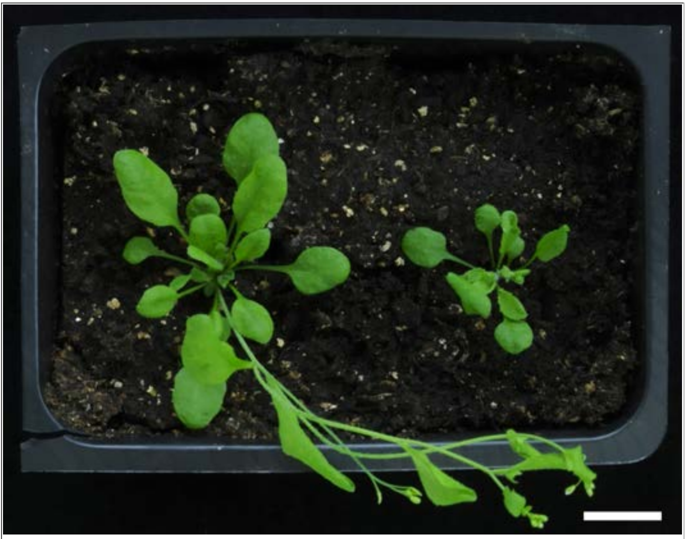

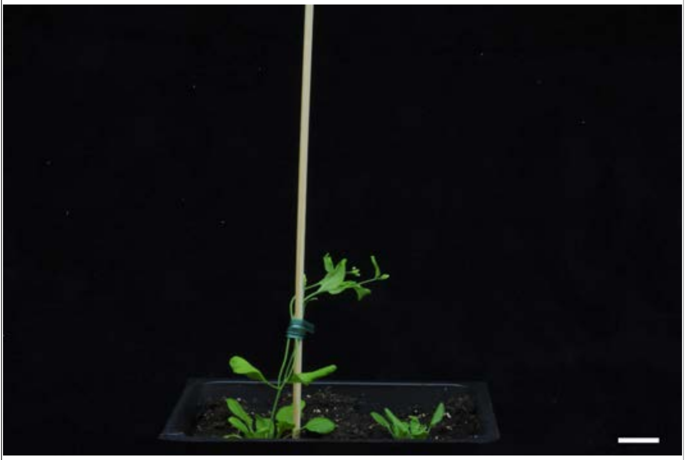

C100-17-late1

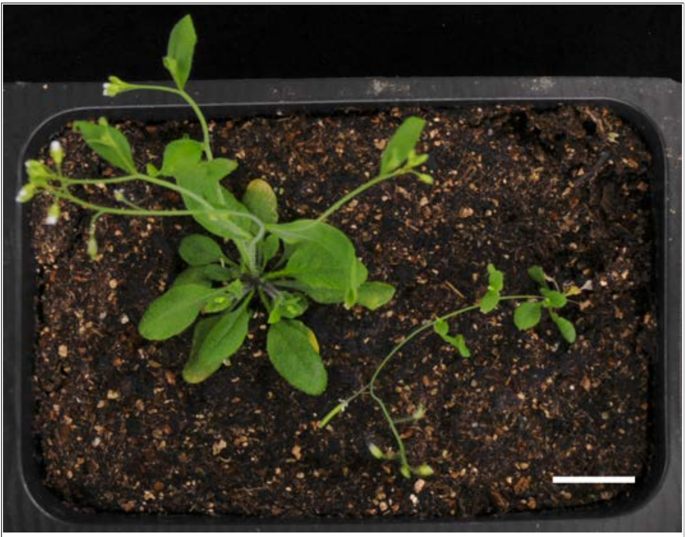

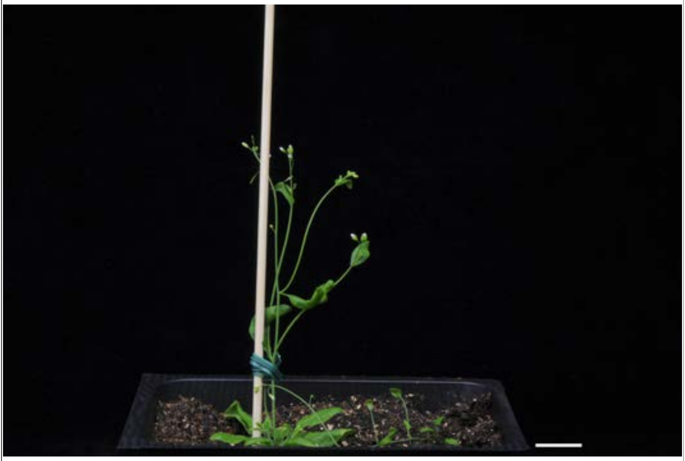

C100-23-N2

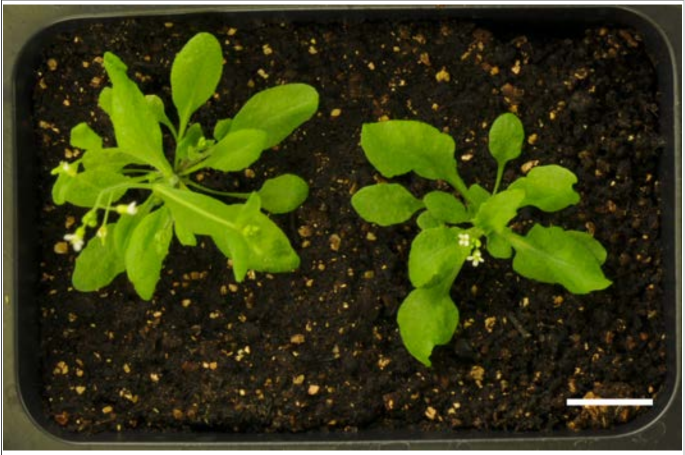

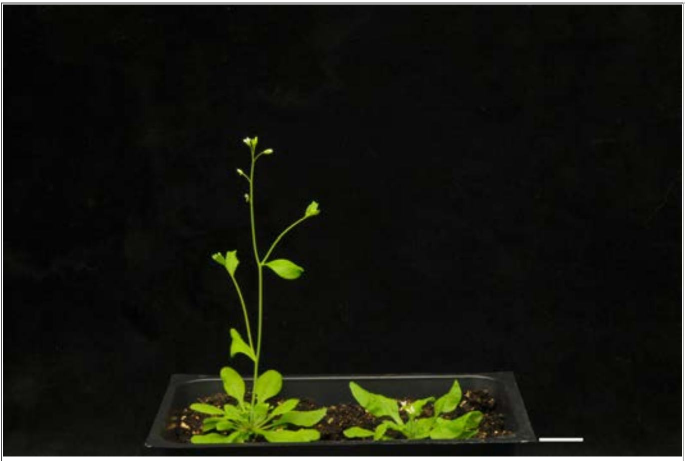

C100-25-as1

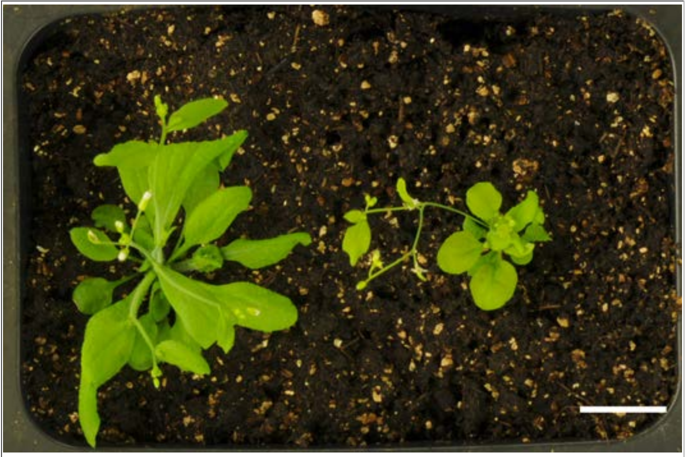

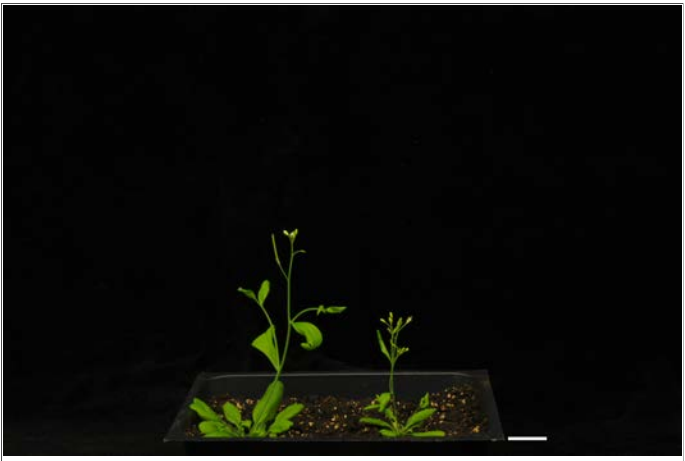

C100-27-c3-1	
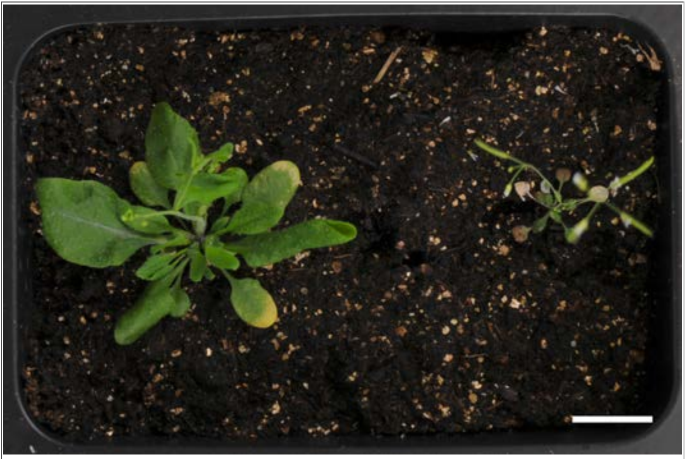	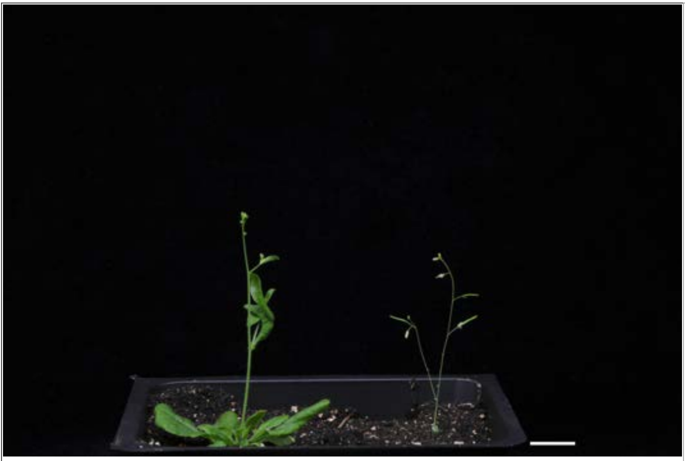

C100-30-late1	
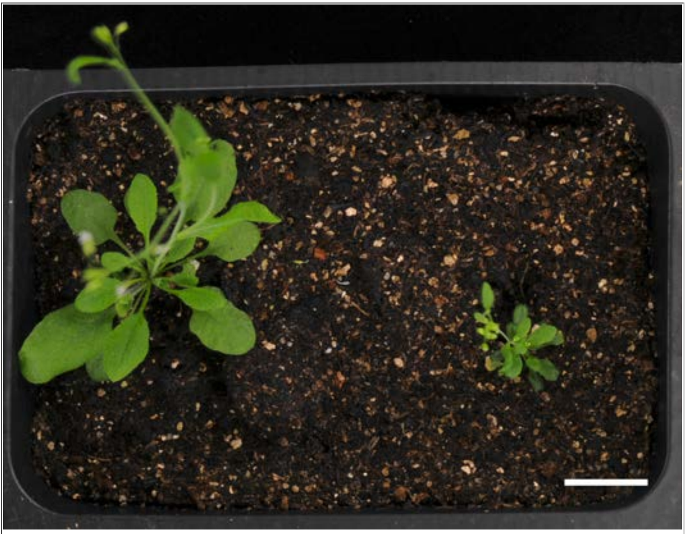	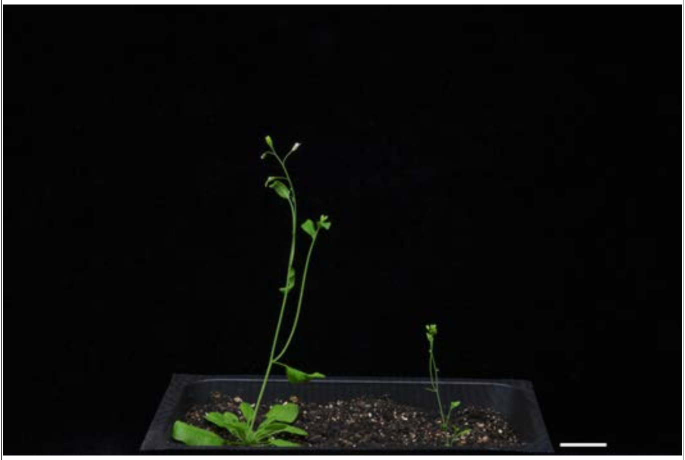

C100-34-as1	
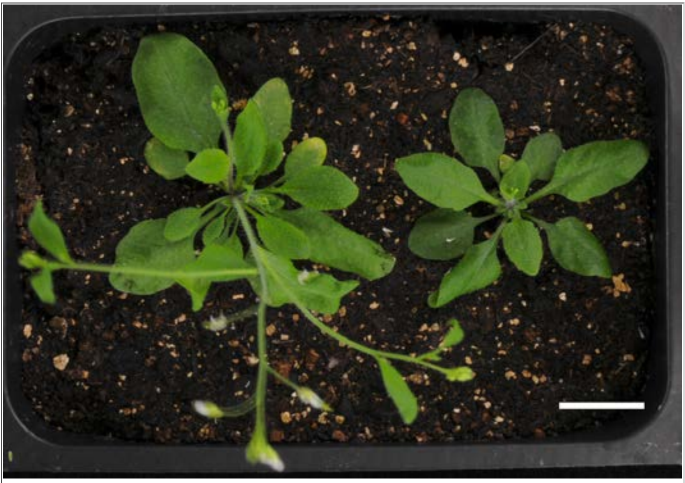	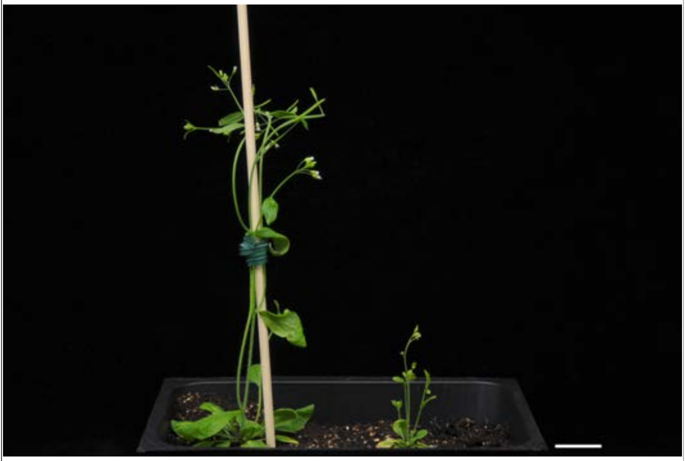

C100-37-N2

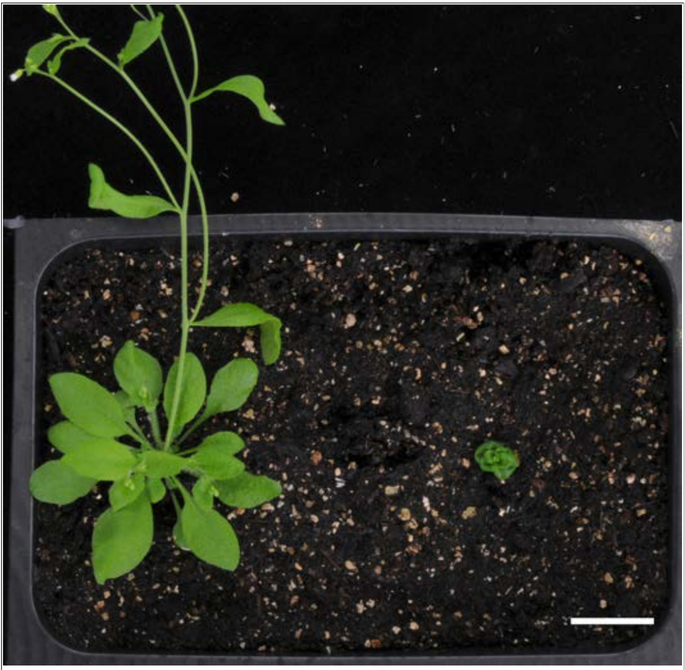

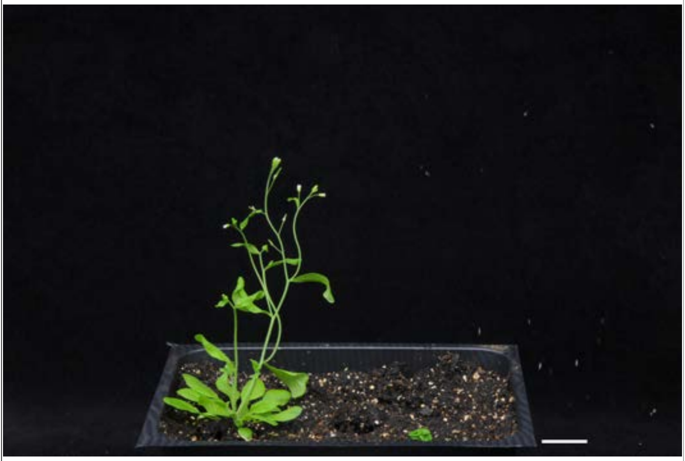

C100-61-pl1

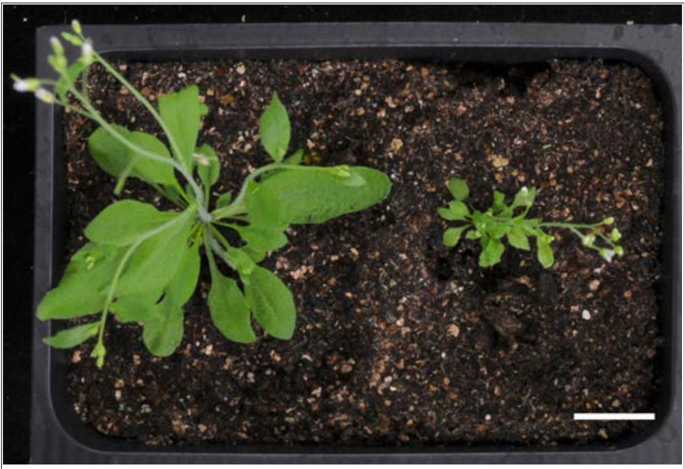

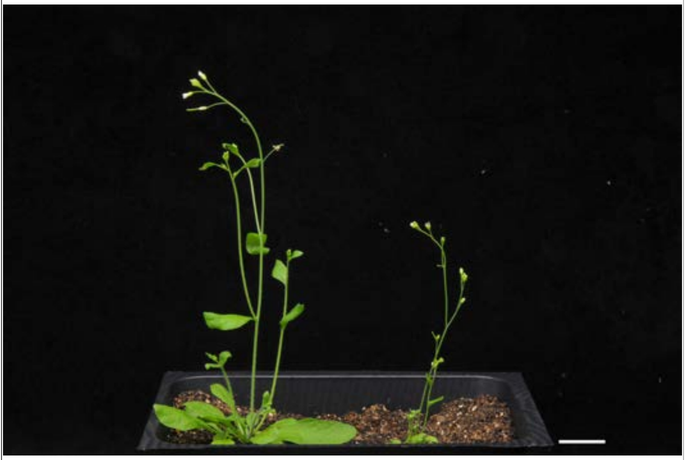

C100-70-N2

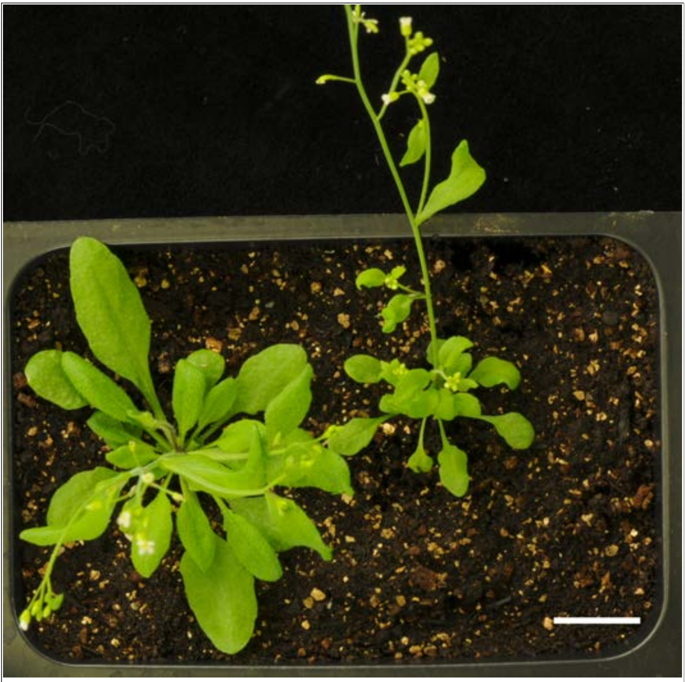

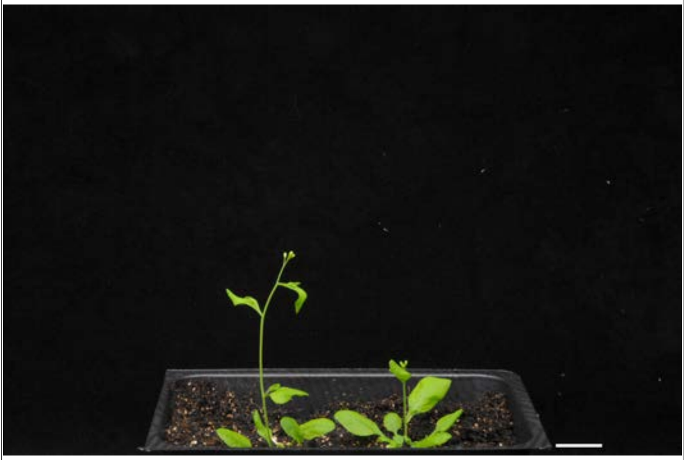

C100-71-N1

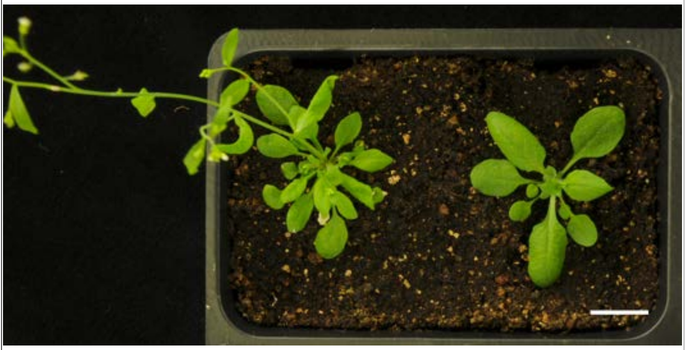

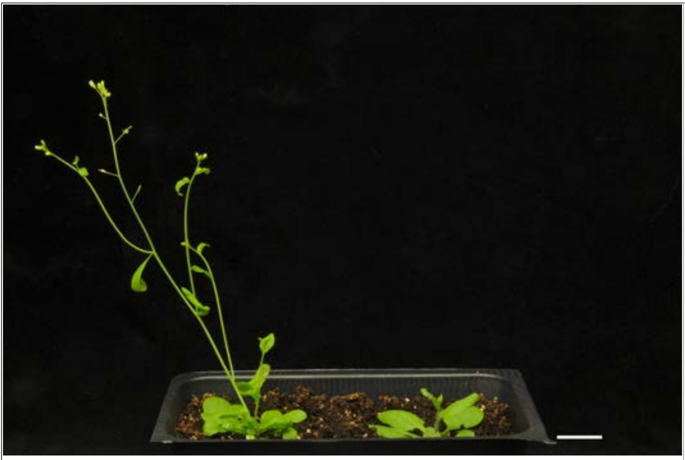

C100-72-pg2

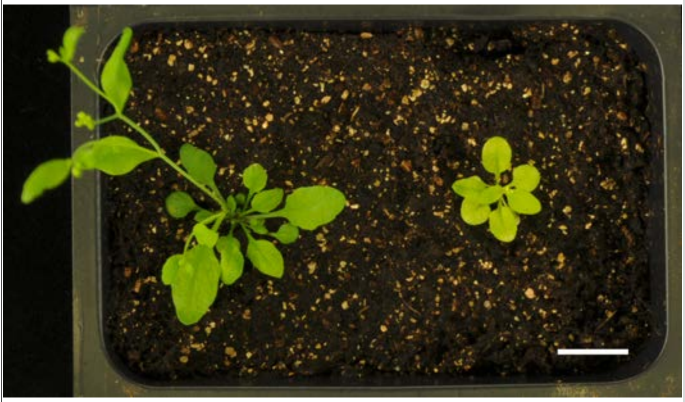

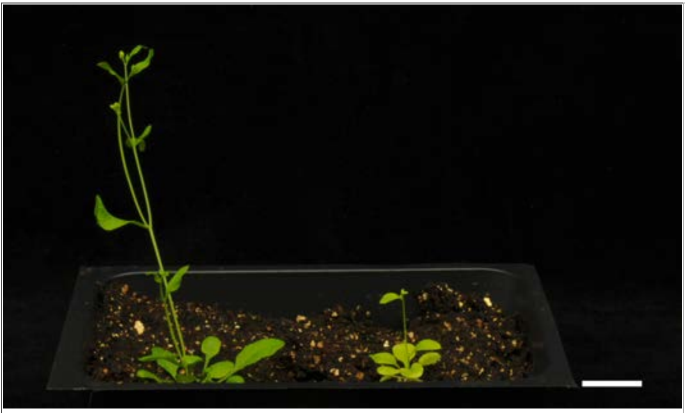

C100-96-late1

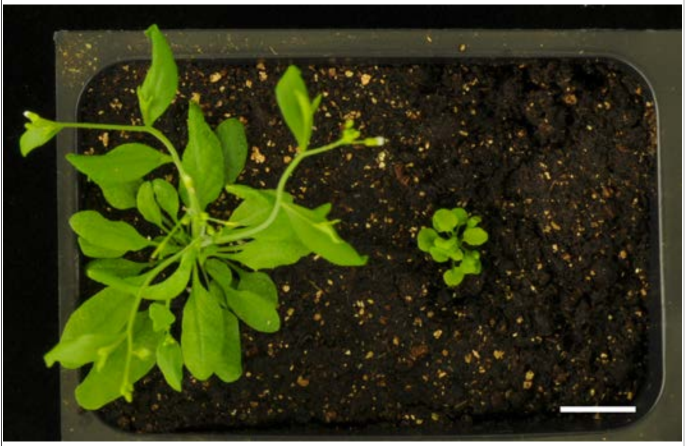

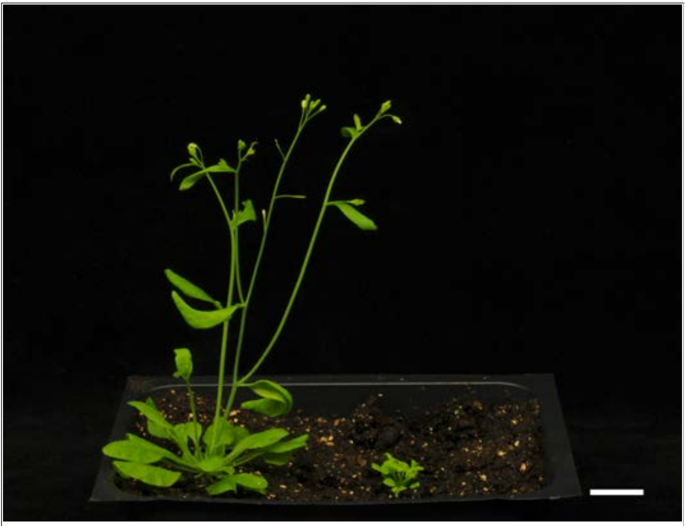

C200-17-N1

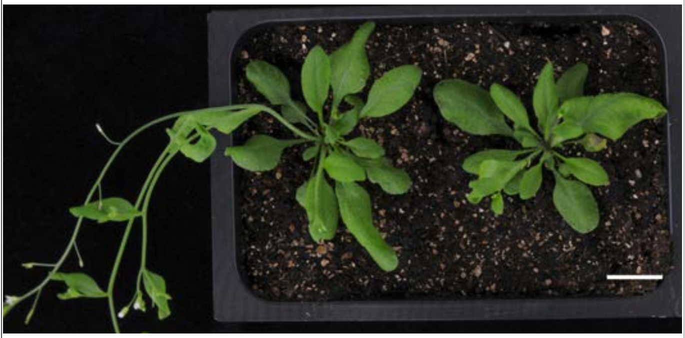

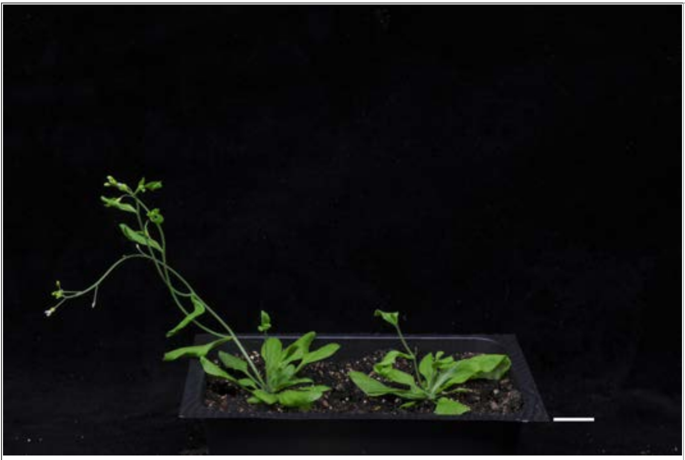

C200-19-as1

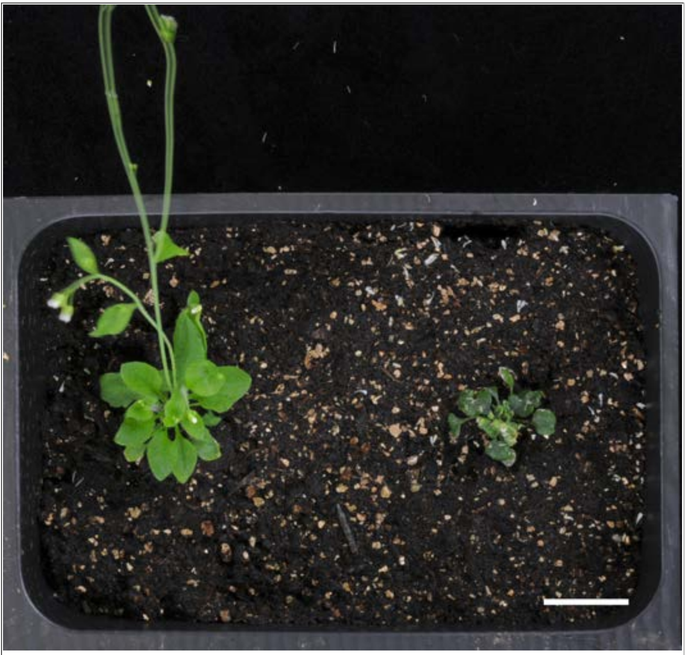

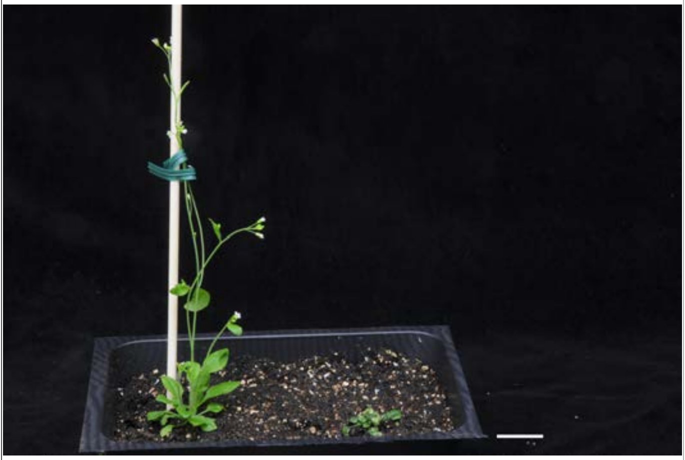

C200-23-as1

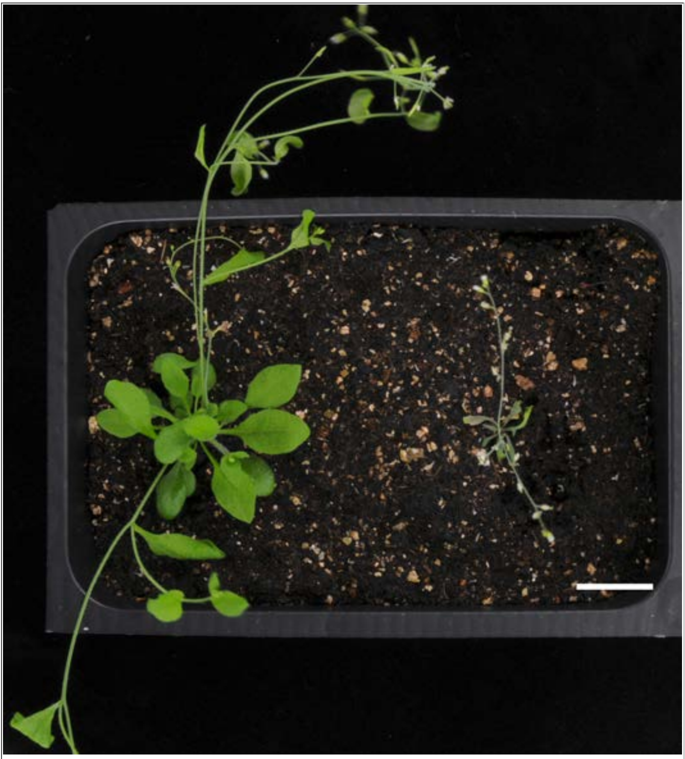

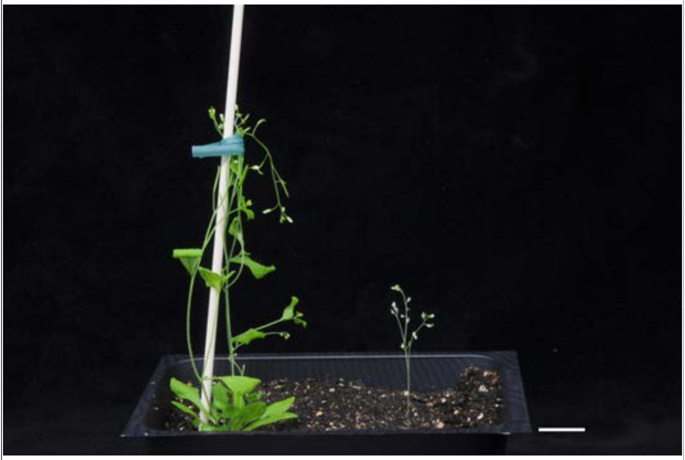

C200-27-as3

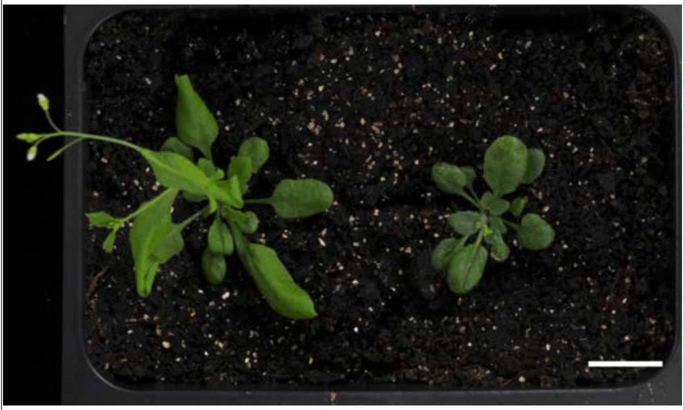

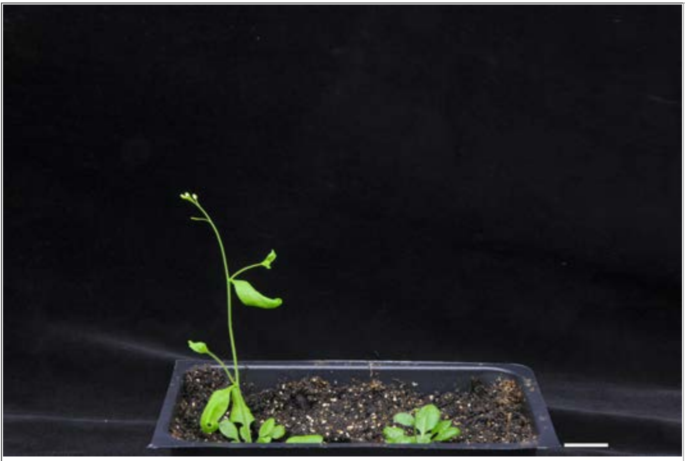

C200-37-as1

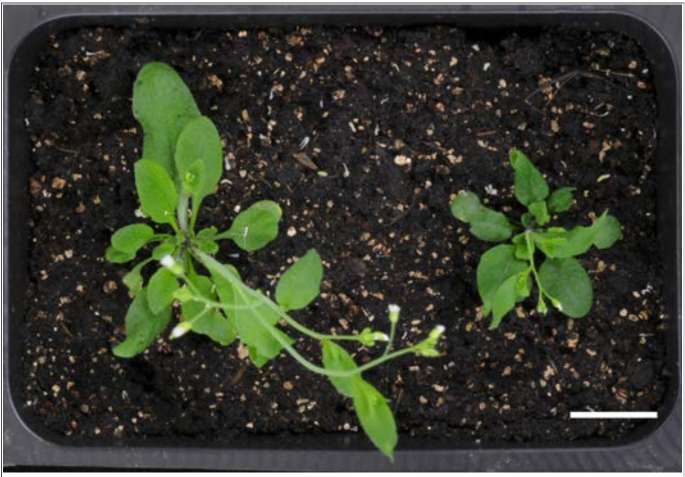	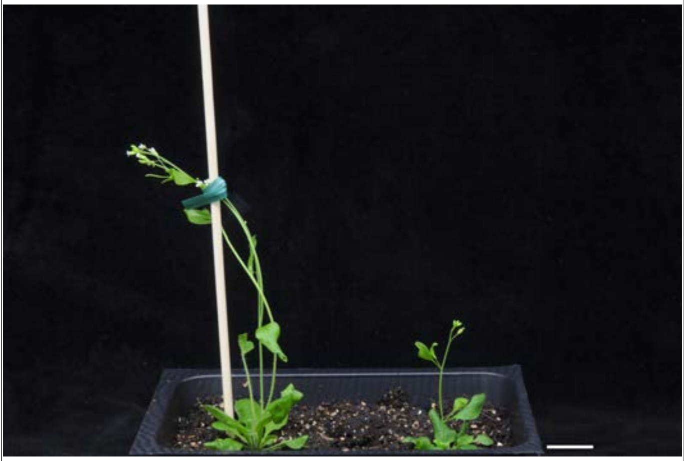

C200-38-N1

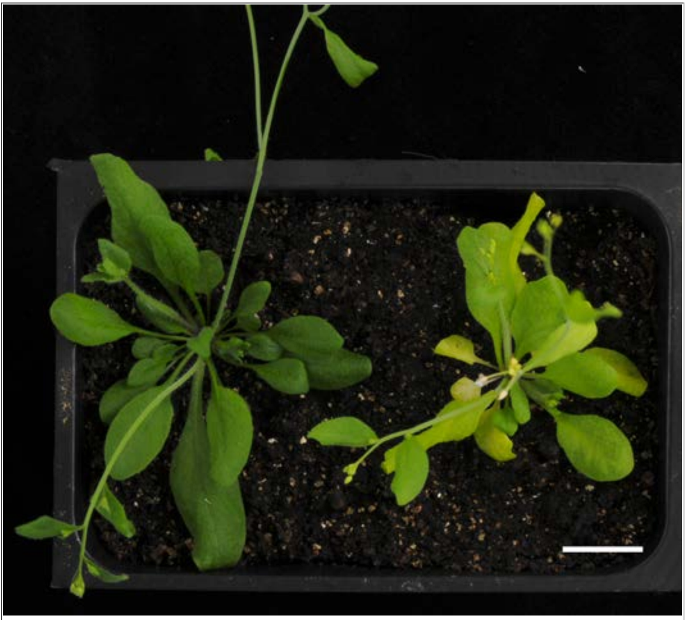	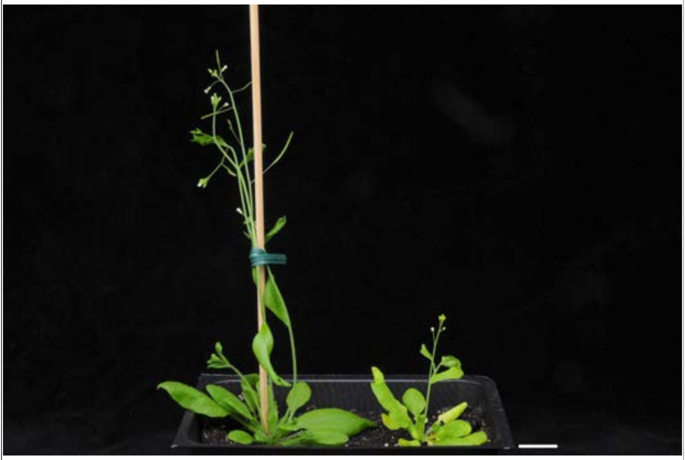

C200-38-pg1	
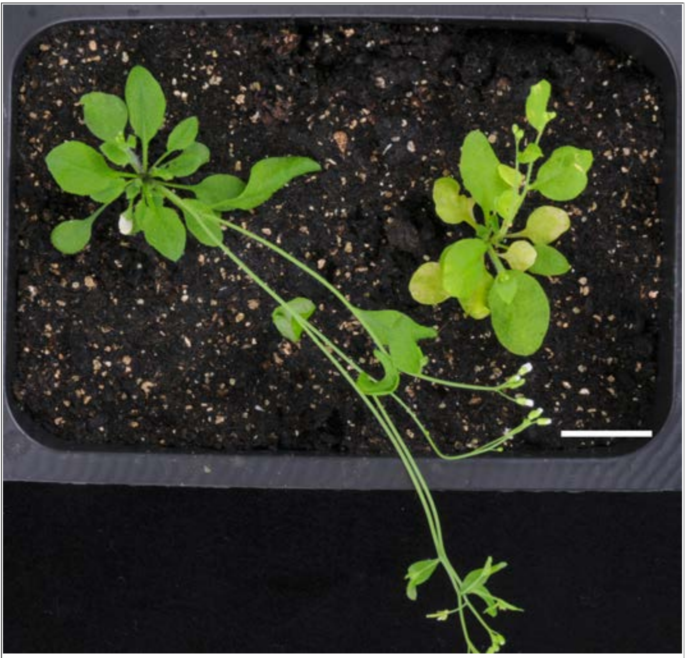	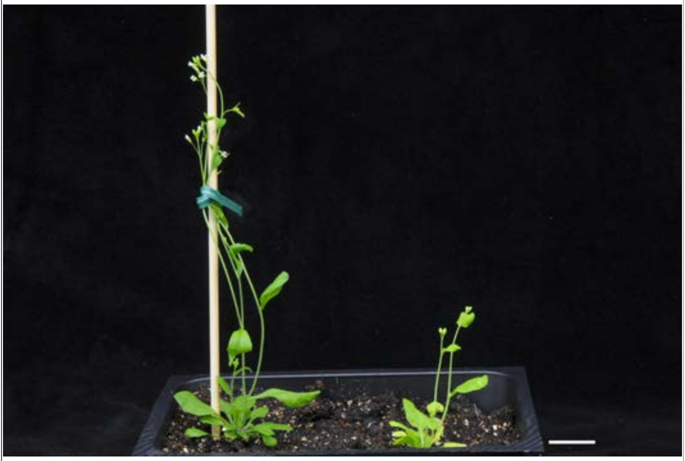

C200-41-pg2	
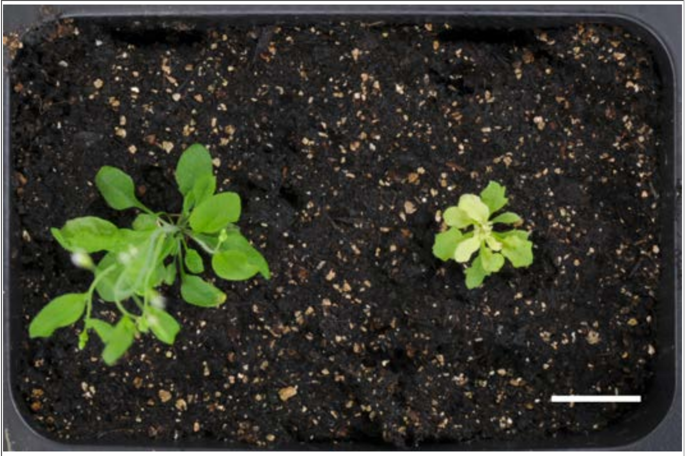	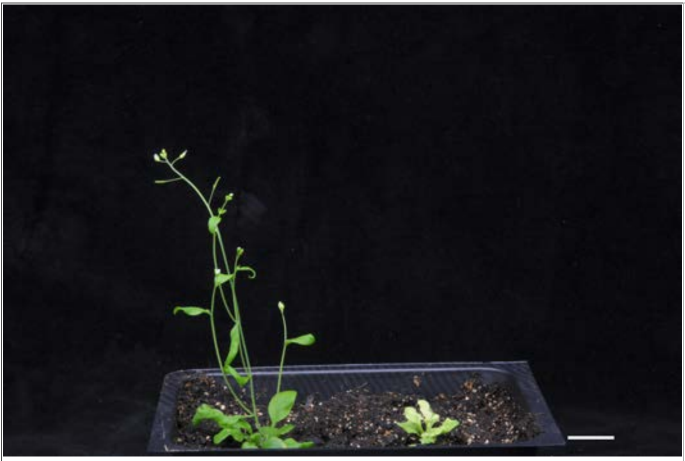

C200-43-pl1

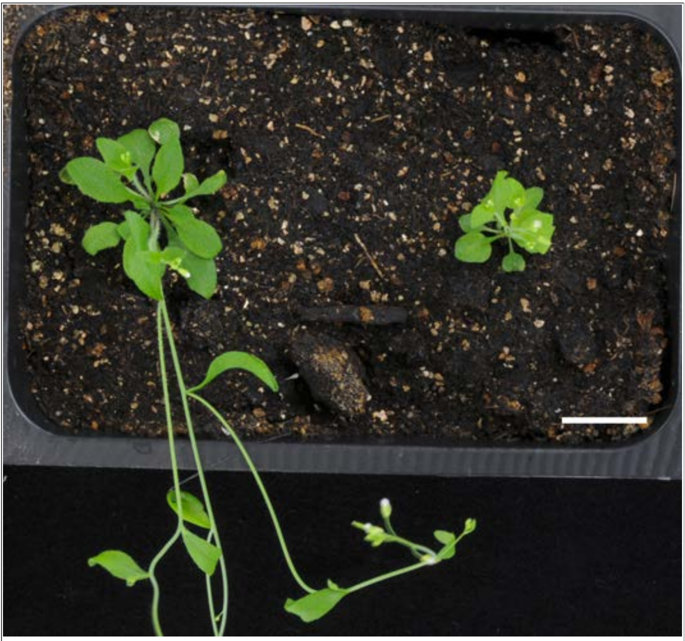

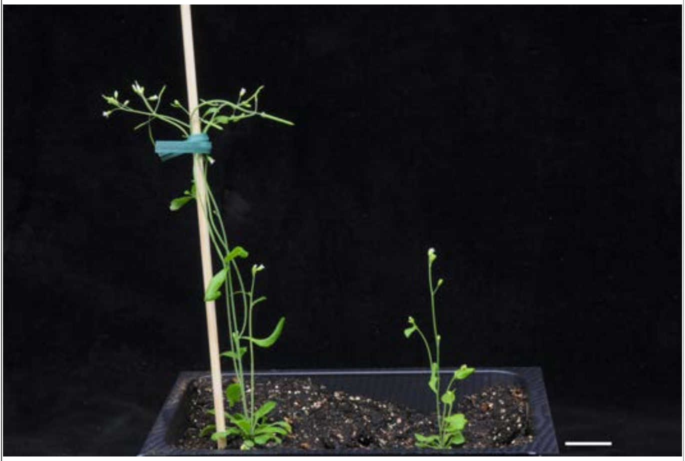

C200-46-N1

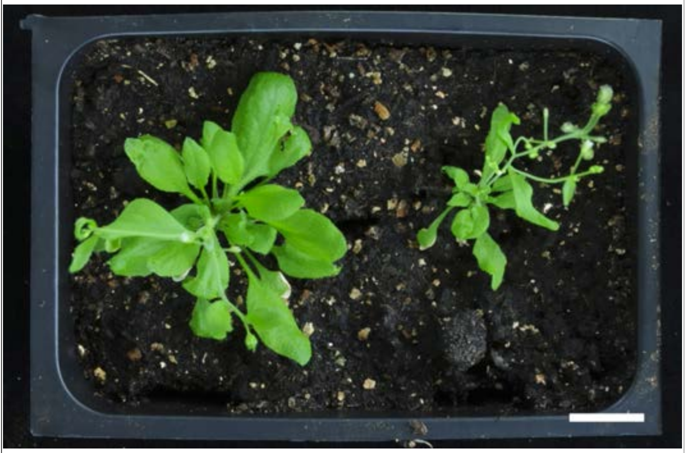

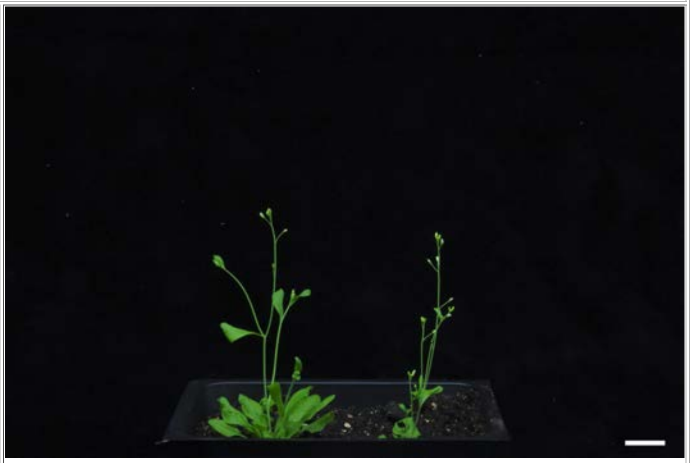

C200-46-N2

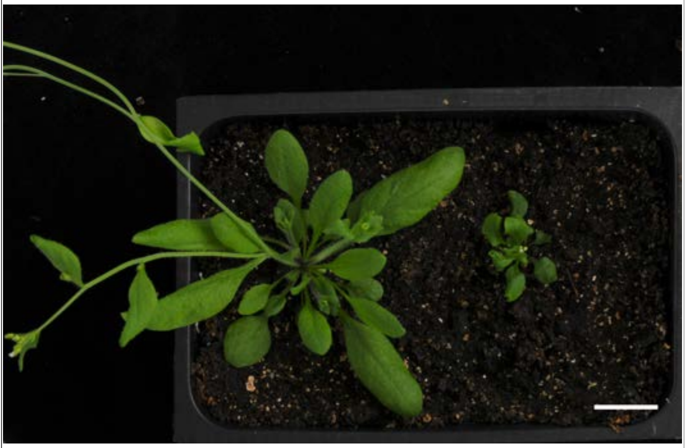

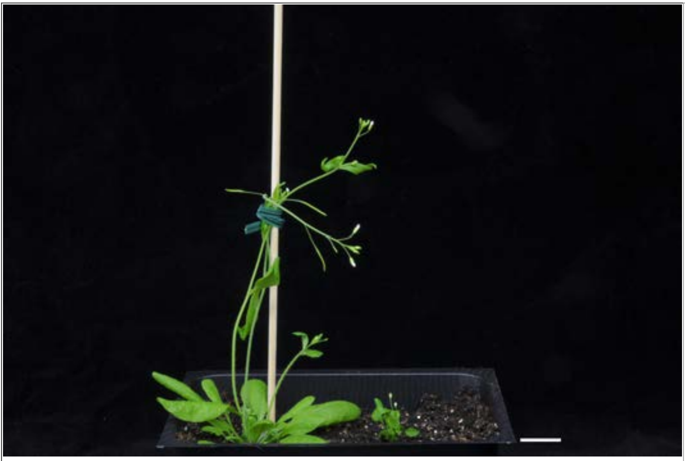

C200-47-N1

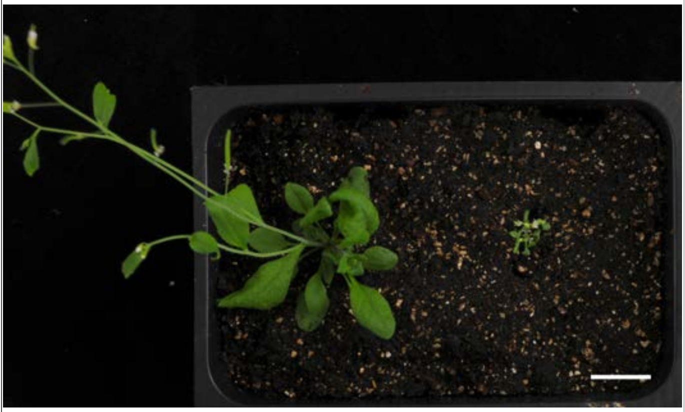

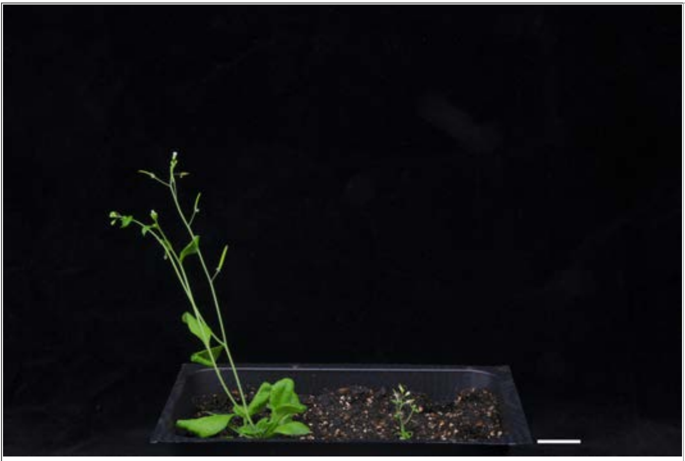

C200-47-pg1

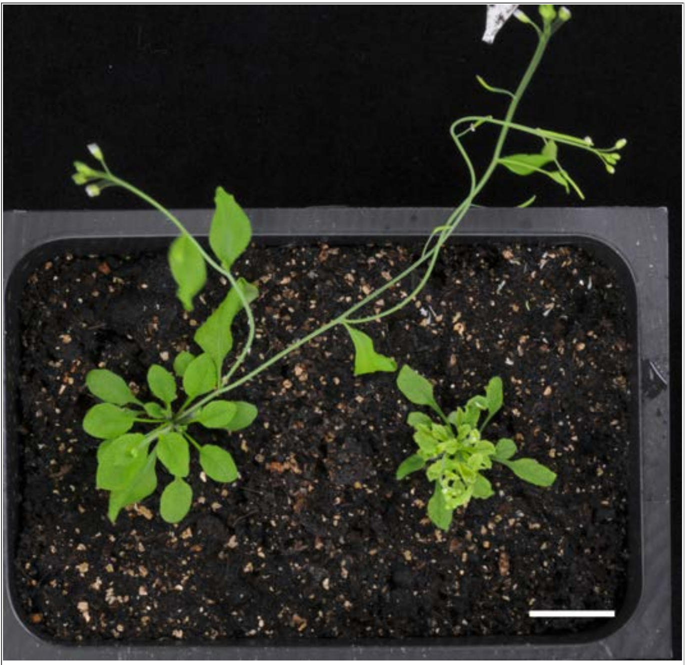

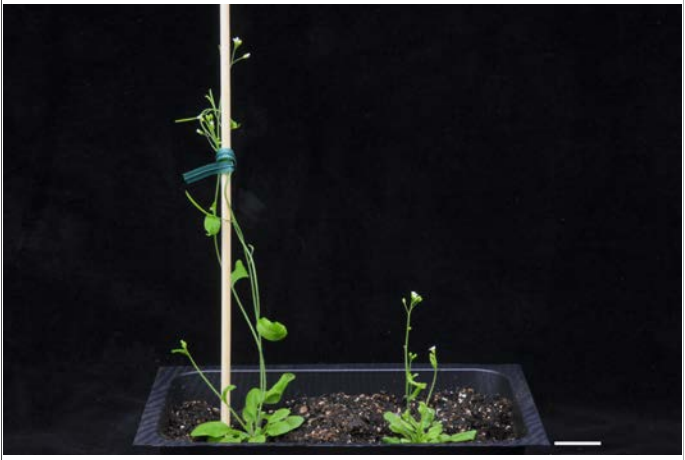

C200-48-late1

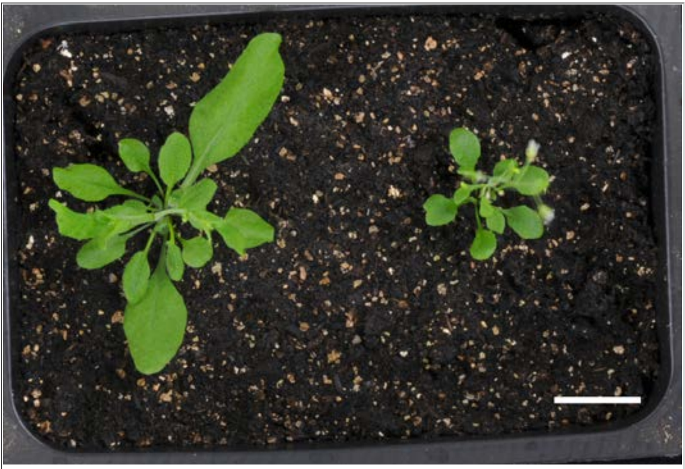

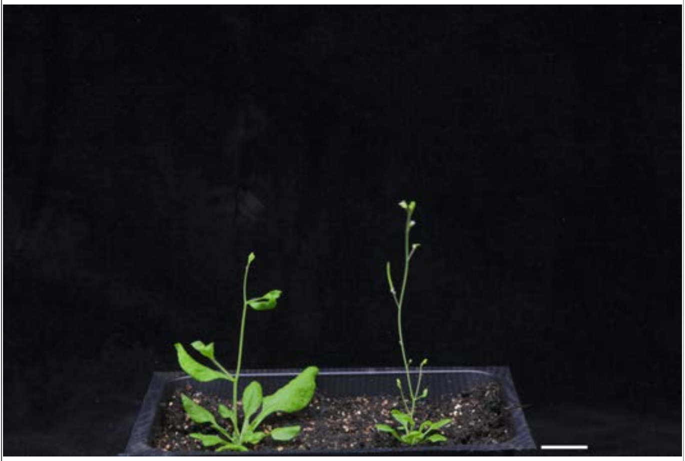

C200-49-pg2

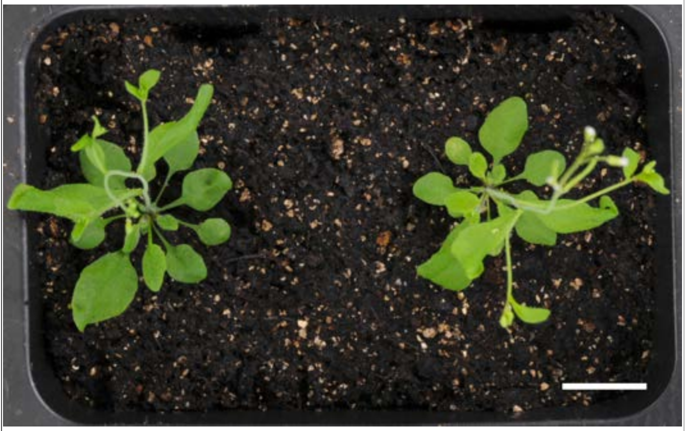

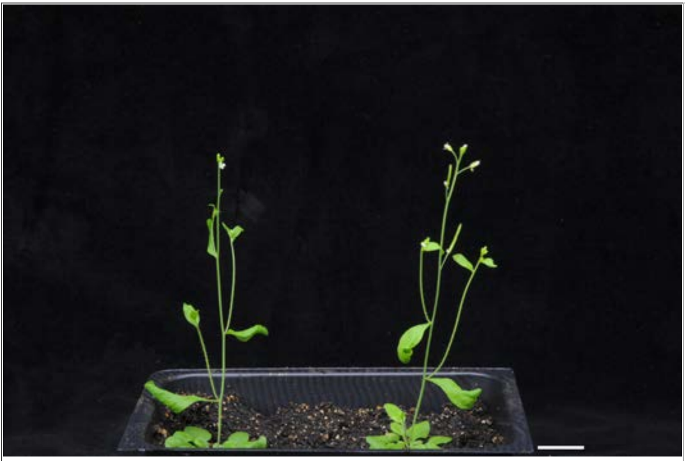

C200-51-as3

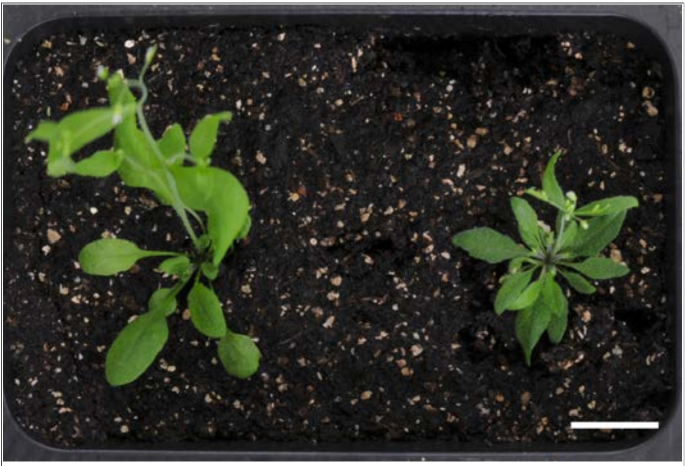

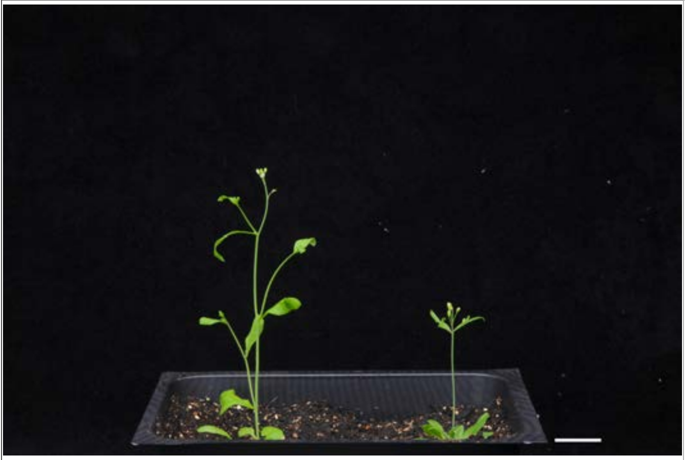

C200-56-as4

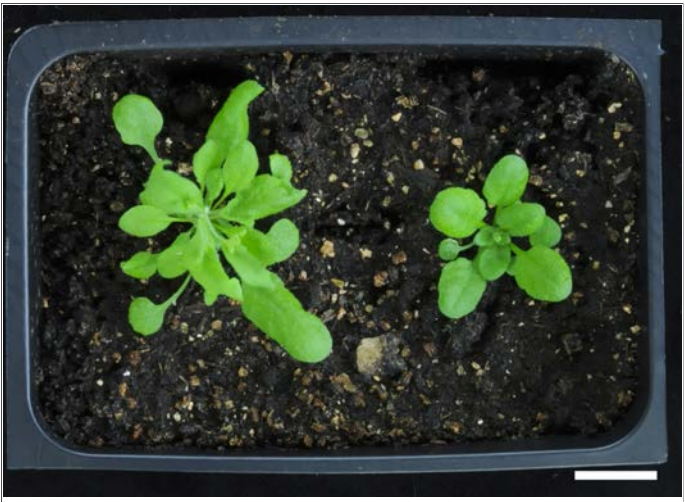

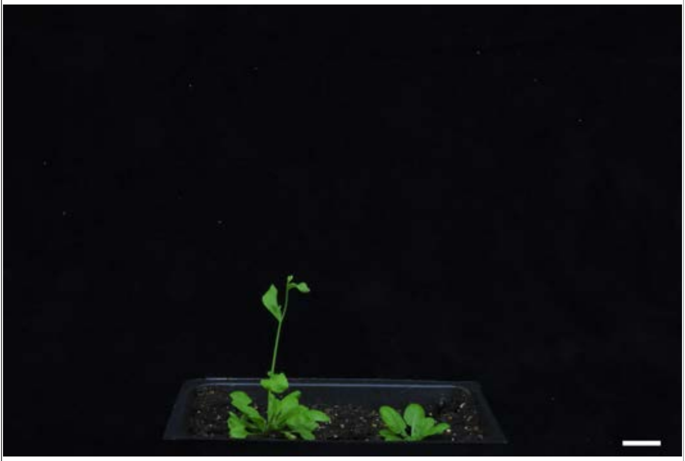

C200-60-as1

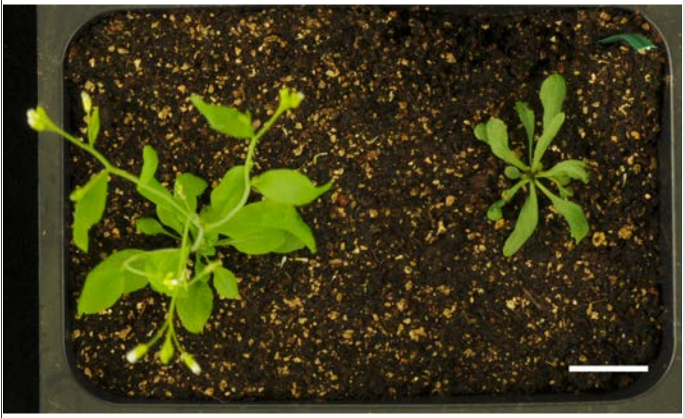
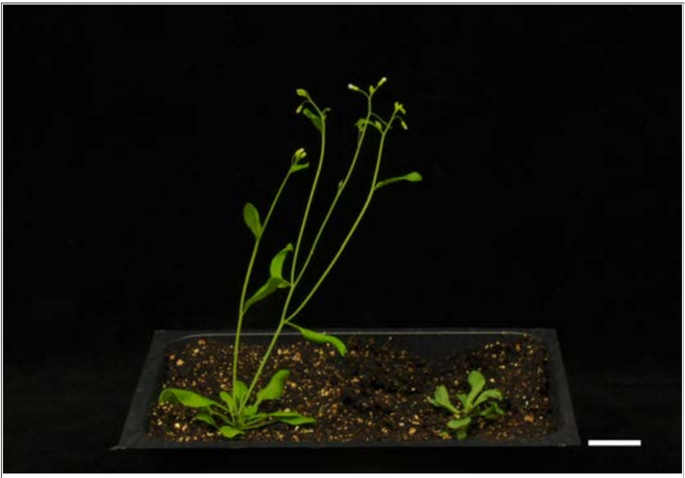

C200-63-N2

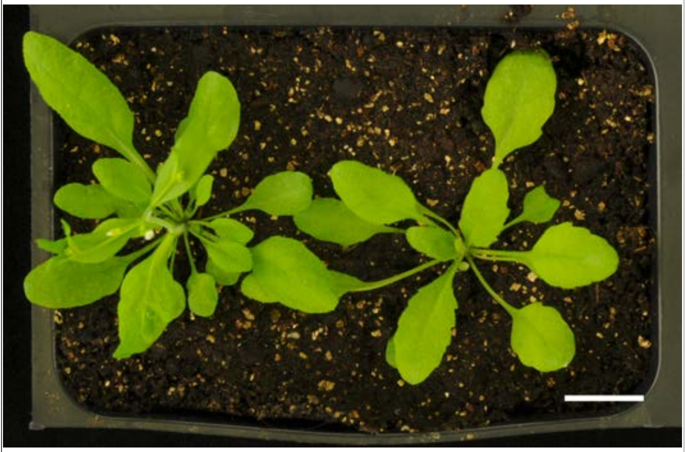
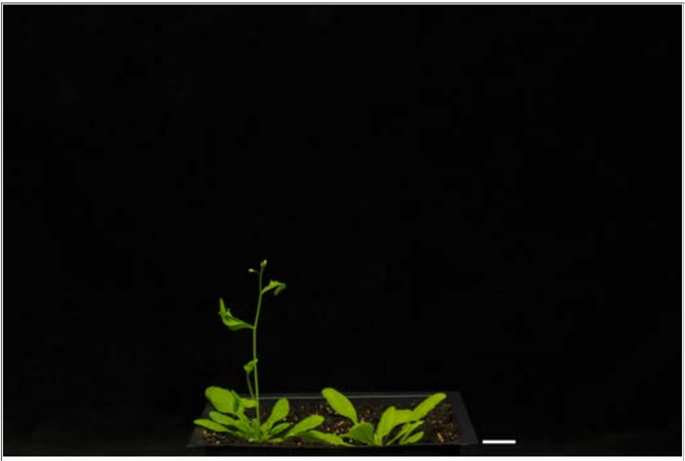

C200-67-N1

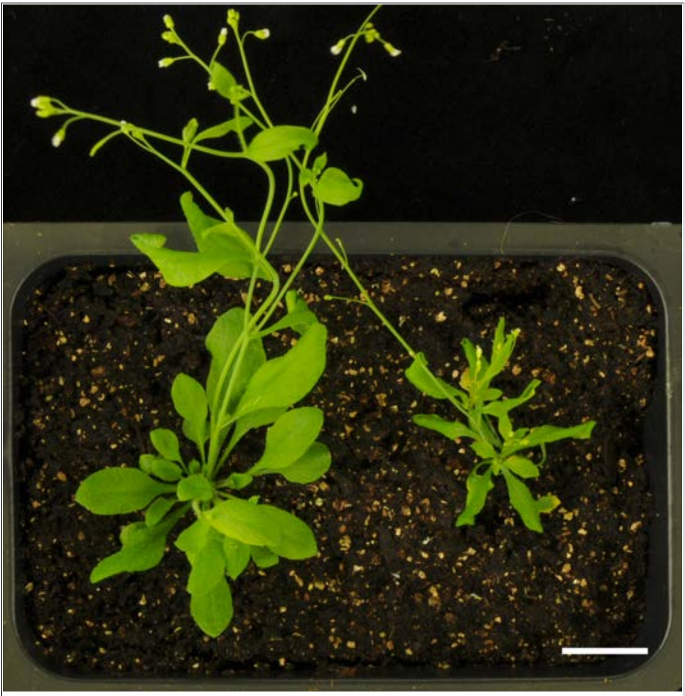
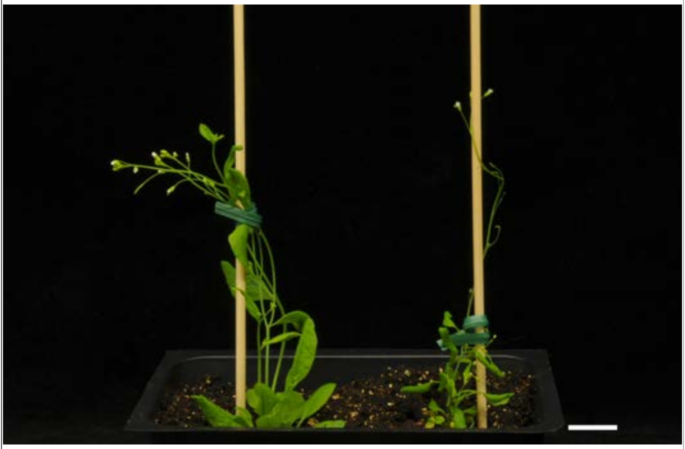

C200-68-N1

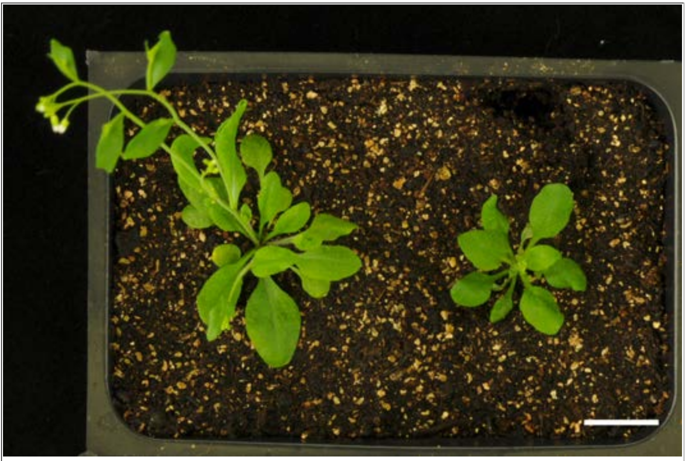

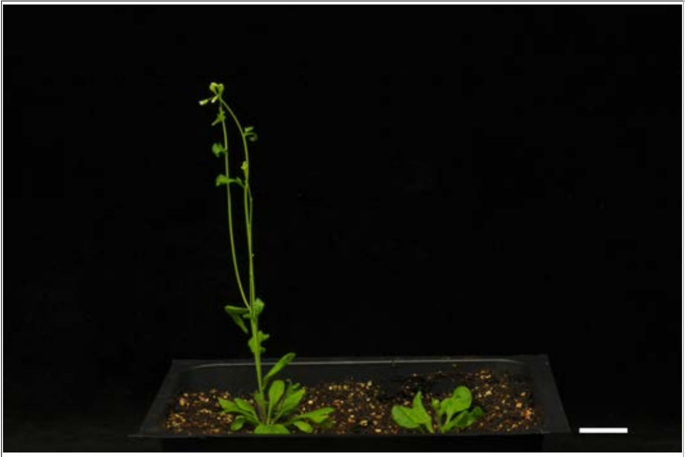

C200-68-N2

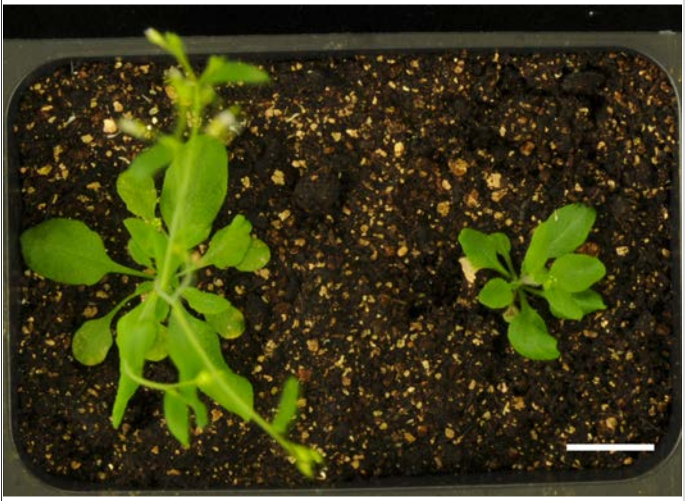

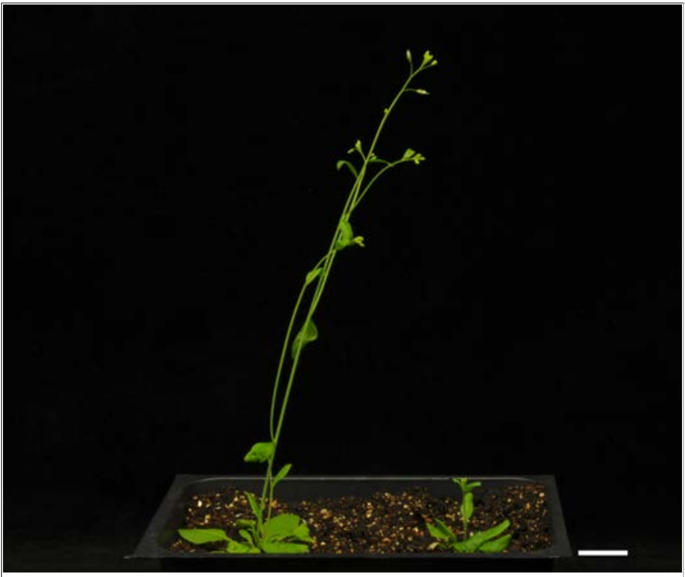

C200-70-as2

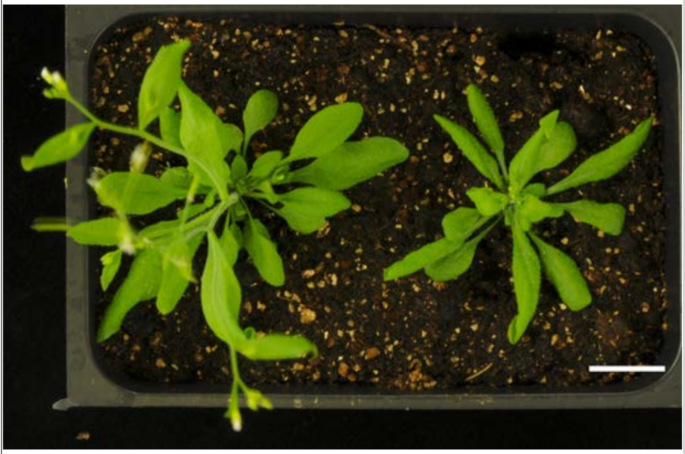

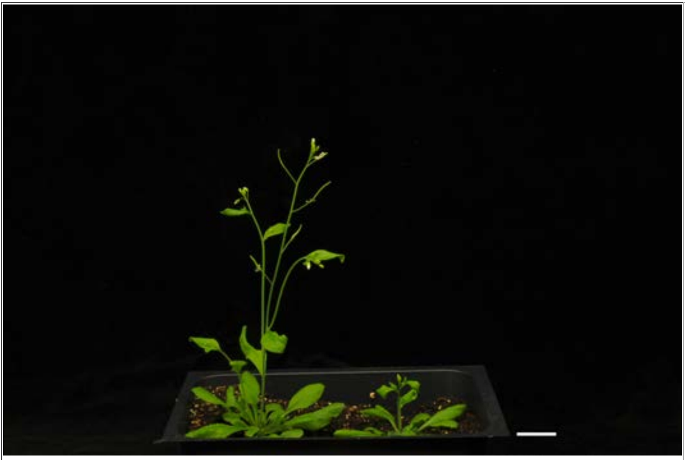

C200-74-pl1

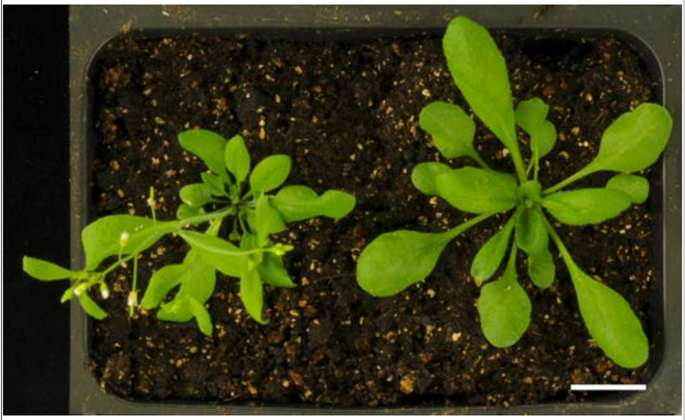

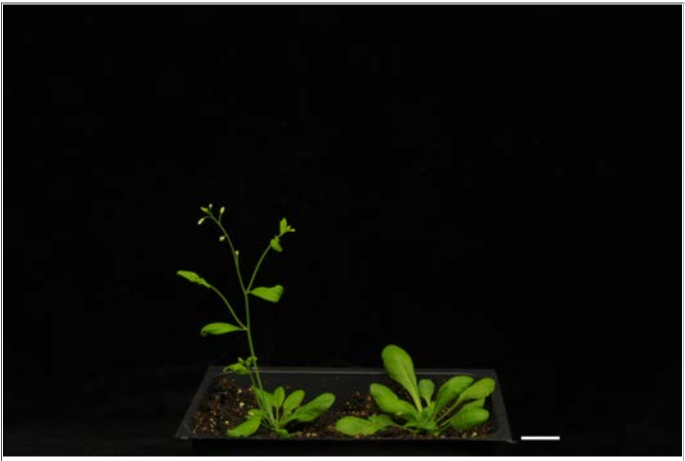

C200-78-N1

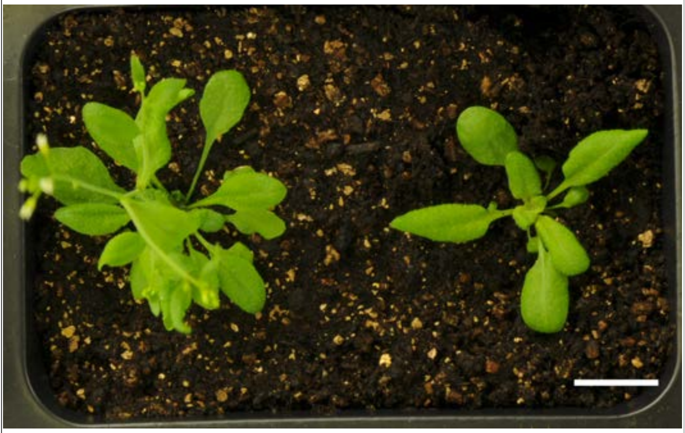

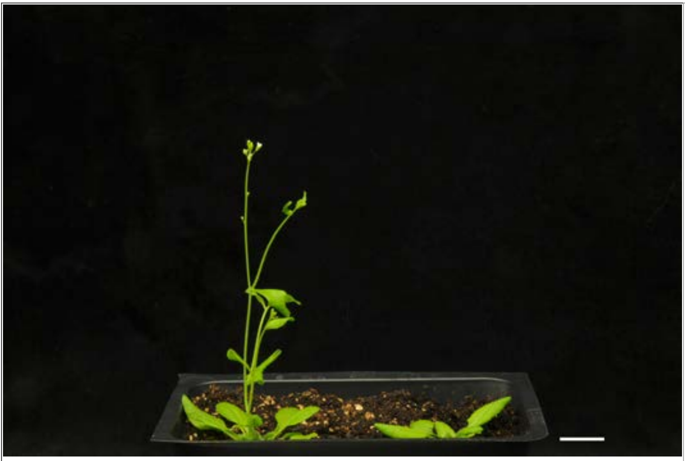

C200-87-N1

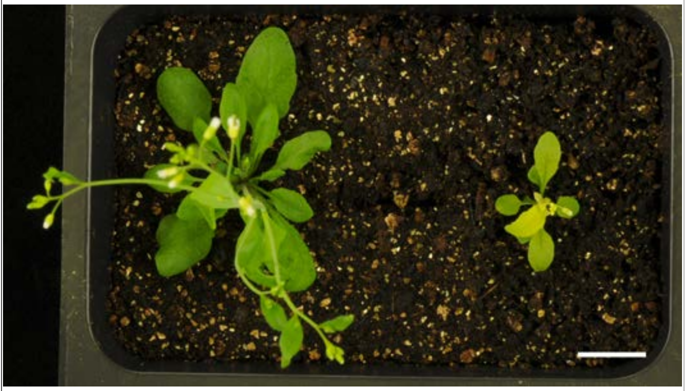

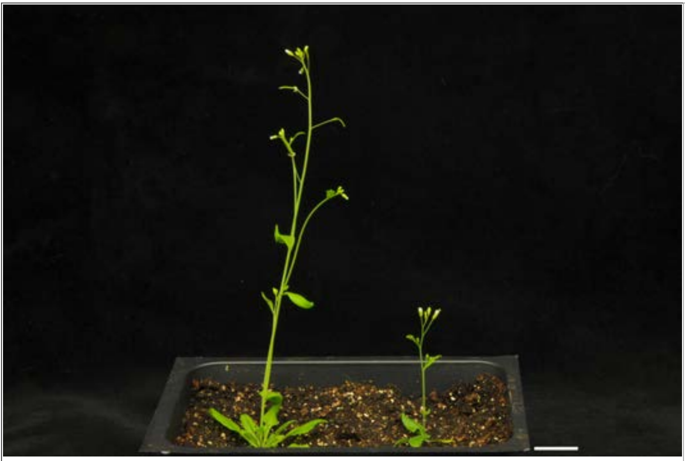

C200-87-pg1	
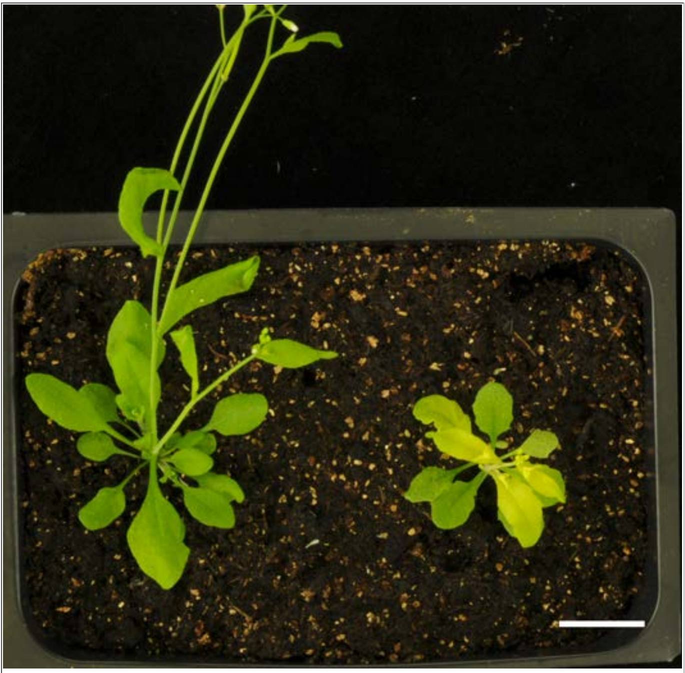	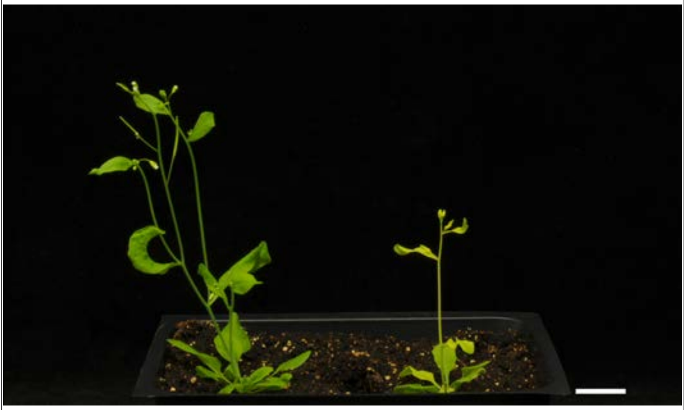

C200-90-as1	
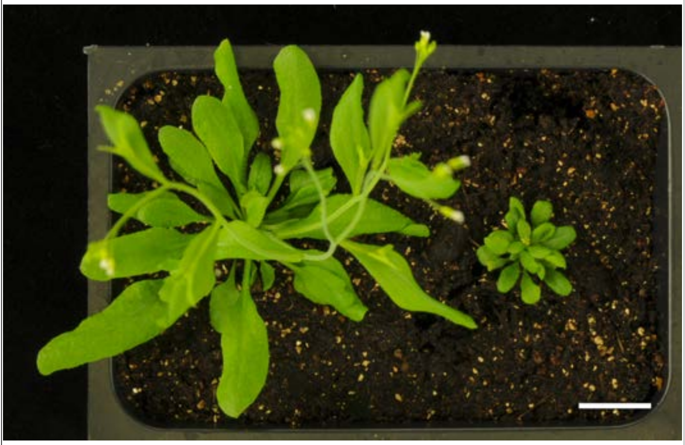	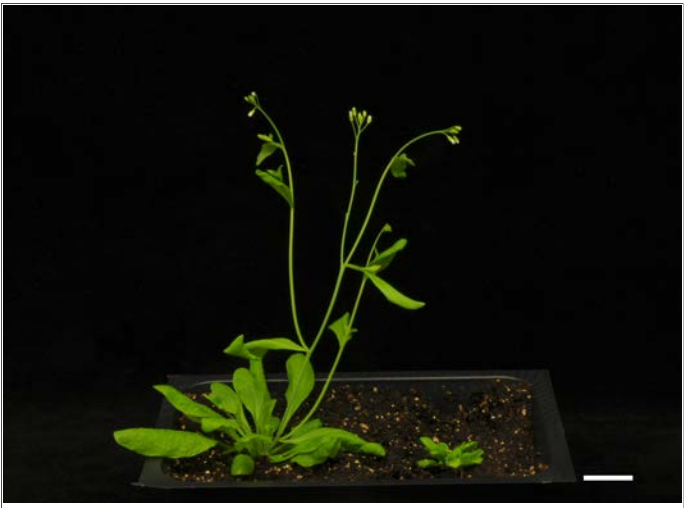

C200-91-as1	
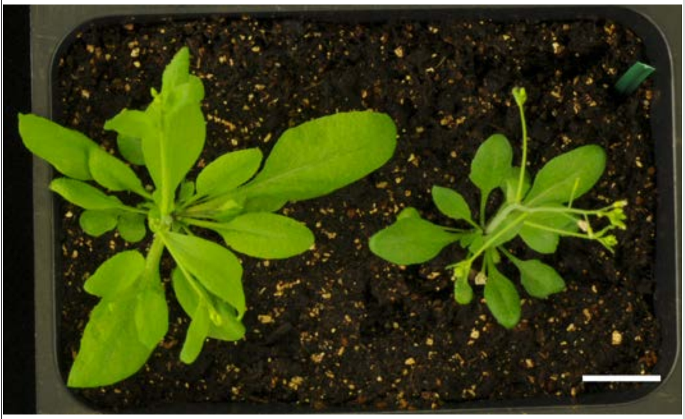	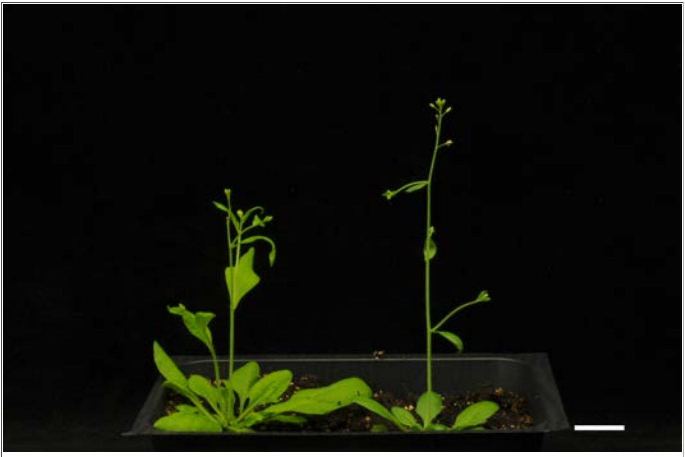

C200-94-as1

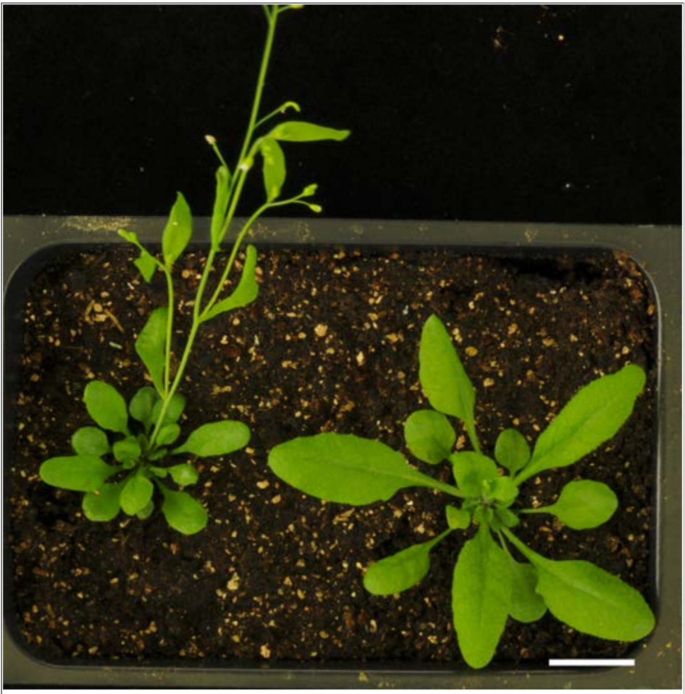

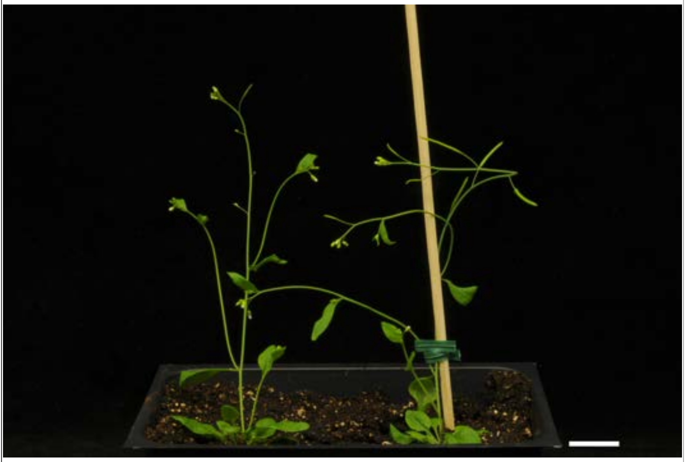

C200-98-pl1

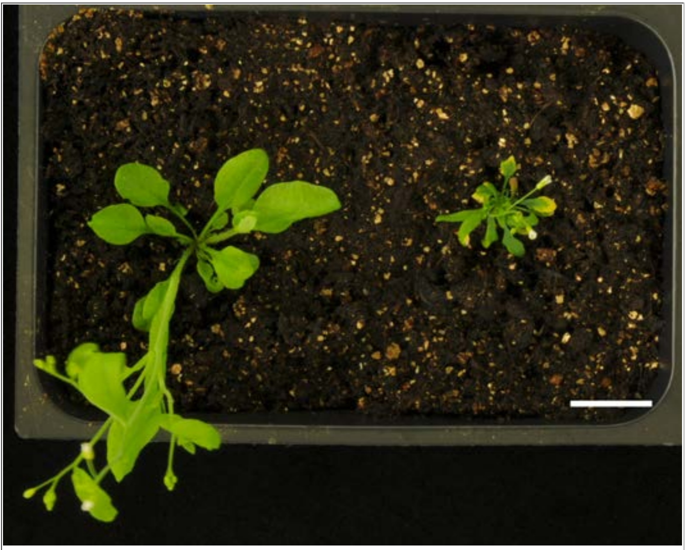

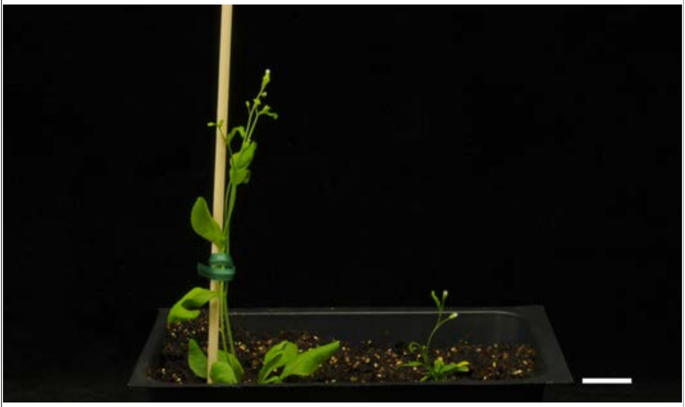

Supplementary Figure 1 **Photographs of mutants induced by Ar- and C-ion irradiations. Each mutant of the M_3 generation (right side) was grown with a wild-type plant(left side). Photographs were taken after 30 days of culture. The scale bars are 1.5 cm.**
