# Supplementary material for: Genomic view of heavy-ion-induced deletions associated with distribution of essential genes in Arabidopsis thaliana
Source: Front Plant Sci. 2024 Apr 17;15:1352564. doi: 10.3389/fpls.2024.1352564 (PMC11061394; doi:10.3389/fpls.2024.1352564)
Supplement: Supplementary file 2 [file Image_2.pdf]

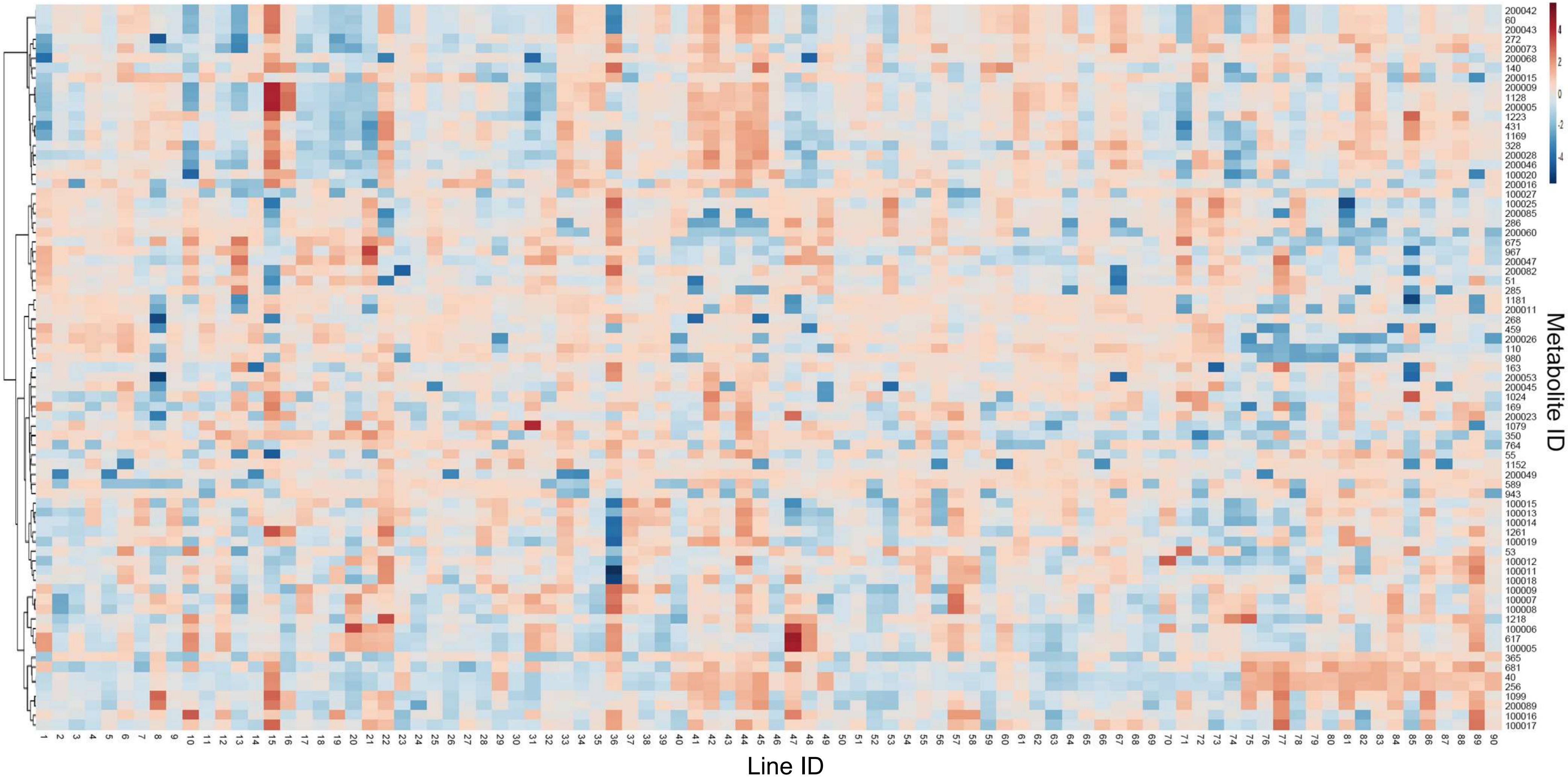

Supplementary Figure 2 Metabolic profiling of heavy-ion induced. Mean intensity of metabolite of three replicates of each mutant was indicated by the heatmap. Correspondence between IDs and names of mutant lines and metabolites are shown in Supplemental Tables 4 and 5, respectively.
